# Phylogenomic study of *Orostachys* using Angiosperms353: polyphyly of subsections with taxonomic consequences

**DOI:** 10.3389/fpls.2025.1696546

**Published:** 2025-12-12

**Authors:** Ha-Rim Lee, Halam Kang, Yoo-Jung Park, Ho-Kwon Kwak, Hyeyeon Kim, Jieun Park, Hyeok Jae Choi, Kyung-Ah Kim, Kyeong-Sik Cheon

**Affiliations:** 1 Department of Biological Science, Sangji University, Wonju, Republic of Korea; 2 Department of Biology and Chemistry, Changwon National University, Changwon, Republic of Korea; 3 Department of Biological Sciences, Kangwon National University, Chuncheon, Republic of Korea

**Keywords:** Crassulaceae, Angiosperms353, phylogenomics, target enrichment, polyphyly

## Abstract

**Introduction:**

The genus *Orostachys* (Crassulaceae) has been subject to considerable disagreement regarding its taxonomic boundaries because of extensive morphological similarities among taxa and widespread intraspecific variation. Additionally, previous phylogenetic studies based on molecular markers have consistently demonstrated that the two subsections of *Orostachys* (subsect. *Orostachys* and subsect. *Appendiculatae*) do not constitute a monophyletic group, leaving the generic boundaries unresolved to date. Therefore, we aimed to evaluate the generic boundaries and accurate taxonomic positions of individual taxa via the Angiosperms353 target enrichment approach.

**Methods:**

We analyzed a total of 43 individuals representing 25 taxa from 5 genera, including 14 *Orostachys* taxa and closely related genera such as *Hylotelephium* and *Meterostachys*. High-quality genomic data were obtained, with an average gene recovery rate of 99.1% (349.5/353 genes) and substantial sequence length recovery (average 1,223 bp per locus), resulting in concatenated datasets of 397-400 kbp sequences. Phylogenetic analyses using concatenation and coalescent-based approaches with datasets based on different threshold values were conducted.

**Results:**

Phylogenetic analyses revealed exceptionally high concordance across all phylogenetic trees, with most nodes having UFBoot values ≥95% and LPP values ≥0.95. Our findings showed the polyphyletic nature of *Orostachys*, with O. subsect. *Orostachys* forming a monophylum distinct from O. subsect. Appendiculatae and related genera. O. subsect. *Orostachys* constituted a monophyletic group rather than exhibiting a paraphyletic relationship with Hylotelephium. *O. ramosa* and *O. japonica f. polycephala* clustered with *O. malacophylla* and *O. japonica*, respectively. *O. margaritifolia*, *O. latielliptica*, *O. chongsunensis*, and *O. iwarenge f. magnus* formed independent clades.

**Discussion:**

Unlike previous studies, our Angiosperms353 data demonstrate that O. subsect. *Orostachys* forms a completely independent lineage, representing the most important finding of this study. The high support values confirm the reliability of our results and demonstrate the effectiveness of the Angiosperms353 approach in resolving complex evolutionary relationships within morphologically challenging plant groups. Our findings support treating *O. ramosa* and *O. japonica f. polycephala* as synonyms of *O. malacophylla* and *O. japonica*, respectively, while recognizing the endemic Korean species *O. margaritifolia*, *O. latielliptica*, *O. chongsunensis*, and *O. iwarenge f. magnus* as independent taxa.

## Introduction

The family Crassulaceae comprises approximately 34 genera and more than 1,400 species of succulent plants distributed worldwide, with particular diversity in the Northern Hemisphere (Thiede and Eggli, 2007). Understanding the phylogenetic relationships within this family has long been challenging because of the morphological convergence associated with succulence and complex patterns of diversification (Mort et al., 2001; Messerschmid et al., 2020). Early molecular phylogenetic studies utilizing a few plastid markers (e.g., *matK* and *trnL-F*) have established the monophyly of the major subfamilies and revealed unexpected relationships among traditionally recognized genera (Mort et al., 2001). Subsequent expanded analyses incorporating multiple plastid and nuclear ribosomal DNA markers resolved relationships among major clades and demonstrated the polyphyly of several morphologically defined genera, including *Sedum* sensu lato, necessitating significant taxonomic reorganization (Mayuzumi and Ohba, 2004; Gontcharova et al., 2006). More recently, phylogenomic approaches employing complete chloroplast genome sequences have provided greater resolution for some groups (Chang et al., 2021; Lee et al., 2022; An et al., 2023); however, fundamental questions regarding generic delimitation and species relationships remain unresolved in several lineages, particularly those that have undergone recent rapid diversification during the late Miocene (~10 Ma; Bruyns et al., 2019).

Among these problematic groups, the genus *Orostachys* Fisch. represents a particularly challenging case for phylogenetic resolution. The genus *Orostachys* comprises succulent perennials, with approximately 15 species distributed primarily across Central and Northeast Asia (Lee and Lee, 2000; Fu and Ohba, 2001). This genus was first described as an independent genus by Fischer (1809) because of its distinct growth environment from that of other genera. However, Steudel (1821); de Candolle (1828), and Bentham and Hooker (1865) did not recognize *Orostachys* as an independent genus and treated it as part of the genera *Sedum* L., *Umbilicus* DC., and *Cotyledon* Tourn. ex L., respectively. Since then, various studies (Berger, 1930; Borissova, 1939; Webb, 1964) have supported the taxonomic position of *Orostachys* as an independent genus, and it is currently recognized as such (Lee et al., 2022).

With respect to the infrageneric classification, the genus was initially divided into two sections, *Orostachys* H.Ohba and *Schoenlandia* H.Ohba, based on leaf shape, number of stamens, and inflorescence form (Ohba, 1978). Later, sect. *Schoenlandia* was described as a new genus, *Kungia* K.T.Fu, due to differences in stamen arrangement and inflorescence form (Fu, 1988); thus, only one section currently exists. In addition, section *Orostachys* comprises two subsections, *Appendiculatae* (Boriss.) H.Ohba and *Orostachys* (Boriss.) H.Ohba, depending on the presence or absence of appendages at the leaf apex (Ohba, 1978).

Despite this taxonomic stability, various previous phylogenetic studies based on molecular markers have revealed that the two subsections do not constitute a monophyletic group, which introduced uncertainty into the monophyly of the genus (Mayuzumi and Ohba, 2004; Gontcharova et al., 2006; Gontcharova and Gontcharov, 2009; Messerschmid et al., 2020). Furthermore, these studies have consistently revealed that *Orostachys* subsect. *Orostachys* has a paraphyletic relationship with the genus *Hylotelephium* H.Ohba, while *Orostachys* subsect. *Appendiculatae* shows a close affinity to *Meterostachys* Nakai, suggesting that the current generic delimitation should be fundamentally reconsidered. To solve this problem, phylogenomic studies based on complete chloroplast genome sequences have been conducted (Lee et al., 2022; An et al., 2023). However, these studies have yielded results similar to those of previous studies, leaving the issue of generic delimitation unresolved.

Chloroplast genome sequence-based phylogenetic analysis has resolved many taxonomic challenges, but it also has limitations in providing comprehensive evolutionary histories of plant lineages. Specifically, the use of chloroplast markers has three major limitations. First, they provide only maternal lineage information, preventing the detection of hybridization events involving biparental inheritance. Second, they cannot adequately represent incomplete lineage sorting (ILS), which frequently occurs during rapid species diversification. Third, they fail to capture the full complexity of nuclear genome evolution, including the rate of heterogeneity among taxa and potential cytonuclear cells (Soltis and Soltis, 1998; Jansen et al., 2007; Rose et al., 2021). Additionally, convergent evolution in chloroplast genomes occurring in independent lineages can lead to misleading phylogenetic signals, particularly in groups that have undergone adaptive radiation or experienced similar selective pressures (Gonçalves et al., 2019; Rose et al., 2021). In particular, previous studies on the genus *Orostachys* have demonstrated that the complete evolutionary relationships of chloroplast genomes alone are difficult to interpret because of these limitations (Lee et al., 2022; An et al., 2023).

To overcome these limitations, the Angiosperms353 probe set was recently developed to target 353 evolutionarily conserved single-copy nuclear genes across seed plants (Johnson et al., 2019). This universal probe set offers several critical advantages over traditional approaches: 1) it captures biparentally inherited nuclear markers that can reveal reticulation events and hybrid origins; 2) the large number of independent loci provides sufficient power to distinguish between ILS and hybridization through gene tree concordance analyses; 3) the standardized nature of the probe set enables direct comparison across studies and facilitates collaborative efforts in plant systematics; and 4) the inclusion of both exonic and flanking non-coding regions provides variation suitable for resolving relationships across multiple phylogenetic scales. The effectiveness of this approach has been demonstrated in resolving complex phylogenetic relationships in various challenging plant groups at multiple taxonomic levels, including at the order level (Zuntini et al., 2024), the family level (Larridon et al., 2021; Joyce et al., 2023), and the genus level (Meudt et al., 2024; Gagnon et al., 2022), particularly in cases with recent rapid radiation (Meudt et al., 2024; Murillo-A et al., 2022) and in groups with extensive hybridization histories (Nauheimer et al., 2021). Previous taxonomic studies of *Orostachys* have documented extensive morphological similarities among species and substantial intraspecific variation (Lee and Lee, 2000; Ohba, 2003), which have resulted in ongoing taxonomic disagreements regarding species delimitation and classification. For morphologically complex groups such as *Orostachys*, where traditional markers have failed to provide clear resolution, the Angiosperms353 approach represents an optimal solution by providing the genomic-scale data needed to disentangle complex evolutionary patterns.

Therefore, the aim of this study was to address the following questions via the Angiosperms353 target enrichment approach: 1) Is the genus *Orostachys* monophyletic, or should it be divided into separate genera based on its two subsections? 2) What is the phylogenetic relationship between *Orostachys* subsect. *Orostachys* and the genus *Hylotelephium*—are they paraphyletic as suggested by previous studies, or do they represent independent lineages? 3) How should taxonomically controversial species within *Orostachys*, including the endemic Korean taxa described by Lee and Lee (2000), be treated? By utilizing hundreds of nuclear loci that capture both coding and non-coding variations, we expected to achieve a substantially improved resolution of phylogenetic relationships and provide a robust framework for the taxonomic revision of this challenging group.

## Materials and methods

### Taxon sampling and DNA extraction

Plant material was collected from the native habitats of each taxon, and voucher specimens were deposited in the Sangji University Herbarium (SJUH) (**Supplementary Figure S1**, **Supplementary Table S1**). The sampling strategies varied according to the distribution range and the presence of taxonomic issues for each taxon. For taxa with taxonomic ambiguity regarding their systematic position or phylogenetic relationships with closely related taxa in previous studies (Kim and Park, 2005; Lee et al., 2022; An et al., 2023), two to five individuals were obtained considering their distribution range, while single individuals were collected for morphologically stable taxa. Although our sampling does not include all described *Orostachys* species due to the combination of accessibility constraints and the poor preservation quality of succulent plant DNA in herbarium specimens, we prioritized comprehensive geographic sampling of taxonomically controversial taxa to address the primary phylogenetic questions. Detailed collection information, including specimens from type localities, where accessible, is provided in **Supplementary Table S1**.

In total, 43 individuals representing 25 taxa from five genera were included in this study. This included 32 individuals of 14 *Orostachys* taxa (12 species, one forma, and one unidentified taxon), eight individuals of eight *Hylotelephium* taxa [seven species and *Sedum taquetii* Praeger, which is synonymized with *Hylotelephium verticillatum* (L.) H.Ohba in some taxonomic databases such as WFO and POWO], and one individual of the *Meterostachys* taxon. Previous phylogenetic studies have consistently shown that *Hylotelephium* exhibits a close relationship with *Orostachys* subsect. *Orostachys*, while *Meterostachys* is closely related to *Orostachys* subsect. *Appendiculatae* (Lee et al., 2022; An et al., 2023); these genera were therefore included to evaluate the generic boundaries of *Orostachys*. Two *Phedimus* Raf. taxa were selected as outgroups.

Genomic DNA was extracted using the DNeasy Plant Mini Kit (Qiagen Inc., Valencia, CA, USA) following the manufacturer’s instructions. Fresh leaf tissue (approximately 100 mg) was used for all extractions to ensure a high-quality DNA yield. The tissue was ground in liquid nitrogen immediately before extraction to prevent DNA degradation. The quality and quantity of the extracted DNA were evaluated using a LabChip GX II (PerkinElmer Inc., Waltham, MA, USA). Samples that passed the quality and quantity filters with a DNA concentration ≥20 ng/μL, a genomic quality score ≥3.0, and a purity ratio (A260/280) ≥1.80 were subsequently sent to LabGenomics (Seongnam, Korea) and Macrogen (Seoul, Korea) for library preparation.

### Library preparation, quality control, target enrichment, and sequencing

The process of library preparation followed the manufacturer's instructions (Twist Bioscience). Genomic DNA libraries were prepared from 50 ng of input gDNA using the Twist Library Preparation EF Kit (96 samples, PN 101058) with full-length combinatorial dual-index TruSeq-compatible Y-adapters (Illumina, San Diego, CA, USA). DNA quantity and quality were assessed using the PicoGreen assay and agarose gel electrophoresis, respectively. The input DNA was diluted with Elution Buffer (EB) buffer and enzymatically sheared to a target fragment size of approximately 200 bp. The fragmented DNA then underwent end repair and A-tailing, followed by ligation with Twist UDI index adapters. After ligation, the adapter-ligated fragments were purified using AMPure XP beads and amplified via limited-cycle polymerase chain reaction (PCR) (6–8 cycles).

Target enrichment was performed using the Angiosperms353 probe set, which targets 353 evolutionarily conserved, single-copy nuclear genes across angiosperms (Johnson et al., 2019). This standardized bait kit enables the capture of both exonic regions and flanking non-coding sequences, providing sufficient variation for phylogenetic resolution at multiple taxonomic levels. Following hybridization, the probe-bound DNA fragments were isolated through the use of streptavidin-coated magnetic beads, which selectively captured the biotinylated probes hybridized to the targeted DNA fragments. Stringent post-capture washes were performed to remove non-specifically bound DNA and ensure high enrichment specificity. The captured libraries were then PCR-amplified using high-fidelity polymerase enzymes under optimized cycling conditions (typically 10–12 cycles). The number of PCR cycles was minimized to reduce amplification bias while ensuring sufficient yield for sequencing.

The postcapture libraries were purified using AMPure XP beads (Beckman Coulter, Brea, CA, USA) and subjected to quantitative and qualitative assessments. Library concentration was determined via quantitative PCR (qPCR) using the KAPA Library Quantification Kit (KAPA Biosystems, Wilmington, MA, USA), strictly adhering to the manufacturer’s protocol for Illumina-compatible libraries. Fragment size distribution and overall library integrity were evaluated using the Agilent TapeStation 4200 platform with D1000 ScreenTape, confirming appropriate size profiles (typically 300–400 bp, including adapters) and the absence of primer–dimer or degradation artifacts. Only libraries meeting stringent quality thresholds were advanced to high-throughput sequencing.

Raw reads were generated on three next-generation sequencing (NGS) platforms due to platform availability during the study (**Supplementary Table S2**). Twenty-five samples were sequenced on a NovaSeq 6000 platform (Illumina Inc., San Diego, CA, USA) to produce 2 × 100 bp paired-end reads, while three samples were processed on a NovaSeq X platform (Illumina Inc., San Diego, CA, USA) to generate 2 × 150 bp paired-end reads. The remaining 15 samples were sequenced on an MGI-400 platform (MGI Tech Co., Shenzhen, China) to produce 2 × 150 bp paired-end reads.

### Data assembly, filtering, and alignment

Raw sequencing data were demultiplexed by the sequencing facilities (LabGenomics and Macrogen) according to the sample-specific index sequences. bcl2fastq v2.20 (for Illumina platforms) or equivalent software (for MGI platforms) was used for this purpose. Raw reads have been deposited in the NCBI Sequence Read Archive (SRA) under the BioProject accession PRJNA1310746, and individual SRA accession numbers for each taxon are listed in **Supplementary Table S1**. The quality of the raw sequencing reads was first assessed using FastQC v0.11.5 (Andrews, 2010). Quality filtering was performed using Trimmomatic v0.38 (Bolger et al., 2014) with the following parameters: Illumina adapter removal (ILLUMINACLIP: TruSeq3-PE.fa:2:30:10), removal of low-quality bases from both ends of reads (LEADING:3, TRAILING:3), sliding-window-based trimming (SLIDINGWINDOW:4:15, with an average quality below 15 within a 4-bp window), and minimum read-length filtering (MINLEN:36). This process resulted in high-quality paired-end reads suitable for downstream analyses.

The trimmed reads were used to extract the Angiosperms353 target loci, and consensus sequences were assembled using HybPiper v1.3.1 (Johnson et al., 2016). In the HybPiper pipeline, the reads were mapped to the target genes using the Burrows-Wheeler Aligner (BWA) (Li and Durbin, 2009), and the mapped reads were *de novo* assembled using SPAdes (Bankevich et al., 2012) with automatic k-mer selection. Gene regions were extracted from the assembled contigs using Exonerate with a protein-to-genome alignment model (–model protein2genome) to generate supercontigs containing the identified introns. The detailed commands and parameters for all bioinformatics analyses are provided in **Supplementary Method S1**. Paralog detection was performed using the HybPiper stats module, which identifies genes with multiple contigs exceeding a specified proportion of the reference length. Genes with paralog warnings were excluded from downstream analyses to retain putative single-copy orthologs. Specifically, genes were removed if they met both of the following criteria: 1) the presence of multiple contigs ≥75% of the target reference length and 2) anomalous depth coverage patterns indicative of gene duplication. A detailed summary of the paralogs recovered during this process is presented in **Supplementary Table S3**. Supercontigs (exons plus flanking non-coding regions) for each target gene were retrieved using the retrieve_sequences.py script from HybPiper and were used for all subsequent analyses. Only supercontigs were utilized because recent studies have demonstrated that flanking non-coding regions contain substantially more phylogenetically informative variation than conserved coding sequences, and this increased variation is more effective for resolving relationships among recently diverged and morphologically similar taxa (Johnson et al., 2019; Slimp et al., 2021; Bagley et al., 2020).

The retrieved sequences were aligned by locus using MAFFT v7.490 (Katoh and Standley, 2013) with automatic algorithm selection and parallel processing. Alignments were filtered based on minimum sequence length and taxon occupancy thresholds. Prior to phylogenetic analysis, the alignments were trimmed using trimAl v1.4 (Capella-Gutiérrez et al., 2009) with the following parameters to improve phylogenetic signal by removing gap-rich regions and poorly conserved, ambiguously aligned positions: columns with >80% gaps were removed (-gt 0.8), and columns with similarity scores <0.001 were removed (-st 0.001). For concatenation-based analyses, trimmed gene alignments were concatenated into a supermatrix using AMAS (Borowiec, 2016), which generated a partition file defining gene boundaries.

### Phylogenetic analyses

Phylogenetic analyses were performed using supercontig datasets derived from Angiosperms353 target genes. To assess the effect of alignment quality on phylogenetic inference, the datasets were filtered to include loci whose gap proportions were less than 30%, 50%, and 70%, which were selected to balance locus inclusion maximization, moderate filtering, and alignment quality prioritization, respectively. Since moderate amounts of missing data have been shown not to significantly affect phylogenetic accuracy in coalescent-based methods (Xi et al., 2014; Streicher et al., 2016), analyses were performed with this consideration.

The best-fit nucleotide substitution model for each locus was selected using jModelTest v2.1.10 (Darriba et al., 2012) based on the Bayesian information criterion (BIC). For concatenation-based analyses, maximum likelihood (ML) trees were reconstructed from the concatenated supermatrix using partitioned analysis in IQ-TREE (Minh et al., 2020), where each gene was treated as a separate partition with its corresponding best-fit model. Node support was assessed using 1,000 ultrafast bootstrap replicates (Hoang et al., 2018).

For multi-locus species tree estimation, ML gene trees for each locus were inferred using IQ-TREE under the corresponding best-fit substitution model with bootstrap support. Prior to ASTRAL analysis, all bootstrap support values and branch lengths were removed from the gene trees using the Phylo module of BioPhthon (Cock et al., 2009), which retained only topological information (Zhang et al., 2018). The cleaned gene trees were used as input for ASTRAL-III v5.7.8 (Zhang et al., 2018) to estimate the multi-locus species tree, with internal branch support assessed using local posterior probabilities (LPPs). ASTRAL analyses were performed for the supercontig datasets and IQ-TREE gene trees at each gap threshold (30%, 50%, and 70%).

To assess the robustness of the phylogenetic inferences, the concordance between the concatenated ML trees and multi-locus species trees was proved by comparing the topological consistency at internal nodes across all different gap threshold datasets. Additionally, gene tree concordance with the multi-locus species tree was quantified through quartet frequency analysis in ASTRAL. Quartet support values were visualized as pie charts on tree nodes using the R package “ggtree” v3.10.0 (Yu et al., 2017) and were integrated into the phylogenetic tree figures. Detailed commands and parameters for all bioinformatics analyses are provided in **Supplementary Method S1**.

## Results

### Sequencing and target capture efficiency

The NGS and target capture results for 43 individuals representing 25 taxa are summarized in **Table 1**. Sequencing generated a total of 176.3 million paired-end reads, with an average of 4.1 million read pairs per sample (range, 1.6–5.9 million). The quality of the raw sequencing data was consistently high, with an average of 88.9% of the reads per sample achieving Phred scores > Q30 (range, 75.4%–95.3%).

**Table 1:** Summary of NGS and target capture results in this study.

Sample	Raw reads	Bases ≥ Q30 (%)	Cleaned reads	Number of reads on target	Number of genes with reads	Number of genes with contigs	Number of genes with sequences	Recovery sequences (%)
*Orostachys boehmeri*	46,602,684	95.0	28,969,768	1,884,414	353	332	311	88.1
*Orostachys chongsunensis*	40,316,020	77.1	38,229,260	1,245,430	349	274	272	77.1
*Orostachys iwarenge_1*	33,660,822	76.9	31,768,044	752,647	348	271	264	74.8
*O. iwarenge_2*	54,296,774	94.4	50,194,400	16,253,994	352	301	308	87.3
*O. iwarenge* f. *magnus_1*	50,881,472	94.1	48,734,832	13,700,108	350	302	307	87.0
*O. iwarenge* f. *magnus_2*	38,819,808	76.8	36,781,000	1,257,664	349	275	271	76.8
*Orostachys japonica_1*	38,887,966	76.1	36,681,768	3,235,685	341	299	289	81.9
*Orostachys japonica_2*	49,366,384	94.4	47,056,466	12,437,675	353	301	312	88.4
*Orostachys japonica_3*	56,062,316	94.1	56,565,580	11,228,052	353	306	308	87.3
*Orostachys japonica_4*	60,728,570	93.9	53,327,968	13,815,756	352	299	308	87.3
*Orostachys japonica* f. *polycephala_1*	27,075,950	74.8	25,306,014	3,362,641	331	315	291	82.4
*O. japonica* f. *polycephala_2*	49,534,638	94.3	45,409,824	12,391,334	353	295	309	87.5
*O. japonica* f. *polycephala_3*	52,284,616	94.3	45,952,164	9,036,551	353	298	310	87.8
*O. japonica* f. *polycephala_4*	49,005,178	94.0	47,368,496	13,778,067	353	302	311	88.1
*Orostachys latielliptica*	59,119,504	94.2	55,163,870	11,501,149	352	292	309	87.5
*Orostachys margaritifolia*	50,285,388	76.3	47,518,774	2,395,027	351	287	285	80.7
*Orostachys malacophylla_1*	57,695,646	94.5	46,525,650	1,776,315	351	281	281	79.6
*Orostachys malacophylla_2*	55,671,382	93.9	50,710,918	1,502,438	353	280	290	82.2
*Orostachys malacophylla_3*	50,715,678	94.4	57,262,886	10,328,371	353	291	300	85.0
*Orostachys malacophylla_4*	61,751,346	95.1	51,570,726	1,646,818	353	284	297	84.1
*Orostachys malacophylla_5*	49,044,872	77.3	40,877,404	1,241,680	353	277	288	81.6
*Orostachys minuta_1*	33,169,904	75.2	56,893,294	13,439,247	353	295	312	88.4
*Orostachys minuta_2*	62,911,778	94.5	31,143,776	3,884,398	337	310	298	84.4
*Orostachys minuta_3*	59,952,796	93.8	55,843,032	13,955,377	353	305	314	89.0
*Orostachys minuta_4*	59,976,392	94.0	59,559,470	13,968,833	353	309	317	89.8
*Orostachys ramosa_1*	31,440,552	76.2	54,690,124	15,305,815	350	305	305	86.4
*Orostachys ramosa_2*	51,184,102	94.5	47,956,694	14,230,664	351	307	310	87.8
*Orostachys ramosa_3*	56,535,378	93.7	29,683,298	2,096,747	342	289	276	78.2
*Orostachys ramosa_4*	49,979,758	94.2	49,202,784	8,014,482	353	294	305	86.4
*Orostachys* sp.	50,486,892	93.6	48,828,524	13,316,291	352	301	307	87.0
*Orostachys* sp*inosa*	46,974,166	92.3	16,785,938	1,269,829	353	339	324	91.8
*Orostachys thyrsiflora*	49,454,342	93.2	19,568,558	1,580,996	353	347	319	90.4
*Meterostachys sikokianus*	25,730,414	76.5	24,319,556	2,965,328	341	306	287	81.3
*Hylotelephium erythrostictum*	20,755,010	75.7	19,552,022	1,831,913	329	313	290	82.2
*Hylotelephium pallescens*	60,541,536	95.3	53,059,670	12,928,990	353	306	313	88.7
*Hylotelephium spectabile*	49,696,926	94.5	39,322,550	6,836,171	353	313	330	93.5
*Hylotelephium ussuriense*	42,654,274	95.3	39,424,260	14,597,144	351	308	309	87.5
*Hylotelephium verticillatum*	23,997,346	75.4	22,583,856	2,734,138	335	303	284	80.5
*Hylotelephium viridescens*	49,877,166	94.4	37,709,402	5,060,603	353	320	325	92.1
*Hylotelephium viviparum*	64,315,514	94.9	42,134,406	14,336,896	353	298	305	86.4
*Sedum taquetii*	31,815,502	77.4	30,134,752	608,418	347	273	267	75.6
*Phedimus aizoon*	40,349,238	96.5	31,902,524	14,299,312	353	349	332	94.1
*P. aizoon* var. *floribundus*	34,577,022	96.4	26,659,780	12,027,885	353	349	331	93.8

NGS, next-generation sequencing.

FastQC analysis revealed no significant quality issues, with the Guanine-Cytosine (GC) content ranging from 39.8% to 44.8% (mean = 42.4%), which is consistent with that of typical plant nuclear DNA. No significant adapter contamination or overrepresented sequences were detected in any sample. The read length distribution after trimming showed minimal degradation, with 95.7% of the reads maintaining their original read lengths.

HybPiper analysis successfully recovered target sequences for an average of 349.5 out of 353 Angiosperms353 loci per sample (99.1% capture success). Gene recovery rates varied among samples, ranging from 93.2% to 100.0% of the 353 target loci (mean = 98.9%, SD = ± 1.8%), despite the use of only fresh tissue material (**Supplementary Figure S2**).

The recovered target sequences showed substantial length variation among loci, ranging from 160 to 4,540 bp, with a mean length of 1,223 bp per locus. On average, the recovered sequences represented 86.2% of the expected target length across all the samples, with individual sequence length recovery rates varying from 74.8% to 94.1% (mean = 85.5%, SD = ± 4.5%). The captured sequences differed in recovery rates between sequence types: exon regions exhibited higher recovery rates (mean = 92.3%) than flanking intron sequences (mean = 81.5%).

After sequence assembly, alignment, and quality filtering, the supercontig datasets were filtered to include loci recovered in at least 30%, 50%, and 70% of the sampled taxa, resulting in three matrices of varying completeness and size: the 30% threshold dataset comprised 332 loci with a total concatenated length of 397,543 bp, the 50% threshold dataset comprised 335 loci with a total concatenated length of 399,303 bp, and the 70% threshold dataset comprised 337 loci with a total concatenated length of 400,823 bp (**Table 2**; **Supplementary Tables S4**-**S6**).

**Table 2:** Summary statistics of the phylogenetic dataset across different individual completeness thresholds.

Completeness threshold (%)	Number of loci	Total concatenated length (bp)	Proportion of informative sites (%)
30	332	397,543	53.1
50	335	399,303	52.9
70	337	400,823	52.9

### Phylogenetic relationships

**Figure 1:**
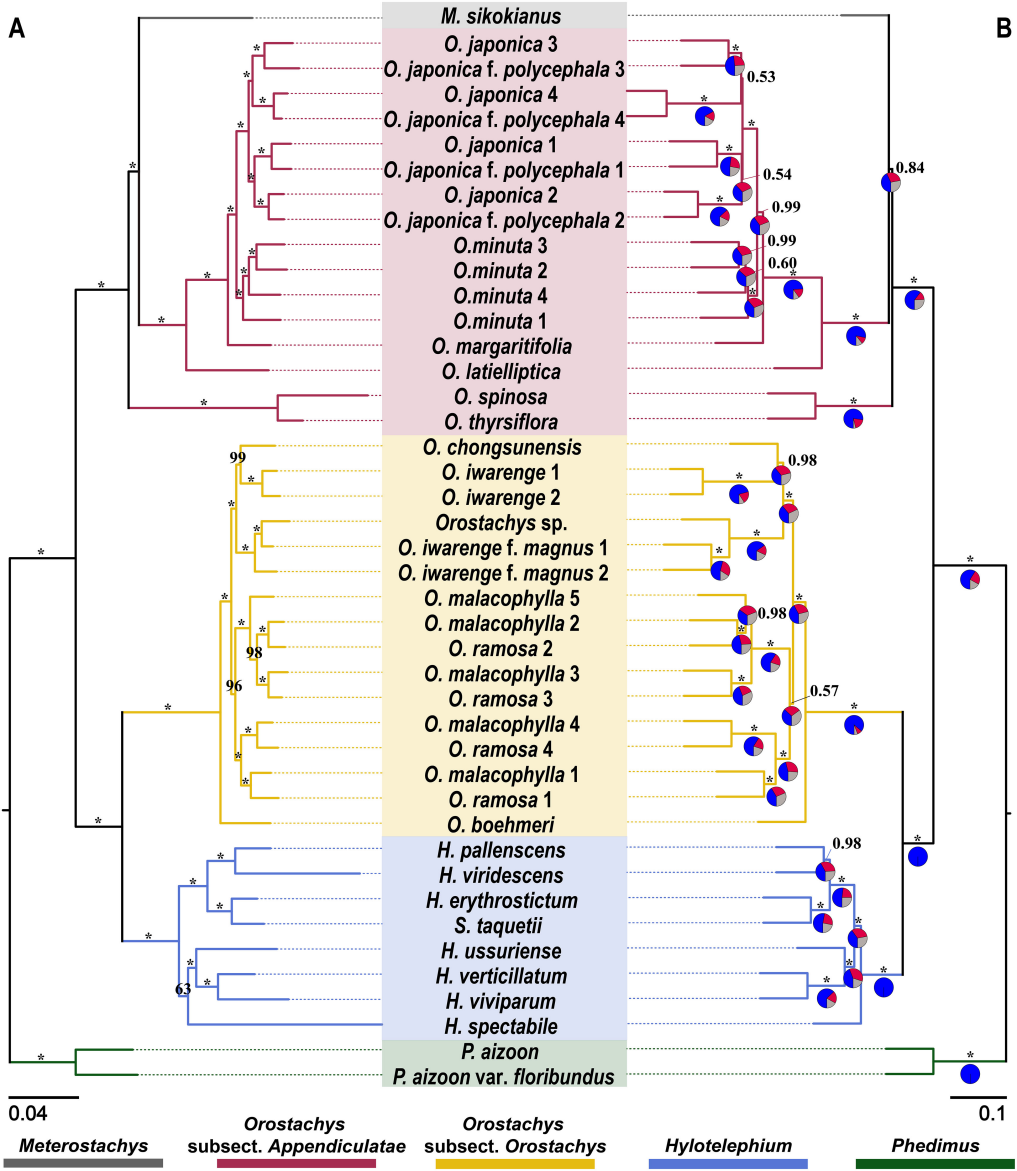
Phylogenetic trees based on a 50% completeness threshold dataset. **(A)** Concatenated maximum likelihood (ML) tree from concatenated analysis. The numbers at the nodes represent UFBoot support values, and the scale bar indicates substitutions per site. **(B)** Multi-locus species tree inferred using ASTRAL-III with LPP values. The numbers at the nodes represent LPP values, and the scale bar indicates coalescent units. Asterisks (*) indicate 100% UFBoot support in the concatenated ML tree and 1.00 LPP support in the multi-locus species tree. The pie charts at each node represent the number of gene trees that fall into one of three categories: concordant with the multi-locus species tree (blue), discordant with the multi-locus species tree (red for all other alternatives), or uninformative (gray). LPP, local posterior probability.

Phylogenetic inference was highly consistent across both analytical methods (concatenation and coalescence) and all datasets. The analyses demonstrated exceptional congruence: identical topologies were recovered for the concatenated ML trees across all three supercontig datasets (30%, 50%, and 70% completeness), and the multi-locus species trees similarly maintained congruent topologies across the three datasets (**Figure 1**; **Supplementary Figures S3**-**S6**). Furthermore, when the methods were compared, the topologies recovered by the concatenated ML tree and multi-locus species tree approaches were nearly identical. The majority of nodes in both the concatenated ML tree and the multi-locus species trees were strongly supported by high UFBoot values and LPP values, respectively. This dual concordance confirmed that the genus *Hylotelephium* formed a monophyletic group, with *S. taquetii* clustering within this clade. However, this inference is based on limited taxon sampling, as our study included only eight accessions representing eight taxa out of the approximately 30–35 currently accepted species in the genus. The two subsections of *Orostachys* formed a polyphyletic group across all trees, strongly supported by high support values. *Meterostachys* clustered within the *Orostachys* subsect. *Appendiculatae* clade. While this relationship received strong support in the concatenated ML tree (UFBoot = 100%), it showed moderate support in the multi-locus species tree (LPP = 0.84) with high gene tree discordance at this node as indicated by the quartet frequency analysis (**Figure 1**; **Supplementary Figures S3**-**S6**).

At the species level, the majority of taxa were strongly supported as monophyletic. However, several species complexes exhibited polyphyletic patterns, such as *Orostachys malacophylla* (Pall.) Fisch and *Orostachys ramosa* Y.N.Lee, which were intermixed. Similarly, *Orostachys japonica* (Maxim.) A.Berger and *O. japonica* f. *polycephala* (Makino) H.Ohba showed polyphyletic clustering.

Despite the overall concordance between the concatenated ML tree and multi-locus species tree analyses, one notable discrepancy was observed in the phylogenetic placement of *Orostachys iwarenge* f. *magnus* (**Figure 1**; **Supplementary Figures S3**-**S6**). In multi-locus species tree analyses, the two individuals of *O. iwarenge* f. *magnus* Y.N.Lee formed a single monophyletic group and exhibited a sister-group relationship with an as-yet-unidentified *Orostachys* species collected from Punggok-ri, Samcheok-si, Korea. In contrast, concatenated ML trees revealed a different arrangement in which *O. iwarenge* f. *magnus* collected from Sadong-ri, Ulleung-gun, Korea (individual 2) formed a sister group with the remaining two individuals (*O. iwarenge* f. *magnus*, individual 1, and the unidentified species).

### Phylogenetic concordance and robustness

To assess the reliability of our phylogenetic inferences, we conducted comprehensive concordance analyses across three analytical dimensions. First, we compared topologies derived from the three supercontig datasets with different completeness thresholds (30%, 50%, and 70%). Concatenated maximum likelihood analyses of these three datasets yielded perfectly identical topologies for all major clades and species-level relationships. Similarly, multi-locus species tree analyses produced identical topologies across all three datasets, demonstrating that our phylogenetic inferences were robust to variations in data completeness. Second, we compared the topologies between the concatenated ML tree and the multi-locus species tree. These two methods revealed nearly perfect topological agreement, with 41 of 42 internal nodes (97.6%) showing identical relationships (**Figure 1**, **Supplementary Figures S3**-**S6**). Third, we evaluated the concordance between individual gene trees and the multi-locus species tree using quartet frequency analysis in ASTRAL. The quartet support values, visualized as pie charts on tree nodes (**Figure 1**; **Supplementary Figures S3**-**S6**), revealed varying levels of gene tree concordance across different nodes. While the majority of nodes showed strong concordance, some nodes exhibited substantial gene tree discordance.

The UFBoot values from the concatenated ML tree and the LPP values from the multi-locus species tree analyses also indicated remarkable consistency across the datasets, demonstrating the statistical robustness of our phylogenetic hypotheses. For the UFBoot values, only six, one, and two nodes fell below 95% in the 30%, 50%, and 70% datasets, respectively. Similarly, for the LPP values, seven, five, and four nodes had values less than 0.95 in the three datasets. Notably, nodes with lower support values were predominantly associated with the taxonomically problematic species complexes described above (e.g., between *O. japonica* and *O. japonica* f. *polycephala* and between *O. malacophylla* and *O. ramosa*) rather than reflecting uncertainty in the major phylogenetic relationships.

The consistency of the phylogenetic results across the tested datasets indicates that the phylogenetic signal remains congruent despite varying levels of missing data. This exceptionally high level of concordance indicates that the Angiosperms353 dataset provides robust and reliable results for interpreting phylogenetic relationships within *Orostachys* and among its allied genera, supporting the reliability of both supermatrix and multi-locus species tree approaches for resolving relationships in this group.

## Discussion

### Angiosperms353: data quality and performance

Our study achieved high sequencing quality (with an average of 88.9% of reads with Phred scores > Q30) and substantial sequence length recovery (with an average of 1,223 bp per locus), resulting in a total concatenated length of 397–400 kbp, including flanking regions. This represents a 14%–15% increase compared with the median recovery reported by Johnson et al. (2019) (total: 349 kbp, of which 137 kbp are coding regions + 212 kbp are flanking non-coding regions). While this value does not reach the maximum recovery achieved by the best-performing taxa in their study (approximately 462 kbp), this level of recovery provides adequate data for comprehensive phylogenomic analyses.

The higher recovery rates observed in exon regions (92.3%) than in flanking non-coding sequences (81.5%) can be attributed to the probe design strategy. Since the Angiosperms353 probes were specifically designed to target coding regions, the recovery of exons represents on-target capture with high efficiency. In contrast, the recovery of flanking non-coding regions represents off-target capture, which occurs when DNA fragments extending beyond the targeted coding sequences are captured during the enrichment process (Johnson et al., 2019). Despite being off-target, these flanking regions provide valuable phylogenetic information and contributed to the substantial sequence length recovery achieved in this study (with an average of 1,223 bp per locus). The recovered loci exhibited considerable length variation (160–4,540 bp), encompassing both highly conserved and highly variable regions.

We achieved an exceptional average gene recovery rate of 99.1% (349.5/353 genes) and consistent gene recovery rates among samples (93.2%–100.0%, mean = 98.9%, SD = ± 1.8%), which represents excellent performance compared with those of other Angiosperms353 studies (Johnson et al., 2019; Meudt et al., 2024; Murillo-A et al., 2022). Our recovery rate substantially exceeds rates reported in other Angiosperms353 studies at similar taxonomic scales, including the study that developed the Angiosperms353 probe kit (80.2%; Johnson et al., 2019) and recent genus-level phylogenomic studies of rapid radiation (Meudt et al., 2024; Murillo-A et al., 2022). The superior recovery rate observed here can be attributed to the use of fresh tissue material and optimized library preparation methods, supporting the findings of Slimp et al. (2021) and Brewer et al. (2019) that sample quality and genomic library concentration significantly influence gene recovery performance. Dataset robustness is demonstrated by the minimal variation in locus counts across different completeness filtering thresholds (332, 335, and 337 loci for the 30%, 50%, and 70% thresholds, respectively), indicating that high gene recovery success minimized the impact of missing data and provided stable datasets regardless of filtering stringency. More significantly, our study demonstrates strong congruence of topologies between the concatenated ML tree and the multi-locus species tree at shallow phylogenetic scales. This concordance across fundamentally different analytical approaches provides robust support for the phylogenetic relationships within *Orostachys* and among its allied genera, demonstrating the effectiveness of the Angiosperms353 approach for resolving recent, rapid radiations in this group. We would like to emphasize that we opted for supercontigs because they have been shown to be more effective in resolving recent, rapid species radiations (Thomas et al., 2021). No phylogenetic reconstruction based solely on exons was performed here, and we cannot rule out the possibility that such a dataset could lead to a different topology similar to those of previous studies (Lee et al., 2022; An et al., 2023), given that exons have lower evolutionary rates than flanking introns (Bagley et al., 2020).

### Polyphyly of *Orostachys* and its taxonomic implications

Our results confirm that the two subsections of *Orostachys* exhibit polyphyly: *Orostachys* subsect. *Appendiculatae* is closely related to *Meterostachys* and *Orostachys* subsect. *Orostachys* shows close phylogenetic relationships with *Hylotelephium*. These findings conform with those of multiple previous studies: Mayuzumi and Ohba (2004) first revealed, using Internal Transcribed Spacer (ITS) sequences, that the two subsections of *Orostachys* do not comprise a monophyletic group, with subsequent studies using chloroplast and nuclear markers confirming this pattern (Gontcharova et al., 2006; Gontcharova and Gontcharov, 2009). Recent phylogenomic studies using complete chloroplast genome analyses have further confirmed this polyphyletic arrangement (Lee et al., 2022; An et al., 2023).

However, our study, which included more extensive taxon sampling with additional *Orostachys* species and multiple individuals per species from different geographic locations, to better capture intraspecific variation, reveals novel and significant findings that differ from those of previous works (Lee et al., 2022; An et al., 2023). While earlier studies consistently showed that *Orostachys* subsect. *Orostachys* has a paraphyletic relationship with *Hylotelephium*, our Angiosperms353 data demonstrate that *Orostachys* subsect. *Orostachys* forms a completely independent clade that is distinct from both *Orostachys* subsect. *Appendiculatae* within the same genus and the closely related genera *Hylotelephium* and *Meterostachys*. This relationship received notable statistical support, with UFBoot values of 100% and LPP values of 1 across all six phylogenetic trees (concatenated ML trees and multi-locus species trees constructed for each of the 30%, 50%, and 70% threshold datasets). Furthermore, ASTRAL analysis revealed high gene tree concordance for the main tree topology at the 30%, 50%, and 70% thresholds with values of 0.91, 0.86, and 0.91, respectively, confirming that this clustering pattern is clearly evident in the majority of individual gene trees.

The monotypic genus *Meterostachys* has historically been treated as part of the genus within *Orostachys* (Ohwi, 1953), but later studies have recognized it as an independent genus (Mayuzumi and Ohba, 2004; Gontcharova et al., 2006; Messerschmid et al., 2020; Lee et al., 2022; An et al., 2023). In our study, *Meterostachys* was clustered within the *Orostachys* subsect. *Appendiculatae* clade, a finding that deviates from the majority of previous phylogenetic reconstructions but which corroborates the topology reported by Nikulin et al. (2015). These results suggest that the existing taxonomic system requires revision and support Ohwi’s (1953) classification. However, as noted by Nikulin et al. (2015), *Meterostachys* and *Orostachys* subsect. *Appendiculatae* differ substantially in appearance, with no clear morphological similarities except for the rosette life form and cartilaginous cuspidate leaf tips. The substantial morphological differences between these taxa, particularly in terms of inflorescence morphology (bracteate cymose in *Meterostachys* versus raceme to paniculate in *Orostachys*) and carpel structure, represent significant taxonomic characteristics that warrant generic distinction. Furthermore, the chloroplast genome of *Meterostachys* has a unique structure with an inversion of the *trnS(GCU)*-*trnS(GGA)* gene block (approximately 37,000 bp) compared to that of *Orostachys* subsect. *Appendiculatae* species (Lee et al., 2022), providing compelling genomic evidence for its independent evolutionary trajectory. Given these characteristics, we believe that *Meterostachys* should continue to be recognized as an independent genus, although further studies incorporating broader geographic sampling of both *Meterostachys* and *Orostachys* subsect. *Appendiculatae* taxa, detailed comparative morphological analyses, and ontogenetic studies of inflorescence development will be necessary to definitively resolve this taxonomic question.

### Species delimitation and taxonomic recommendations based on phylogenetic inference

The genus *Orostachys* contains numerous taxonomically difficult taxa because of morphological similarities and extensive intraspecific variation, resulting in ongoing disagreements among taxonomists with respect to taxonomic identity and phylogenetic positions that remain unresolved.

The most notable taxonomic controversy concerns Ohba’s (2003) treatment of five taxa that were described by Lee and Lee (2000) as synonyms without proper taxonomic investigation: *O. ramosa* and *Orostachys chongsunensis* Y.N.Lee as synonyms of *O. malacophylla*, *Orostachys latielliptica* Y.N.Lee and *Orostachys margaritifolia* Y.N.Lee as synonyms of *O. japonica*, and *O. iwarenge* f. *magnus* Y.N.Lee as a synonym of *O. iwarenge* (Makino) H.Hara. These five taxa described by Lee and Lee (2000) have been represented in a previous study that is based on chloroplast genomes (Lee et al., 2022), which agreed with Ohba’s (2003) opinion regarding *O. ramosa* but argued that *O. iwarenge* f. *magnus* and *O. margaritifolia* should be recognized as independent taxa. Additionally, they suggested that *O. chongsunensis* should be appropriately treated as a synonym of *O. malacophylla* rather than *O. japonica*, and that *O. latielliptica* requires further research to clarify its taxonomic position. Our results fully support the previous claims regarding *O. ramosa* and *O. margaritifolia*, but show somewhat different results for the remaining taxa. We attribute these differences to the higher resolution of the Angiosperms353 method, which reflects parental inheritance, compared with that of chloroplasts, which exhibit only maternal inheritance. Therefore, our results are considered more reliable than those of previous studies. Furthermore, the observed phylogenetic discordance between the nuclear-based (Angiosperms353) and chloroplast-based topologies suggests that a history of hybridization or introgression cannot be ruled out for these taxa. While our current results clarify taxonomic boundaries using nuclear markers, future research employing population genetic approaches is warranted to comprehensively analyze patterns of gene flow and potential hybrid origins within the genus *Orostachys*.

**Figure 2:**
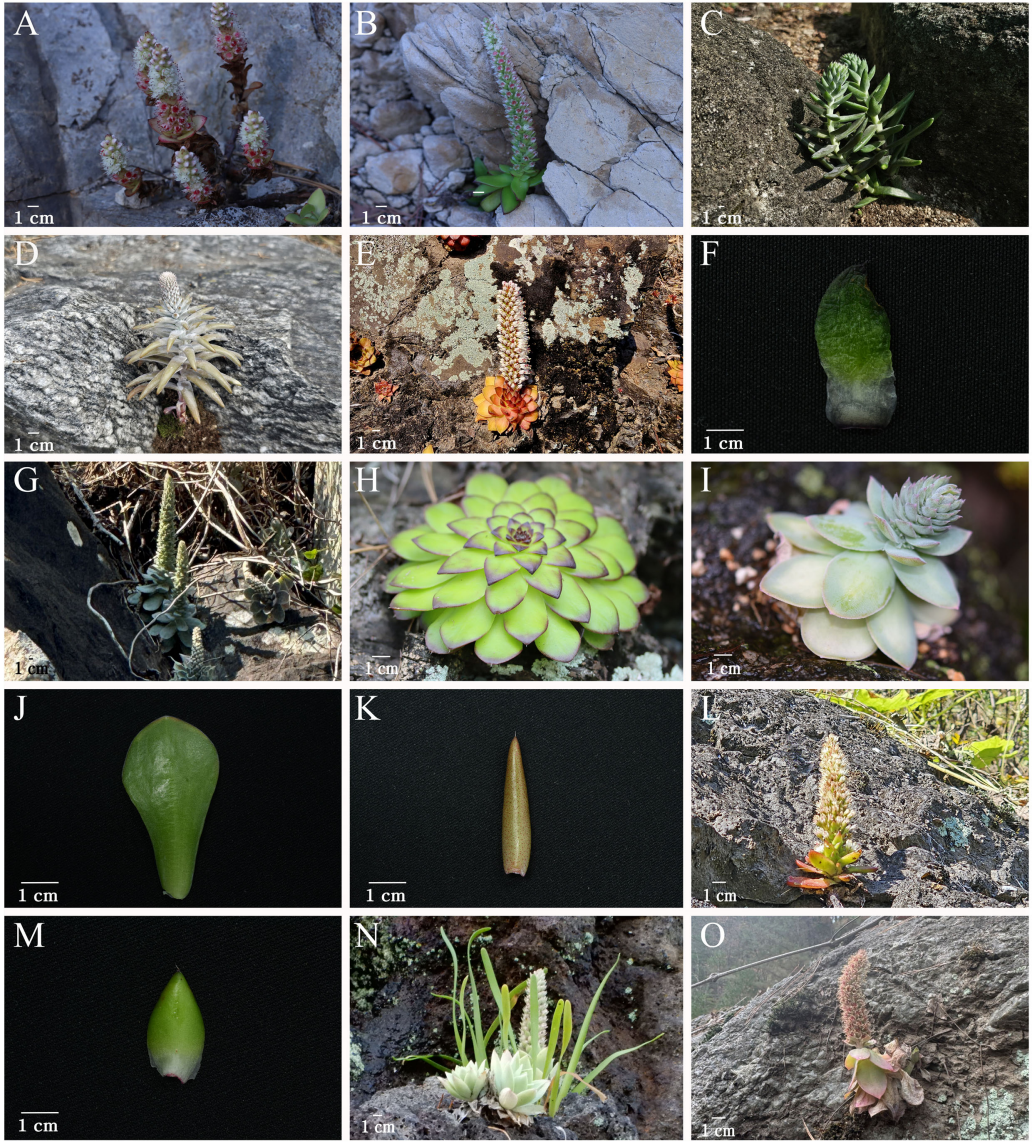
Photographs of *Orostachys* taxa used in this study. **(A)***Orostachys ramosa*, habit. **(B)***Orostachys malacophylla*, habit. **(C)***Orostachys japonica* f. *polycephala*, habit. **(D)***O. japonica*, habit. **(E)***Orostachys margaritifolia*, habit. **(F)***O. margaritifolia*, leaf. **(G)***Orostachys iwarenge*, habit. **(H)***O. margaritifolia*, rosette. **(I)***Orostachys chongsunensis*, habit. **(J)***O. chongsunensis*, leaf. **(K)***O. japonica*, leaf. **(L)***Orostachys latielliptica*, habit. **(M)***O. latielliptica*, leaf. **(N)***O. iwarenge* f. *magnus*, habit. **(O)** Unidentified *Orostachys* sp., habit.

With respect to individual taxa, *O. ramosa* (**Figure 2A**) was intermixed with *O. malacophylla* (**Figure 2B**), which is consistent with earlier work (Lee et al., 2022), confirming that distinguishing the two taxa as separate species is unreasonable. We fully agree with the conclusion by Lee et al. (2022) that “individuals with branching at the base of the stem represent individual variation”, and accordingly, we consider it appropriate to treat *O. ramosa* as a synonym of *O. malacophylla*, like *O. ramosa*, *O. japonica f. polycephala* (**Figure 2C**), like *O. ramosa* mentioned above, closely resembled *O. japonica* (**Figure 2D**) but was described as a new taxon characterized by branching at the base of the stem (Makino, 1910). This taxon also exhibited intermixed clustering with *O. japonica*, and since individuals with branching stems represent infraspecific variation within *O. japonica*, it would be reasonable to consider it a synonym of *O. japonica*.

*O. margaritifolia* (**Figure 2E**) was described as a new species by Lee and Lee (2000) and is distinguished from *O. malacophylla* (**Figure 2B**) by possessing thorn-like appendages at the leaf apex and ovate leaves (**Figure 2F**). Ohba (2003) treated this species as a synonym of *O. iwarenge* (**Figure 2G**). Consistent with previous studies (Lee et al., 2022), our analysis also revealed that it formed a completely independent lineage, strongly supporting its position as a species. Morphologically, it is clearly distinguished from all *Orostachys* subsect. *Orostachys* species, such as *O. iwarenge*, due to the appendages at the leaf apex and is distinguished from *Orostachys* subsect. *Appendiculatae* species by having rosette leaves that are slightly reflexed backward (**Figure 2H**) and ovate leaves (**Figure 2F**). Therefore, it is appropriate to treat *O. margaritifolia* as an independent species.

Ohba (2003) treated *O. chongsunensis* (**Figure 2I**), which was described as a new species based on its distinctive characteristics of possessing glaucous green leaves with purplish-red markings compared with *O. malacophylla* (**Figure 2B**), as a synonym of *O. japonica* (**Figure 2D**), while Lee et al. (2022) argued that it would be more appropriate to treat it as a synonym of *O. malacophylla* (**Figure 2B**). Morphologically, *O. chongsunensis* and *O. japonica* are clearly distinguished by the presence or absence of spine-like appendages at the leaf apex (**Figures 2J–K**). Additionally, our analysis revealed that *O. chongsunensis* is closely related to *O. iwarenge* and exhibited independent clustering, confirming a relatively distant relationship with *O. malacophylla*, which differs from Ohba’s (2003) treatment (**Figure 1**, **Supplementary Figures S3**-**S6**). Since the external morphology of *O. chongsunensis*, including its leaf characteristics, is more similar to that of *O. iwarenge* than to that of *O. malacophylla*, our results accurately reflect these morphological characteristics. However, our study includes only a single accession of *O. chongsunensis*, which limits definitive conclusions regarding species delimitation, and additional sampling with multiple individuals from different populations is needed to accurately confirm its taxonomic status.

*O. latielliptica* (**Figure 2L**) was described as a new species based on its characteristics of possessing glaucous, ovate leaves with thorn-like appendages at the leaf apex and one to four aggregated flowers per pedicel. This taxon was treated as a synonym of *O. japonica* by Ohba (2003), while Lee et al. (2022) recognized it as an independent species based on the morphological characteristics of the original description but noted that it showed a very close relationship with *O. japonica*, indicating the need for additional research. Our analysis revealed that *O. latielliptica* forms a sister group to the *O. japonica*–*O. margaritifolia* clade and is consistently retrieved as an independent lineage in all phylogenetic trees, supporting its recognition as an independent species (**Figure 1**, **Supplementary Figures S3**-**S6**). Based on numerous field observations, the characteristic of having multiple flowers per pedicel, as described by Lee and Lee (2000), was confirmed to be a non-reliable and inconsistent diagnostic character for this species. However, the ovate leaf morphology (**Figure 2M**) of *O. latielliptica* clearly distinguishes it from that of other taxa in the *Orostachys* subsect. *Appendiculatae*.

*O. iwarenge* f. *magnus* (**Figure 2N**) was treated as a synonym of *O. iwarenge* by Ohba (2003), but Lee et al. (2022) and Kim and Park (2005) recognized it as an independent taxon. Our analysis also revealed that this taxon is an independent lineage from *O. iwarenge*, strongly supporting the current taxonomy (**Figure 1**, **Supplementary Figures S3**-**S6**). Additionally, the clustering patterns shown in all the phylogenetic trees provide sufficient evidence to recognize *O. iwarenge* f. *magnus* as a species. An individual (*Orostachys* sp.; **Figure 2O**) morphologically very similar to *O. iwarenge* f. *magnus* was collected from an inland region of the Korean Peninsula (Samcheok-si, Gangwon-do), and a phylogenetic evaluation confirmed its closest relationship with *O. iwarenge* f. *magnus*. However, the clustering patterns between the concatenated ML tree and multi-locus species tree were somewhat different, suggesting that additional research using more individuals is needed to determine the exact relationship between these two taxa.

Moreover, *S. taquetii*, which was treated as a synonym of *Hylotelephium viridescens* (Nakai) H.Ohba without any taxonomic studies after its initial description, formed a single clade with *Hylotelephium erythrostictum* (Miq.) H.Ohba in all the phylogenetic trees of this study, indicating that it should be treated as a separate taxon from *H. viridescens*. Therefore, we support the opinion of Chung and Kim (1990) and An et al. (2023) regarding the taxonomic position of *S. taquetii* as an independent taxon within *Hylotelephium* rather than *Sedum*. These results indicate that taxonomic revision is needed to transfer this taxon to the genus *Hylotelephium* through a new nomenclatural combination.

## Conclusion

In this study, a phylogenomic analysis of the genus *Orostachys* was conducted via the Angiosperms353 target enrichment approach, and an in-depth consideration of longstanding taxonomic controversies within this morphologically complex group was provided. Our results confirmed that the genus *Orostachys* is polyphyletic, which is consistent with the findings of previous phylogenetic studies, with its two subsections (subsect. *Orostachys* and subsect. *Appendiculatae*) representing distinct evolutionary lineages. However, unlike in previous studies, we showed that *Orostachys* subsect. *Orostachys* forms an independent clade, which represents the most significant finding of this study. With respect to species delineation, we confirmed that *O. ramosa* and *O. japonica* f. *polycephala* should be treated as synonyms of *O. malacophylla* and *O. japonica*, respectively, while the endemic Korean species *O. margaritifolia*, *O. latielliptica*, *O. chongsunensis*, and *O. iwarenge* f. *magnus* should be recognized as independent taxa. The high concordance across all phylogenetic trees, regardless of dataset completeness thresholds or reconstruction methods, demonstrates the reliability of our results. Our findings confirm that the Angiosperms353 approach effectively resolves evolutionary relationships in taxonomically complex groups.

## Data Availability

The raw sequencing data have been deposited in the NCBI Sequencing Read Archive (SRA) under the BioProject ID PRJNA1310746. Individual SRA accession numbers for each taxon are provided in **Supplementary Table S1**. Assembled supercontig sequences are available on Zenodo (doi: 10.5281/zenodo.17645506).
