# Supplementary material for: Phylogenomic study of Orostachys using Angiosperms353: polyphyly of subsections with taxonomic consequences
Source: Front Plant Sci. 2025 Dec 12;16:1696546. doi: 10.3389/fpls.2025.1696546 (PMC12741063; doi:10.3389/fpls.2025.1696546)
Supplement: Supplementary file 1 [file DataSheet1.docx]

**Supplementary Materials**

**Method S1. Detailed Bioinformatics Pipeline and Analysis Parameters**

This document provides the complete, step-by-step pipeline, specific software versions, and command-line arguments used for all bioinformatics analyses conducted in this study, ensuring full reproducibility.

**- Data Processing and Preparation**

**Software:** Trimmomatic v0.38, HybPiper v1.3.1,MAFFT v7.490, trimAl v1.4, AMAS v1.0

| **Step** | **Command/Script** | **Parameters and Justification** |
| --- | --- | --- |
| Quality Control | trimmomatic PE -phred33 ILLUMINACLIP:TruSeq3-PE.fa:2:30:10 LEADING:3 TRAILING:3 SLIDINGWINDOW:4:15 MINLEN:36 | -phred33: Phred+33 quality score encoding  ILLUMINACLIP:TruSeq3-PE.fa:2:30:10: Remove Illumina adapters (seed mismatches: 2, palindrome threshold: 30, simple threshold: 10)  LEADING:3: Trim bases below quality 3 from read start  TRAILING:3: Trim bases below quality 3 from read end  SLIDINGWINDOW:4:15: Trim when 4-bp window average drops below 15  MINLEN:36: Discard reads shorter than 36 bp |
| Target Capture Assembly | hybpiper assemble -r -t_dna --prefix sample_name --cpu 8 --bwa | -r: Paired-end FASTQ input files  -t_dna: DNA target sequence file (reference genes)  --prefix: Sample name for output organization  --cpu 8: Use 8 threads for parallel processing  --bwa: BWA mapper for read alignment (alternative: BLAST/DIAMOND)  Internal pipeline: 1. BWA: Map reads to target genes. 2. SPAdes: De novo assembly of mapped reads (auto k-mer selection). 3. Exonerate (--model protein2genome): Extract gene regions using protein alignment. 4. Generate supercontigs (N-padding), identify introns (GFF annotation).  Output: Gene sequences (CDS, introns, supercontigs) with automatic paralog warnings |
| Paralog Detection and Filtering | hybpiper stats gene --targetfile_dna | gene: Generate gene-level statistics including paralog warnings  --targetfile_dna: Reference target file for comparison  Output files: seq_lengths.tsv: Sequence length matrix. genes_with_paralog_warnings.txt: List of paralog-flagged genes. Paralog criteria: Multiple contigs ≥75% of reference length (default threshold). Filtering strategy: Exclude paralog-warned genes from downstream phylogenetic analysis to retain putative single-copy orthologs. Note: Additional depth-based verification performed. |
| Sequence Retrieval | hybpiper retrieve_sequences dna --targetfile_dna --sample_names --fasta_dir | dna: Extract coding sequences (alternatives: aa, intron, supercontig)  --targetfile_dna: Reference target file  --sample_names: List of samples to process (excluding paralog-flagged genes)  --fasta_dir: Output directory for gene-wise FASTA files  Output: One FASTA file per gene containing sequences from all samples |
| Multiple Sequence Alignment | mafft --auto | --auto: Automatically select optimal alignment algorithm based on data size (FFT-NS-1, FFT-NS-2, or L-INS-i)  Additional filtering: Minimum sequence length 150 bp, minimum taxon occupancy 0.7 (genes present in ≥70% of samples) |
| Alignment Trimming | trimal -in -out -gt 0.8 -st 0.001 | -in: Input alignment file in FASTA format  -out: Output trimmed alignment file  -gt 0.8: Remove columns with >80% gaps  -st 0.001: Remove columns with similarity <0.001 (poorly conserved or ambiguously aligned positions)  Purpose: Improve phylogenetic signal by removing gap-rich and poorly aligned regions |
| Sequence Concatenation | amas concat -d dna -f fasta -p partitions.txt -o | concat: Concatenate multiple gene alignments into supermatrix  -i: Input alignment files (all trimmed gene alignments)  -d dna: Data type is DNA sequences  -f fasta: Input file format is FASTA  -p partitions.txt: Output partition file defining gene boundaries  -o concatenated.fasta: Output concatenated alignment file for phylogenetic analysis |

**- Phylogenetic Analysis**

**Software:** jModelTest v2.1.10, IQ-TREE v2.1.3, BioPyhon, ASTRAL-III v5.7.8

| **Step** | **Command/Script** | **Parameters and Justification** |
| --- | --- | --- |
| Model Selection | java -jar jModelTest.jar -d -g 4 -i -f -AIC -BIC -a | -d: Input alignment file  -g 4: Number of rate categories for gamma distribution  -i: Calculate proportion of invariable sites  -f: Include models with unequal base frequencies  -AIC: Akaike Information Criterion  -BIC: Bayesian Information Criterion (used for best model selection)  -a: Estimate model-averaged phylogeny  Output: Best-fit substitution model (e.g., GTR+G, HKY+I+G) |
| Maximum Likelihood Tree (IQ-TREE) | iqtree -s -m MODEL -bb 1000 -nt 8 | -s: Input alignment file  -m: Substitution model (from jModelTest, converted to IQ-TREE format)  -bb 1000: Ultrafast bootstrap with 1000 replicates  -nt 8: Use 8 CPU threads for parallel processing  Model conversion: GTR+G → GTR+G4, HKY+I+G → HKY+I+G4  Output: Maximum likelihood gene tree with bootstrap support |
| Tree Cleaning for ASTRAL | Python script using from Bio import Phylo | BioPython Phylo module used to: Remove bootstrap values from internal nodes (clade.confidence = None). Remove branch lengths from all nodes (clade.branch_length = None). Ensure proper Newick format (ending with semicolon). Remove extraneous quotes using regex.  Purpose: Prepare gene trees in format required by ASTRAL (topology only) |
| Species Tree Inference (ASTRAL) | java -jar astral.jar -i -o | -i: Input file containing all gene trees (one tree per line in Newick format)  -o: Output species tree file  Method: Coalescent-based species tree inference from gene trees  Handles incomplete lineage sorting (ILS)  Output: Species tree with local posterior probabilities as branch support  Analysis performed separately for: supercontig sequences. Individual gap thresholds (30%, 50%, 70%). Combined analysis across all thresholds. |

Table S1. Information for sample collection sites, voucher specimens, and GenBank accessions.

| **Sample** | **Source** | **Voucher** | **GenBank accession** | **Notes** |
| --- | --- | --- | --- | --- |
| *O. boehmeri* | Mount Apoi, Samani, Hidaka Subprefecture, Hokkaido, Japan | SJUH001460 | SRR35138008 |  |
| *O. chongsunensis* | Morun-ri, Jeongseon-gun, Gangwon-do Province, Korea | SJUH000864 | SRR35138002 | From type locality |
| *O. iwarenge*_1 | Wimihang, Namwon-eup, Seogwipo-si, Jeju Special Self-Governing Province, Korea | SJUH000981 | SRR35138039 |  |
| *O. iwarenge*_2 | Wimihang, Namwon-eup, Seogwipo-si, Jeju Special Self-Governing Province, Korea | SJUH000431 | SRR35138038 |  |
| *O. iwarenge* f. *magnus*_1 | Tongjumi beach, Namyang-ri, Ulleung-gun, Gyeongsangbuk-do Province, Korea | SJUH000982 | SRR35138041 |  |
| *O. iwarenge* f. *magnus*_2 | Sadong beach, Sadong-ri, Ulleung-gun, Gyeongsangbuk-do Province, Korea | SJUH000983 | SRR35138040 | From type locality |
| *O. japonica*_1 | Balsan-ri, Chuncheon-si, Gangwon-do Province, Korea | SJUH000988 | SRR35138032 |  |
| *O. japonica*_2 | Sambong beach, Changgi-ri, Taean-gun, Chungcheongnam-do Province, Korea | SJUH000985 | SRR35138029 |  |
| *O. japonica*_3 | Mt. Maneulbong, Geumgye-ri, Andong-si, Gyeongsangbuk-do Province, Korea | SJUH000986 | SRR35138033 |  |
| *O. japonica*_4 | Mt. Bojeoksan, Eup-ri, Wando-gun, Jeollanam-do Province, Korea | SJUH000987 | SRR35138030 |  |
| *O. japonica* f. *polycephala*_1 | Balsan-ri, Chuncheon-si, Gangwon-do Province, Korea | SJUH000992 | SRR35138036 |  |
| *O. japonica* f. *polycephala*_2 | Sambong beach, Changgi-ri, Taean-gun, Chungcheongnam-do Province, Korea | SJUH000989 | SRR35138034 |  |
| *O. japonica* f. *polycephala*_3 | Mt. Maneulbong, Geumgye-ri, Andong-si, Gyeongsangbuk-do Province, Korea | SJUH000990 | SRR35138037 |  |
| *O. japonica* f. *polycephala*_4 | Mt. Bojeoksan, Eup-ri, Wando-gun, Jeollanam-do Province, Korea | SJUH000991 | SRR35138035 |  |
| *O. latielliptica* | Hantan river, Oga-ri, Pocheon-si, Gyeonggi-do Province, Korea | SJUH000863 | SRR35138028 | From type locality |
| *O. margaritifolia* | Mt. Yangmasan, Panmun-dong, Jinju-si, Gyeongsangnam-do Province, Korea | SJUH000867 | SRR35138022 | From type locality |
| *O. malacophylla*_1 | Songjiho beach, Jngam-myeon, Goseong-gun, Gangwon-do Province, Korea | SJUH000971 | SRR35138024 |  |
| *O. malacophylla*_2 | Chuam beach, Chuam-dong, Donghae-si, Gangwon-do Province, Korea | SJUH000972 | SRR35138026 |  |
| *O. malacophylla*_3 | Oryugoara beach, Gampo-eup, Gyeongju-si, Gyeongsangnam-do Province, Korea | SJUH000973 | SRR35138025 |  |
| *O. malacophylla*_4 | Daewangam, Ilsan-dong, Dong-gu, Ulsan-si, Korea | SJUH000974 | SRR35138023 |  |
| *O. malacophylla*_5 | Dongbaek island, U-dong, Haeundae-gu, Busan-si, Korea | SJUH000975 | SRR35138027 |  |
| *O. minuta*_1 | Hantan river, Sangsa-ri, Cheorwon-gun, Gangwon-do Province, Korea | SJUH000994 | SRR35138019 |  |
| *O. minuta*_2 | Mt. Nochugol, Daegi-ri, Gangneung-si, Gangwon-do Province, Korea | SJUH000995 | SRR35138017 |  |
| *O. minuta*_3 | Mt. Cheongnyangsan, Bukgok-ri, Bonghwa-gun, Gyeongsangbuk-do Province, Korea | SJUH000435 | SRR35138021 |  |
| *O. minuta*_4 | Mt. Obongsan, Cheonchon-ri, Gyeongju-si, Gyeongsangbuk-do Province, Korea | SJUH000993 | SRR35138018 |  |
| *O. ramosa*_1 | Songjiho beach, Jngam-myeon, Goseong-gun, Gangwon-do Province, Korea | SJUH000976 | SRR35138014 |  |
| *O. ramosa*_2 | Chuam beach, Chuam-dong, Donghae-si, Gangwon-do Province, Korea | SJUH000977 | SRR35138016 |  |
| *O. ramosa*_3 | Oryugoara beach, Gampo-eup, Gyeongju-si, Gyeongsangnam-do Province, Korea | SJUH000978 | SRR35138015 |  |
| *O. ramosa*_4 | Daewangam, Ilsan-dong, Dong-gu, Ulsan-si, Korea | SJUH000979 | SRR35138013 |  |
| *O.* sp. | Punggok river, Punggok-ri, Samcheok-si, Gangwon-do Province, Korea | SJUH000984 | SRR35138001 |  |
| *O. spinosa* | Asralt Khairkhan Mountain, Nalaikh District, Ulaanbaatar, Mongolia | SJUH001461 | SRR35138010 |  |
| *O. thyrsiflora* | Bedel Pass, Naryn Region, Kyrgyzstan | SJUH001462 | SRR35138011 |  |
| *M. sikokianus* | Mt. Hwaaksan, Hwaak-ri, Gapyeong-gun, Gyeonggi-do Province, Korea | SJUH000434 | SRR35138003 |  |
| *H. erythrostictum* | Bonsin-ri, Yeongyang-gun, Gyeongsangbuk-do Province, Korea | SJUH000869 | SRR35138043 |  |
| *H. pallescens* | Mt. Taegisan, Taegi-ri, Heongseong-gun, Gangwon-do Province, Korea | SJUH000871 | SRR35138042 |  |
| *H. spectabile* | Sanseong-ri, Gwangju-si, Gyeonggi-do Province, Korea | SJUH000870 | SRR35138031 |  |
| *H. ussuriense* | Mt. Phalkaksan, Jueung-ri, Yeongdeok-gun, Gyeongsangbuk-do Province, Korea | SJUH000874 | SRR35138020 |  |
| *H. verticillatum* | Mt. Hwaaksan, Hwaak-ri, Gapyeong-gun, Gyeonggi-do Province, Korea | SJUH000996 | SRR35138009 |  |
| *H. viridescens* | Haean-dong, Jeju-si, Jeju Special Self-Governing Province, Korea | SJUH000872 | SRR35138005 |  |
| *H. viviparum* | Mt. Hwaaksan, Hwaak-ri, Gapyeong-gun, Gyeonggi-do Province, Korea | SJUH000997 | SRR35138004 |  |
| *S. taquetii* | Haean-dong, Jeju-si, Jeju Special Self-Governing Province, Korea | SJUH000873 | SRR35138012 |  |
| *P. aizoon* | Geumgok-dong, Buk-gu, Gwangj-si, Province, Korea | SJUH000998 | SRR35138006 |  |
| *P. aizoon* var. *floribundus* | Gangchon-ri, Chuncheon-si, Gangwon-do Province, Korea | SJUH000999 | SRR35138007 |  |

Table S2. Summary of sequencing platforms employed for sample analysis.

| **Platform** | **Manufacturer** | **Taxa** | **Read length** |
| --- | --- | --- | --- |
| MGI-400 | MGI-Tech Co. (Shenzhen, China) | *O. chongsunensis* | 150 bp |
|  |  | *O. iwarenge_1* |  |
|  |  | *O. iwarenge* f. *magnus_2* |  |
|  |  | *O. japonica_3* |  |
|  |  | *O. japonica* f. *polycephala_3* |  |
|  |  | *O. malacophylla_5* |  |
|  |  | *O. margaritifolia* |  |
|  |  | *O. minuta_1* |  |
|  |  | *O. ramosa_1* |  |
|  |  | *Meterostachys sikokianus* |  |
|  |  | *H. erythrostictum* |  |
|  |  | *H. verticillatum* |  |
|  |  | *H. viridescens* |  |
|  |  | *S. taquetii* |  |
| Novaseq 6000 | Illumina Inc. (San Diego, CA, USA) |  | 100 bp |
|  |  | *O. iwarenge_2* |  |
|  |  | *O. iwarenge* f. *magnus_1* |  |
|  |  | *O. japonica_1* |  |
|  |  | *O. japonica_2* |  |
|  |  | *O. japonica_4* |  |
|  |  | *O. japonica* f. *polycephala_1* |  |
|  |  | *O. japonica* f. *polycephala_2* |  |
|  |  | *O. japonica* f. *polycephala_4* |  |
|  |  | *O. latielliptica* |  |
|  |  | *O. malacophylla_1* |  |
|  |  | *O. malacophylla_2* |  |
|  |  | *O. malacophylla_3* |  |
|  |  | *O. malacophylla_4* |  |
|  |  | *O. minuta_2* |  |
|  |  | *O. minuta_3* |  |
|  |  | *O. minuta_4* |  |
|  |  | *O. ramosa_2* |  |
|  |  | *O. ramosa_3* |  |
|  |  | *O. ramosa_4* |  |
|  |  | *O.* sp. |  |
|  |  | *H. pallescens* |  |
|  |  | *H. spectabile* |  |
|  |  | *H. ussuriense* |  |
|  |  | *H. viviparum* |  |
| Novaseq X | Illumina Inc. (San Diego, CA, USA) | *O. boehmeri* | 150 bp |
|  |  | *O. spinosa* |  |
|  |  | *O. thyrsiflora* |  |
|  |  | *P. aizoon* |  |
|  |  | *P. aizoon* var. *floribundus* |  |

Table S3. Paralog detection statistics for all samples and loci.

| **Sample** | **Gene** | **No. Long Contigs** | **Max_Coverage** | **Min_Coverage** | **Max_Length** | **Min_Length** |
| --- | --- | --- | --- | --- | --- | --- |
| *O. boehmeri* | 4951 | 2 | 289.6 | 245.7 | 459 | 456 |
|  | 5460 | 2 | 234.7 | 75.4 | 348 | 348 |
|  | 5840 | 2 | 1048.1 | 414.8 | 825 | 699 |
|  | 5859 | 2 | 581 | 574.8 | 630 | 627 |
|  | 5910 | 2 | 358 | 302.1 | 717 | 717 |
|  | 5942 | 2 | 182.1 | 121.4 | 531 | 468 |
|  | 5958 | 2 | 174.6 | 108.3 | 333 | 315 |
|  | 6003 | 2 | 245.8 | 143.8 | 219 | 219 |
|  | 6068 | 2 | 330.3 | 312.1 | 252 | 249 |
|  | 6498 | 3 | 668.6 | 548.9 | 285 | 279 |
|  | 6506 | 2 | 767.8 | 206.4 | 438 | 408 |
|  | 6780 | 2 | 234.1 | 90 | 321 | 318 |
|  | 6875 | 2 | 74.2 | 33.9 | 360 | 321 |
| *O. chongsunensis* | 4932 | 2 | 340.8 | 32.1 | 969 | 897 |
|  | 5206 | 2 | 80 | 9.4 | 459 | 459 |
|  | 5347 | 2 | 71.3 | 50.4 | 210 | 207 |
|  | 5348 | 2 | 11.5 | 6 | 474 | 465 |
|  | 5460 | 2 | 53.1 | 33.2 | 396 | 390 |
|  | 5840 | 2 | 781.3 | 507.6 | 825 | 660 |
|  | 5859 | 2 | 29.6 | 22 | 744 | 630 |
|  | 5910 | 2 | 270.6 | 161.9 | 717 | 717 |
|  | 5919 | 2 | 73.2 | 12.6 | 426 | 378 |
|  | 5942 | 2 | 173.5 | 79.4 | 552 | 531 |
|  | 5980 | 2 | 32.9 | 18.8 | 498 | 486 |
|  | 6003 | 2 | 150.1 | 101.9 | 219 | 219 |
|  | 6068 | 2 | 43 | 34.4 | 252 | 249 |
|  | 6114 | 2 | 126.2 | 106.6 | 558 | 558 |
|  | 6274 | 2 | 20.9 | 14.5 | 516 | 483 |
|  | 6496 | 2 | 117.8 | 99.2 | 588 | 537 |
|  | 6498 | 2 | 66.3 | 48.2 | 282 | 282 |
|  | 6875 | 2 | 77.4 | 14 | 387 | 387 |
| *O. iwarenge*_1 | 4932 | 2 | 196.7 | 71.3 | 975 | 897 |
|  | 4951 | 2 | 7.7 | 6.5 | 465 | 462 |
|  | 5188 | 2 | 444.4 | 49.5 | 552 | 447 |
|  | 5206 | 2 | 45.7 | 5.2 | 459 | 459 |
|  | 5318 | 2 | 99.9 | 52.6 | 600 | 462 |
|  | 5460 | 2 | 37.3 | 16.8 | 396 | 390 |
|  | 5840 | 2 | 468.3 | 378.8 | 825 | 660 |
|  | 5859 | 2 | 22.2 | 18.6 | 792 | 630 |
|  | 5910 | 2 | 146 | 144.9 | 717 | 717 |
|  | 5942 | 2 | 104.8 | 69 | 552 | 501 |
|  | 5945 | 2 | 39.1 | 30.3 | 780 | 777 |
|  | 6003 | 2 | 84.8 | 70.8 | 219 | 219 |
|  | 6068 | 2 | 33.4 | 30.4 | 225 | 225 |
|  | 6274 | 2 | 17 | 16.9 | 549 | 483 |
|  | 6295 | 2 | 585.7 | 406.5 | 1158 | 1158 |
|  | 6498 | 2 | 79.8 | 15.1 | 282 | 282 |
|  | 6506 | 2 | 117.9 | 27.5 | 504 | 396 |
|  | 6746 | 2 | 71.9 | 46.2 | 321 | 288 |
|  | 6780 | 2 | 58.7 | 44 | 318 | 306 |
|  | 6909 | 2 | 81.4 | 26.3 | 525 | 507 |
| *O. iwarenge*_2 | 5188 | 2 | 2335 | 93.5 | 540 | 471 |
|  | 5460 | 2 | 1326.5 | 621.1 | 396 | 390 |
|  | 5858 | 2 | 6288.6 | 4952.6 | 504 | 504 |
|  | 5910 | 2 | 1728.3 | 1647.1 | 702 | 699 |
|  | 5942 | 2 | 1378.8 | 685.8 | 567 | 498 |
|  | 5958 | 2 | 449.1 | 375.3 | 333 | 267 |
|  | 6003 | 2 | 1104.7 | 771.4 | 219 | 219 |
|  | 6068 | 2 | 2085.2 | 1468.6 | 252 | 249 |
|  | 6498 | 2 | 6233 | 3121.4 | 282 | 282 |
| *O. iwarenge* f. *magnus*_1 | 4951 | 2 | 703 | 540.1 | 459 | 459 |
|  | 5188 | 2 | 2196.9 | 98.3 | 552 | 471 |
|  | 5318 | 2 | 2513.2 | 1763.1 | 462 | 459 |
|  | 5347 | 2 | 644.8 | 327.5 | 210 | 210 |
|  | 5460 | 2 | 865.2 | 312.6 | 390 | 312 |
|  | 5840 | 2 | 3262.1 | 2631.9 | 825 | 660 |
|  | 5858 | 2 | 3445.6 | 2823.7 | 573 | 504 |
|  | 5859 | 2 | 2072.9 | 1231.6 | 747 | 630 |
|  | 5919 | 2 | 901.8 | 714.3 | 444 | 354 |
|  | 5942 | 2 | 542.5 | 338.9 | 552 | 477 |
|  | 5958 | 2 | 344.5 | 300.4 | 333 | 303 |
|  | 6068 | 2 | 1235.1 | 1092.2 | 252 | 249 |
|  | 6114 | 2 | 683.8 | 585.3 | 558 | 558 |
|  | 6295 | 2 | 5524.3 | 4900.8 | 1158 | 1152 |
|  | 6498 | 2 | 5289.5 | 3382.6 | 282 | 282 |
|  | 6854 | 2 | 790.5 | 176.4 | 639 | 633 |
|  | 6875 | 2 | 255.1 | 225 | 387 | 387 |
| *O. iwarenge* f. *magnus*_2 | 4951 | 2 | 22.3 | 10.7 | 462 | 444 |
|  | 5188 | 2 | 673.6 | 93.6 | 471 | 447 |
|  | 5460 | 2 | 47.7 | 14.1 | 393 | 390 |
|  | 5840 | 2 | 654.9 | 471.6 | 825 | 660 |
|  | 5858 | 2 | 167.3 | 65.7 | 597 | 504 |
|  | 5859 | 2 | 38.1 | 15.4 | 783 | 627 |
|  | 5919 | 2 | 32.8 | 5.8 | 444 | 387 |
|  | 5942 | 2 | 137.5 | 33.6 | 552 | 522 |
|  | 5958 | 2 | 34.2 | 6.3 | 333 | 333 |
|  | 6003 | 2 | 127.3 | 109.9 | 219 | 219 |
|  | 6068 | 2 | 53.1 | 43.6 | 225 | 225 |
|  | 6114 | 2 | 124.4 | 84.6 | 558 | 558 |
|  | 6496 | 2 | 198.9 | 31.6 | 690 | 588 |
|  | 6498 | 2 | 46 | 40 | 282 | 282 |
|  | 6506 | 2 | 261.4 | 71.8 | 504 | 438 |
|  | 6854 | 2 | 45.1 | 27.1 | 645 | 606 |
|  | 6875 | 2 | 65.1 | 9.2 | 387 | 381 |
| *O. japonica*_1 | 4951 | 2 | 1263.5 | 1024.3 | 459 | 459 |
|  | 5328 | 2 | 1523.8 | 327.9 | 438 | 438 |
|  | 5940 | 2 | 2065 | 1442 | 819 | 816 |
|  | 5949 | 2 | 1809.2 | 1260.5 | 462 | 438 |
|  | 6068 | 2 | 1459.6 | 1064.5 | 225 | 225 |
|  | 6498 | 2 | 5560.5 | 2401.3 | 285 | 285 |
| *O. japonica*_2 | 4527 | 2 | 2967.9 | 865.2 | 1161 | 1014 |
|  | 4951 | 2 | 2356.8 | 870.2 | 576 | 576 |
|  | 5460 | 2 | 412.7 | 259.5 | 381 | 327 |
|  | 5940 | 2 | 2340.9 | 1705.1 | 819 | 651 |
|  | 5949 | 2 | 1923.5 | 1729.9 | 462 | 438 |
|  | 5950 | 2 | 1272.9 | 1081.9 | 648 | 522 |
|  | 6068 | 2 | 1160.6 | 623.1 | 231 | 225 |
|  | 6114 | 2 | 756.2 | 241.6 | 573 | 570 |
|  | 6498 | 2 | 4114.2 | 3383.3 | 279 | 279 |
|  | 6854 | 2 | 555.8 | 388.5 | 690 | 672 |
|  | 6995 | 2 | 12.8 | 8.5 | 204 | 204 |
| *O. japonica*_3 | 4951 | 2 | 165.8 | 25.7 | 555 | 465 |
|  | 5328 | 2 | 294.8 | 255.8 | 438 | 438 |
|  | 5460 | 2 | 114.8 | 91 | 396 | 384 |
|  | 5821 | 3 | 329.1 | 44.6 | 477 | 372 |
|  | 5940 | 2 | 432.6 | 323.7 | 825 | 786 |
|  | 5945 | 2 | 558.7 | 65 | 717 | 717 |
|  | 5950 | 3 | 777.8 | 623 | 561 | 543 |
|  | 6068 | 2 | 150.4 | 48.5 | 231 | 225 |
|  | 6114 | 2 | 377.4 | 121.8 | 561 | 552 |
|  | 6387 | 2 | 330.6 | 78.9 | 786 | 786 |
|  | 6498 | 2 | 171.4 | 152.7 | 285 | 285 |
|  | 6854 | 2 | 251 | 36 | 693 | 678 |
| *O. japonica*_4 | 4951 | 2 | 1714.4 | 1444.1 | 570 | 459 |
|  | 5328 | 2 | 1359.8 | 449 | 438 | 438 |
|  | 5634 | 2 | 2230.8 | 1261.1 | 825 | 744 |
|  | 5940 | 2 | 2118.3 | 943.9 | 819 | 816 |
|  | 5950 | 3 | 1295.9 | 836.1 | 543 | 534 |
|  | 6068 | 2 | 1052.7 | 911.4 | 225 | 225 |
|  | 6114 | 2 | 1241.5 | 239.2 | 531 | 459 |
|  | 6498 | 2 | 3136.9 | 2457.8 | 282 | 282 |
|  | 6995 | 2 | 26.6 | 6.3 | 204 | 204 |
| *O. japonica* f. *polycephala*_1 | 5328 | 2 | 1850.8 | 924.3 | 438 | 438 |
|  | 5347 | 2 | 464.6 | 265 | 210 | 201 |
|  | 5460 | 2 | 533.9 | 532.4 | 348 | 312 |
|  | 5940 | 2 | 2384 | 1762.8 | 819 | 816 |
|  | 5949 | 2 | 1961.6 | 1370.7 | 462 | 438 |
|  | 6068 | 2 | 1273.1 | 1106.3 | 231 | 225 |
|  | 6496 | 2 | 1225.5 | 768.1 | 540 | 537 |
|  | 6498 | 2 | 4079.2 | 2965.9 | 279 | 279 |
| *O. japonica* f. *polycephala*_2 | 4951 | 2 | 4020.9 | 1118.9 | 465 | 465 |
|  | 6068 | 2 | 1248.2 | 720.7 | 231 | 225 |
|  | 6496 | 2 | 1348.7 | 722.5 | 588 | 540 |
|  | 6995 | 2 | 23.6 | 11 | 204 | 183 |
| *O. japonica* f. *polycephala*_3 | 4527 | 2 | 490.3 | 106.5 | 1101 | 1035 |
|  | 4951 | 2 | 249.2 | 25.5 | 570 | 465 |
|  | 5328 | 2 | 393.4 | 158.9 | 438 | 396 |
|  | 5460 | 2 | 90.9 | 19.5 | 396 | 393 |
|  | 5821 | 2 | 397.5 | 356.9 | 405 | 372 |
|  | 5940 | 2 | 763.5 | 150.3 | 825 | 825 |
|  | 5949 | 2 | 459.8 | 159 | 477 | 456 |
|  | 6068 | 2 | 136.1 | 56.8 | 231 | 225 |
|  | 6114 | 2 | 215.8 | 119 | 561 | 552 |
|  | 6295 | 2 | 1940.5 | 1429.5 | 1158 | 951 |
|  | 6387 | 2 | 338.4 | 128.9 | 786 | 786 |
|  | 6496 | 2 | 418.5 | 316.7 | 588 | 585 |
|  | 6498 | 2 | 171.1 | 170.3 | 285 | 285 |
| *O. japonica* f. *polycephala*_4 | 5328 | 2 | 1067.8 | 341.8 | 438 | 438 |
|  | 5634 | 2 | 1890 | 1068.9 | 825 | 744 |
|  | 5940 | 2 | 1464.2 | 744.2 | 819 | 816 |
|  | 5950 | 2 | 999.1 | 703.7 | 543 | 534 |
|  | 6068 | 2 | 783.7 | 726.6 | 225 | 225 |
|  | 6114 | 2 | 981.7 | 188.8 | 531 | 459 |
|  | 6496 | 2 | 637 | 570.8 | 690 | 585 |
|  | 6498 | 2 | 2618.6 | 2056.9 | 279 | 279 |
| *O. latielliptica* | 4951 | 2 | 1200.7 | 553.6 | 549 | 465 |
|  | 5460 | 2 | 1071.8 | 552.6 | 396 | 330 |
|  | 5940 | 2 | 1791 | 1114.6 | 819 | 807 |
|  | 6139 | 2 | 2751.3 | 2686.9 | 909 | 903 |
|  | 6387 | 2 | 2814.2 | 2079.3 | 786 | 783 |
|  | 6496 | 2 | 1216.4 | 717.8 | 588 | 585 |
|  | 6498 | 2 | 3449.9 | 3146.6 | 279 | 279 |
|  | 6854 | 3 | 413.1 | 357 | 717 | 612 |
|  | 7363 | 2 | 1380.7 | 1107.3 | 480 | 471 |
| *O. margaritifolia* | 4951 | 3 | 100 | 8.9 | 576 | 465 |
|  | 5188 | 2 | 218.1 | 5.5 | 471 | 471 |
|  | 5949 | 2 | 391.4 | 178.8 | 453 | 432 |
|  | 5950 | 3 | 749.9 | 414.5 | 561 | 534 |
|  | 6068 | 2 | 115.2 | 115 | 225 | 225 |
|  | 6114 | 2 | 303.1 | 87.2 | 561 | 552 |
|  | 6387 | 2 | 278.6 | 96.5 | 780 | 759 |
|  | 6496 | 2 | 559.3 | 181.9 | 690 | 585 |
|  | 6498 | 2 | 122.9 | 120.8 | 285 | 285 |
|  | 6780 | 2 | 18.5 | 17.7 | 312 | 312 |
|  | 6854 | 2 | 95.1 | 23.7 | 678 | 666 |
| *O. malacophylla*_1 | 5840 | 2 | 397.7 | 359.7 | 825 | 660 |
|  | 5919 | 2 | 206.8 | 84.8 | 381 | 354 |
|  | 5942 | 2 | 108.2 | 74.9 | 567 | 489 |
|  | 6068 | 2 | 156.1 | 97.7 | 252 | 249 |
|  | 6295 | 2 | 778 | 656.7 | 1158 | 1158 |
|  | 6498 | 2 | 554.4 | 451.3 | 282 | 282 |
|  | 6506 | 2 | 201.8 | 47 | 405 | 390 |
|  | 6780 | 2 | 113.7 | 51.1 | 318 | 318 |
| *O. malacophylla*_2 | 4951 | 2 | 44.8 | 38.6 | 462 | 462 |
|  | 5460 | 2 | 112.8 | 48.7 | 396 | 390 |
|  | 5840 | 2 | 358.8 | 313.4 | 825 | 660 |
|  | 5910 | 2 | 167.4 | 160 | 702 | 699 |
|  | 5919 | 2 | 164.5 | 84.1 | 444 | 354 |
|  | 5942 | 2 | 103 | 57.9 | 567 | 498 |
|  | 6003 | 2 | 132.1 | 100.8 | 219 | 219 |
|  | 6068 | 2 | 132.1 | 114.5 | 240 | 240 |
|  | 6295 | 2 | 622.1 | 592.6 | 1158 | 1158 |
|  | 6498 | 2 | 455.8 | 385.3 | 282 | 282 |
|  | 6780 | 2 | 88.1 | 86.7 | 318 | 318 |
|  | 6875 | 2 | 32 | 15.5 | 387 | 387 |
| *O. malacophylla*_3 | 4951 | 3 | 1181.6 | 186.3 | 459 | 456 |
|  | 5460 | 2 | 564.6 | 215.1 | 396 | 390 |
|  | 5859 | 2 | 2601.9 | 1041.5 | 744 | 630 |
|  | 5919 | 2 | 893.9 | 341.4 | 435 | 342 |
|  | 5942 | 2 | 443 | 393.7 | 567 | 483 |
|  | 5958 | 2 | 208.4 | 195.5 | 303 | 267 |
|  | 6003 | 2 | 658.7 | 458.9 | 219 | 219 |
|  | 6068 | 2 | 918.7 | 878.8 | 252 | 249 |
|  | 6295 | 2 | 4089.7 | 3814.6 | 1158 | 1158 |
|  | 6498 | 2 | 5205 | 2727 | 282 | 282 |
|  | 6875 | 2 | 135.4 | 110.9 | 387 | 387 |
| *O. malacophylla*_4 | 5859 | 2 | 289.7 | 250.2 | 630 | 627 |
|  | 5919 | 2 | 189.5 | 85.4 | 378 | 354 |
|  | 5958 | 2 | 35.7 | 34.8 | 300 | 264 |
|  | 6068 | 2 | 168.5 | 149.6 | 252 | 249 |
|  | 6295 | 2 | 499.2 | 419.6 | 1158 | 1158 |
|  | 6498 | 2 | 521.2 | 413.7 | 282 | 282 |
| *O. malacophylla*_5 | 4951 | 3 | 32.1 | 4.7 | 465 | 447 |
|  | 5206 | 2 | 73 | 12 | 459 | 459 |
|  | 5348 | 2 | 16.9 | 10.2 | 492 | 444 |
|  | 5460 | 2 | 90.8 | 38.9 | 396 | 390 |
|  | 5551 | 2 | 41.3 | 30.3 | 855 | 756 |
|  | 5859 | 2 | 37.2 | 34.9 | 804 | 630 |
|  | 5910 | 2 | 274.5 | 207.3 | 717 | 717 |
|  | 5942 | 2 | 171.5 | 130.1 | 552 | 552 |
|  | 5945 | 2 | 197.8 | 66.7 | 780 | 777 |
|  | 5958 | 2 | 37.9 | 4.7 | 333 | 309 |
|  | 6068 | 2 | 72.8 | 62.8 | 225 | 225 |
|  | 6114 | 2 | 123.1 | 120.5 | 567 | 456 |
|  | 6274 | 2 | 39.8 | 33.6 | 573 | 537 |
|  | 6498 | 2 | 98.7 | 70.7 | 282 | 282 |
|  | 6746 | 2 | 189.5 | 166 | 321 | 288 |
|  | 6780 | 2 | 225.6 | 136.6 | 318 | 318 |
|  | 6875 | 2 | 69.3 | 16.6 | 387 | 387 |
|  | 6909 | 2 | 132.7 | 64.4 | 525 | 507 |
| *O. minuta*_1 | 4951 | 2 | 74.9 | 24.1 | 576 | 465 |
|  | 5460 | 2 | 212.7 | 5.9 | 366 | 348 |
|  | 5802 | 2 | 192.5 | 76.8 | 243 | 243 |
|  | 5821 | 3 | 454.7 | 64.8 | 402 | 372 |
|  | 5949 | 2 | 624.1 | 389.4 | 453 | 426 |
|  | 5950 | 3 | 976.8 | 237.1 | 657 | 546 |
|  | 5974 | 2 | 1407.7 | 145.9 | 567 | 555 |
|  | 6068 | 2 | 127.9 | 48.7 | 225 | 225 |
|  | 6114 | 2 | 399.5 | 119.9 | 561 | 552 |
|  | 6387 | 2 | 614.1 | 100.3 | 780 | 768 |
|  | 6492 | 3 | 654.6 | 314.4 | 492 | 417 |
|  | 6498 | 2 | 248.8 | 195.9 | 285 | 285 |
|  | 6854 | 2 | 166.4 | 42.1 | 690 | 678 |
| *O. minuta*_2 | 4951 | 2 | 1102.9 | 896.9 | 570 | 513 |
|  | 5821 | 2 | 3236.6 | 1003.6 | 402 | 372 |
|  | 5940 | 2 | 2205.3 | 1295.2 | 819 | 819 |
|  | 5974 | 2 | 1072.1 | 533.9 | 567 | 555 |
|  | 6068 | 2 | 1051.6 | 732.2 | 225 | 225 |
|  | 6114 | 2 | 1583.4 | 218.5 | 573 | 492 |
|  | 6387 | 2 | 3624.7 | 2997.1 | 786 | 786 |
|  | 6498 | 2 | 7776.3 | 3846.5 | 279 | 279 |
|  | 6854 | 2 | 367.2 | 347.6 | 693 | 669 |
| *O. minuta*_3 | 4951 | 2 | 1186.7 | 535.9 | 570 | 534 |
|  | 5942 | 2 | 505.5 | 409.9 | 552 | 450 |
|  | 5949 | 2 | 1876.4 | 1646.7 | 462 | 438 |
|  | 5950 | 2 | 1437.1 | 845.7 | 540 | 534 |
|  | 5974 | 2 | 2817.6 | 504.3 | 567 | 555 |
|  | 6068 | 2 | 1047.5 | 864.1 | 225 | 225 |
|  | 6114 | 2 | 1093.8 | 215.5 | 573 | 573 |
|  | 6387 | 2 | 3710.2 | 3407 | 786 | 759 |
|  | 6496 | 2 | 1151.2 | 683 | 690 | 540 |
|  | 6498 | 2 | 3654.1 | 3573.3 | 279 | 279 |
|  | 7572 | 2 | 6436.2 | 527.3 | 741 | 678 |
| *O. minuta*_4 | 4951 | 2 | 1240.3 | 1117.7 | 570 | 459 |
|  | 6068 | 2 | 1138.1 | 533.2 | 225 | 225 |
|  | 6387 | 2 | 3646.7 | 3426.1 | 786 | 672 |
|  | 6492 | 2 | 2502.7 | 1802.2 | 492 | 492 |
|  | 6498 | 2 | 4283.5 | 4046.3 | 282 | 282 |
| *O. ramosa*_1 | 4951 | 3 | 55.4 | 12.9 | 465 | 441 |
|  | 5206 | 2 | 125.8 | 13.8 | 459 | 459 |
|  | 5347 | 2 | 129.2 | 77.8 | 210 | 207 |
|  | 5460 | 2 | 70.1 | 35.7 | 396 | 390 |
|  | 5551 | 2 | 61.7 | 31.2 | 777 | 765 |
|  | 5858 | 2 | 260.7 | 149.9 | 573 | 504 |
|  | 5859 | 2 | 45 | 42.2 | 774 | 630 |
|  | 5942 | 2 | 171.2 | 166.8 | 552 | 552 |
|  | 5945 | 2 | 191.7 | 54.4 | 780 | 777 |
|  | 6068 | 2 | 65 | 44.2 | 225 | 225 |
|  | 6114 | 2 | 239.6 | 115.6 | 558 | 447 |
|  | 6130 | 2 | 162.8 | 76.2 | 453 | 357 |
|  | 6496 | 2 | 315 | 61.6 | 588 | 537 |
|  | 6498 | 2 | 71.9 | 65.3 | 282 | 282 |
|  | 6506 | 2 | 218.8 | 90.9 | 504 | 429 |
|  | 6780 | 2 | 259.6 | 70.6 | 318 | 318 |
|  | 6875 | 2 | 118.4 | 8.6 | 387 | 387 |
|  | 6909 | 2 | 186.5 | 68.1 | 525 | 507 |
|  | 7067 | 2 | 181.1 | 10.8 | 351 | 333 |
| *O. ramosa*_2 | 4951 | 4 | 1435.3 | 283.6 | 459 | 456 |
|  | 5840 | 2 | 3136.4 | 2411.4 | 825 | 660 |
|  | 5919 | 2 | 976.6 | 709 | 450 | 354 |
|  | 5942 | 2 | 861.2 | 540.6 | 567 | 498 |
|  | 5945 | 2 | 1198.4 | 1121.7 | 780 | 588 |
|  | 5958 | 2 | 377.8 | 279.3 | 333 | 273 |
|  | 6068 | 2 | 1155.8 | 1053.5 | 225 | 225 |
|  | 6498 | 2 | 5977.1 | 3043.8 | 282 | 282 |
|  | 6746 | 2 | 506.3 | 476.6 | 321 | 288 |
|  | 6875 | 2 | 239 | 179.7 | 387 | 387 |
|  | 6909 | 2 | 1326.6 | 883.2 | 525 | 483 |
|  | 6992 | 2 | 1999.6 | 11.3 | 384 | 378 |
| *O. ramosa*_3 | 4951 | 4 | 1100.4 | 243.9 | 459 | 453 |
|  | 5188 | 2 | 2073.9 | 136.5 | 552 | 471 |
|  | 5460 | 2 | 849.8 | 420.6 | 396 | 390 |
|  | 5858 | 2 | 3523.7 | 2978.4 | 573 | 504 |
|  | 5859 | 2 | 2350.1 | 1378.6 | 741 | 630 |
|  | 5910 | 2 | 1279.2 | 1232.8 | 717 | 717 |
|  | 5919 | 2 | 1087.2 | 587.6 | 393 | 354 |
|  | 5942 | 2 | 763.9 | 534.9 | 567 | 498 |
|  | 5958 | 2 | 368.6 | 303.1 | 333 | 267 |
|  | 6068 | 2 | 1018.1 | 953.3 | 252 | 249 |
|  | 6114 | 2 | 519.7 | 435 | 558 | 558 |
|  | 6498 | 2 | 3710.2 | 3386.3 | 282 | 282 |
|  | 6780 | 2 | 632.9 | 464.1 | 318 | 318 |
|  | 6875 | 2 | 321.9 | 263.3 | 387 | 387 |
| *O. ramosa*_4 | 4951 | 2 | 277.3 | 163.2 | 456 | 423 |
|  | 5188 | 2 | 1192.3 | 101.5 | 534 | 471 |
|  | 5348 | 2 | 893.2 | 65.8 | 459 | 417 |
|  | 5460 | 2 | 577 | 230.1 | 396 | 390 |
|  | 5910 | 2 | 705.8 | 620.7 | 702 | 699 |
|  | 5919 | 2 | 935 | 429 | 387 | 354 |
|  | 5942 | 2 | 428.4 | 307.6 | 567 | 498 |
|  | 5958 | 2 | 291.5 | 177.1 | 303 | 267 |
|  | 6003 | 2 | 406.2 | 262.2 | 219 | 219 |
|  | 6068 | 2 | 569.6 | 536.5 | 240 | 240 |
|  | 6114 | 2 | 366.8 | 254.5 | 558 | 558 |
|  | 6498 | 2 | 2620.6 | 1745.7 | 282 | 282 |
|  | 6780 | 2 | 370.4 | 200.3 | 318 | 318 |
|  | 6875 | 2 | 174.8 | 149.4 | 387 | 387 |
| *O*. sp. | 5188 | 2 | 2325 | 91.6 | 552 | 471 |
|  | 5460 | 2 | 929.8 | 287.7 | 396 | 390 |
|  | 5859 | 2 | 1747.2 | 1065.7 | 630 | 627 |
|  | 5919 | 2 | 1337.4 | 603.8 | 435 | 354 |
|  | 5942 | 2 | 567.5 | 311.1 | 552 | 477 |
|  | 5958 | 2 | 473.3 | 223.5 | 333 | 303 |
|  | 6875 | 2 | 235 | 230.9 | 387 | 387 |
| *O. spinosa* | 4951 | 2 | 215 | 176.6 | 528 | 459 |
|  | 5355 | 2 | 375.3 | 12.4 | 297 | 249 |
|  | 5428 | 3 | 595.7 | 8.3 | 420 | 342 |
|  | 5449 | 2 | 491.7 | 11.1 | 984 | 981 |
|  | 5919 | 3 | 233 | 4.2 | 444 | 387 |
|  | 6003 | 4 | 194.3 | 7.3 | 225 | 219 |
|  | 6068 | 3 | 257.8 | 4.1 | 240 | 234 |
|  | 6387 | 2 | 452.1 | 362.9 | 774 | 762 |
|  | 6492 | 2 | 595.3 | 394.8 | 510 | 492 |
|  | 6498 | 3 | 548.1 | 13.2 | 282 | 282 |
|  | 7336 | 2 | 167.2 | 11.8 | 456 | 369 |
| *O. thyrsiflora* | 4951 | 2 | 253.8 | 238.2 | 459 | 459 |
|  | 5919 | 2 | 328.3 | 97.9 | 444 | 387 |
|  | 6003 | 2 | 205.3 | 188.4 | 219 | 210 |
|  | 6068 | 2 | 278.3 | 227 | 240 | 240 |
|  | 6387 | 2 | 593.6 | 382.6 | 774 | 774 |
|  | 6492 | 2 | 747.1 | 514.9 | 528 | 492 |
| *M. sikokianus* | 5821 | 2 | 667.2 | 482.7 | 423 | 399 |
|  | 5945 | 2 | 150 | 102.9 | 780 | 780 |
|  | 6068 | 2 | 219.3 | 17.4 | 252 | 249 |
|  | 6498 | 2 | 176.7 | 162.3 | 282 | 282 |
|  | 6854 | 2 | 41.5 | 13 | 651 | 615 |
| *H. erythrostictum* | 4691 | 2 | 94.5 | 73.6 | 531 | 531 |
|  | 4989 | 2 | 102.7 | 85.4 | 624 | 624 |
|  | 5034 | 2 | 157 | 84.3 | 732 | 636 |
|  | 5206 | 2 | 111.3 | 78.6 | 444 | 444 |
|  | 5280 | 2 | 603.5 | 334.4 | 885 | 813 |
|  | 5304 | 2 | 106.5 | 67.8 | 492 | 492 |
|  | 5347 | 3 | 94.4 | 38 | 210 | 192 |
|  | 5355 | 2 | 76.3 | 7.6 | 297 | 288 |
|  | 5460 | 2 | 78.3 | 33.1 | 390 | 378 |
|  | 5594 | 2 | 777 | 691.4 | 651 | 564 |
|  | 5620 | 2 | 230.4 | 168.1 | 1005 | 1002 |
|  | 5840 | 2 | 535.3 | 375.9 | 696 | 696 |
|  | 5857 | 3 | 170.2 | 95.2 | 657 | 615 |
|  | 5942 | 2 | 101.8 | 92.8 | 567 | 501 |
|  | 5945 | 3 | 505.6 | 116.3 | 777 | 693 |
|  | 5949 | 2 | 83.1 | 44.1 | 432 | 426 |
|  | 5958 | 2 | 23.7 | 4.8 | 345 | 285 |
|  | 6003 | 2 | 84.2 | 69.6 | 219 | 219 |
|  | 6034 | 2 | 195.5 | 20.3 | 498 | 483 |
|  | 6051 | 2 | 380.8 | 327.7 | 921 | 912 |
|  | 6114 | 2 | 34 | 33.2 | 567 | 567 |
|  | 6139 | 2 | 426.8 | 347.8 | 909 | 909 |
|  | 6238 | 2 | 610.4 | 112.5 | 690 | 591 |
|  | 6295 | 2 | 510.8 | 386.3 | 1158 | 1158 |
|  | 6401 | 2 | 100.8 | 41.1 | 540 | 540 |
|  | 6420 | 2 | 75.7 | 70.5 | 606 | 582 |
|  | 6492 | 2 | 247.4 | 164.2 | 528 | 510 |
|  | 6498 | 5 | 57.5 | 23.1 | 282 | 225 |
|  | 6528 | 2 | 32.1 | 27.7 | 354 | 330 |
|  | 6572 | 2 | 260.6 | 244.6 | 861 | 786 |
|  | 6875 | 2 | 42.8 | 17.4 | 387 | 387 |
|  | 6883 | 2 | 185.5 | 162.9 | 483 | 483 |
|  | 6909 | 2 | 157.1 | 142.2 | 525 | 507 |
|  | 6955 | 2 | 10.3 | 6.7 | 321 | 312 |
|  | 6992 | 2 | 250.8 | 219.8 | 384 | 357 |
|  | 7067 | 2 | 106.7 | 44 | 372 | 324 |
|  | 7333 | 4 | 75.8 | 39.1 | 396 | 321 |
| *H. pallenscens* | 4691 | 2 | 1587.2 | 1081.2 | 531 | 420 |
|  | 5206 | 2 | 413.8 | 325.6 | 444 | 444 |
|  | 5355 | 2 | 609.2 | 549.2 | 297 | 294 |
|  | 5460 | 2 | 373 | 324.4 | 348 | 348 |
|  | 5594 | 2 | 5765.1 | 2600.7 | 645 | 615 |
|  | 5949 | 2 | 1751 | 394.7 | 462 | 441 |
|  | 5958 | 2 | 356.7 | 64.9 | 333 | 306 |
|  | 6003 | 2 | 371 | 175.4 | 219 | 219 |
|  | 6295 | 2 | 2825.4 | 1959 | 1158 | 927 |
|  | 6498 | 5 | 6484.1 | 841 | 282 | 207 |
|  | 6883 | 2 | 1668.7 | 1284.5 | 441 | 441 |
|  | 7194 | 2 | 313.3 | 266.7 | 390 | 390 |
|  | 7333 | 2 | 5624.3 | 1017.6 | 324 | 321 |
| *H. spectabile* | 4951 | 3 | 134.3 | 6 | 459 | 420 |
|  | 5355 | 2 | 690.4 | 3.8 | 297 | 222 |
|  | 5594 | 3 | 1863.8 | 1411.6 | 648 | 543 |
|  | 5949 | 2 | 512.6 | 385.7 | 462 | 441 |
|  | 5958 | 2 | 357 | 22 | 288 | 264 |
|  | 6003 | 2 | 252.6 | 197.6 | 225 | 225 |
|  | 6068 | 5 | 1167.3 | 3.9 | 234 | 204 |
|  | 6387 | 3 | 1066.3 | 6.5 | 786 | 645 |
|  | 6498 | 5 | 1615.9 | 5.5 | 282 | 282 |
|  | 6620 | 3 | 372.4 | 6.3 | 501 | 474 |
|  | 6875 | 3 | 258 | 3.8 | 381 | 297 |
| *H. ussuriense* | 4951 | 2 | 667.6 | 287.4 | 459 | 459 |
|  | 5460 | 2 | 489.4 | 295.5 | 348 | 336 |
|  | 5536 | 2 | 619.8 | 462.8 | 369 | 366 |
|  | 5594 | 2 | 5507.9 | 5130.4 | 522 | 519 |
|  | 6068 | 3 | 589.9 | 330.6 | 231 | 222 |
|  | 6498 | 3 | 6404 | 737.3 | 282 | 282 |
| *H. verticillatum* | 4932 | 2 | 509 | 405.4 | 885 | 795 |
|  | 5018 | 2 | 484.7 | 390.1 | 1191 | 969 |
|  | 5347 | 3 | 239.7 | 94.3 | 210 | 192 |
|  | 5940 | 2 | 744.3 | 501 | 828 | 825 |
|  | 5942 | 2 | 107.5 | 15.6 | 552 | 513 |
|  | 5949 | 2 | 265.7 | 187.4 | 432 | 432 |
|  | 5950 | 2 | 582.7 | 465.8 | 573 | 540 |
|  | 6274 | 2 | 174.1 | 40.2 | 537 | 531 |
|  | 6496 | 2 | 462.2 | 155.3 | 588 | 585 |
|  | 6498 | 3 | 201.1 | 42.1 | 282 | 273 |
|  | 6865 | 2 | 120.8 | 80.6 | 537 | 453 |
|  | 6909 | 2 | 214.1 | 182.7 | 525 | 507 |
|  | 6992 | 2 | 706.5 | 577.5 | 384 | 369 |
| *H. viridescens* | 4951 | 2 | 265.5 | 6.7 | 459 | 423 |
|  | 5355 | 2 | 455.1 | 379.6 | 297 | 243 |
|  | 5428 | 2 | 390.5 | 5.1 | 426 | 426 |
|  | 5594 | 2 | 2018.8 | 1115.7 | 648 | 618 |
|  | 5919 | 2 | 380.8 | 4 | 435 | 348 |
|  | 5958 | 2 | 206.7 | 44.5 | 306 | 291 |
|  | 6003 | 3 | 447.5 | 181.9 | 219 | 219 |
|  | 6498 | 5 | 1778.8 | 716 | 282 | 219 |
|  | 6620 | 2 | 246.6 | 9.5 | 489 | 474 |
|  | 6782 | 2 | 1423.7 | 1194 | 459 | 402 |
|  | 7333 | 2 | 1134.4 | 4.9 | 399 | 384 |
| *H. viviparum* | 6498 | 2 | 6362.5 | 4968.9 | 282 | 255 |
| *S. taqueii* | 4691 | 2 | 29.2 | 28.4 | 531 | 531 |
|  | 4989 | 2 | 46 | 31.4 | 624 | 606 |
|  | 5034 | 2 | 59.9 | 28.2 | 732 | 636 |
|  | 5206 | 2 | 36.1 | 30 | 444 | 444 |
|  | 5347 | 3 | 34.4 | 14.7 | 210 | 192 |
|  | 5449 | 2 | 91 | 33.7 | 1047 | 912 |
|  | 5594 | 2 | 235.8 | 172 | 651 | 648 |
|  | 5620 | 2 | 76.7 | 58.7 | 1005 | 957 |
|  | 5840 | 2 | 187.5 | 166.6 | 825 | 825 |
|  | 5857 | 3 | 53.1 | 32.2 | 657 | 615 |
|  | 5942 | 2 | 30 | 27.1 | 552 | 546 |
|  | 5945 | 2 | 37.8 | 33.8 | 777 | 777 |
|  | 5949 | 3 | 30.3 | 15.8 | 432 | 426 |
|  | 6003 | 3 | 77 | 15.2 | 219 | 219 |
|  | 6034 | 2 | 60.2 | 12.6 | 495 | 483 |
|  | 6051 | 2 | 126.1 | 108.1 | 1179 | 1035 |
|  | 6139 | 2 | 136.3 | 94.7 | 909 | 909 |
|  | 6295 | 2 | 199.7 | 99.2 | 1158 | 1158 |
|  | 6401 | 2 | 32.5 | 16.8 | 540 | 540 |
|  | 6420 | 2 | 24.5 | 20.3 | 606 | 582 |
|  | 6498 | 5 | 19.8 | 8 | 282 | 225 |
|  | 6528 | 2 | 12.2 | 11.1 | 354 | 330 |
|  | 6532 | 2 | 579.2 | 178.3 | 246 | 216 |
|  | 6601 | 2 | 117.8 | 47.9 | 723 | 618 |
|  | 6875 | 2 | 14.9 | 6.5 | 387 | 387 |
|  | 6883 | 2 | 54 | 48 | 483 | 483 |
|  | 6909 | 2 | 51 | 45 | 525 | 507 |
|  | 6992 | 2 | 77.3 | 74.5 | 384 | 357 |
|  | 7333 | 2 | 42.8 | 35.5 | 396 | 336 |
| *P. aizoon* | 5304 | 2 | 722.1 | 404.8 | 474 | 465 |
|  | 5347 | 2 | 405.7 | 216.7 | 213 | 213 |
|  | 5355 | 2 | 784.3 | 129.4 | 300 | 300 |
|  | 5428 | 5 | 1382.8 | 26.5 | 420 | 366 |
|  | 5434 | 2 | 1602 | 178 | 558 | 546 |
|  | 5460 | 3 | 622 | 253 | 396 | 381 |
|  | 5858 | 2 | 876.6 | 188.8 | 597 | 489 |
|  | 5919 | 2 | 615.7 | 192.2 | 429 | 366 |
|  | 5941 | 3 | 1789.3 | 306 | 360 | 276 |
|  | 5958 | 2 | 707.3 | 50 | 336 | 324 |
|  | 6068 | 2 | 1158.4 | 166.8 | 219 | 219 |
|  | 6412 | 4 | 897.7 | 23.6 | 597 | 480 |
|  | 6492 | 2 | 1265.7 | 875.2 | 468 | 423 |
|  | 6498 | 4 | 2855.4 | 709.9 | 285 | 228 |
|  | 6532 | 2 | 1181.3 | 540.3 | 258 | 207 |
|  | 6636 | 6 | 679.3 | 163.4 | 603 | 474 |
|  | 6886 | 2 | 41.1 | 7.2 | 159 | 150 |
|  | 6955 | 2 | 936.7 | 643.2 | 315 | 312 |
|  | 6995 | 10 | 161.7 | 5.6 | 231 | 132 |
|  | 7325 | 3 | 302.6 | 192.4 | 456 | 423 |
|  | 7333 | 4 | 1204.5 | 154.6 | 402 | 336 |
|  | 7336 | 3 | 931.3 | 214.9 | 402 | 378 |
| *P. aizoon* var. *floribundus* | 5220 | 2 | 632.4 | 607.7 | 531 | 528 |
|  | 5304 | 2 | 700.8 | 387.8 | 465 | 465 |
|  | 5355 | 2 | 485.6 | 204.7 | 300 | 237 |
|  | 5428 | 5 | 1586.2 | 19.7 | 420 | 345 |
|  | 5460 | 2 | 1112.2 | 327 | 387 | 348 |
|  | 5919 | 2 | 369.7 | 100.8 | 435 | 348 |
|  | 5950 | 2 | 590.4 | 442.2 | 657 | 546 |
|  | 6412 | 2 | 1359.1 | 235.4 | 597 | 591 |
|  | 6430 | 2 | 217.2 | 118.8 | 255 | 255 |
|  | 6492 | 2 | 1207.8 | 565.8 | 501 | 501 |
|  | 6498 | 5 | 1365.4 | 297.3 | 285 | 216 |
|  | 6532 | 4 | 574.3 | 493.3 | 258 | 207 |
|  | 6636 | 3 | 693 | 254.1 | 609 | 555 |
|  | 6886 | 2 | 75.5 | 12.1 | 165 | 150 |
|  | 6955 | 4 | 440 | 164.6 | 312 | 282 |
|  | 6995 | 8 | 83.7 | 4.8 | 231 | 195 |
|  | 7325 | 2 | 807.5 | 192.1 | 453 | 435 |
|  | 7333 | 5 | 929 | 134.7 | 402 | 336 |
|  | 7336 | 3 | 507 | 234.3 | 441 | 351 |
|  | 7572 | 2 | 1228.2 | 840.2 | 648 | 591 |

Table S4. Sequence recovery and variation statistics at the 30% taxon completeness threshold.

| **Gene** | **Original Length** | **Original Sequences** | **Variable Sites** | **Variable Percentage** | **Filtered Sequences** | **Removed Sequences** |
| --- | --- | --- | --- | --- | --- | --- |
| 4471_supercontig | 1726 | 43 | 1051 | 60.9 | 34 | 9 |
| 4527_supercontig | 1635 | 43 | 797 | 48.7 | 40 | 3 |
| 4691_supercontig | 902 | 43 | 496 | 55.0 | 34 | 9 |
| 4724_supercontig | 607 | 39 | 345 | 56.8 | 34 | 5 |
| 4744_supercontig | 244 | 27 | 113 | 46.3 | 27 | 0 |
| 4757_supercontig | 1561 | 43 | 1001 | 64.1 | 39 | 4 |
| 4793_supercontig | 826 | 39 | 656 | 79.4 | 21 | 18 |
| 4796_supercontig | 1248 | 43 | 811 | 65.0 | 33 | 10 |
| 4802_supercontig | 2027 | 43 | 938 | 46.3 | 41 | 2 |
| 4806_supercontig | 664 | 28 | 307 | 46.2 | 14 | 14 |
| 4848_supercontig | 1816 | 43 | 659 | 36.3 | 43 | 0 |
| 4889_supercontig | 842 | 43 | 509 | 60.5 | 30 | 13 |
| 4890_supercontig | 548 | 16 | 311 | 56.8 | 9 | 7 |
| 4893_supercontig | 1556 | 43 | 873 | 56.1 | 25 | 18 |
| 4932_supercontig | 1341 | 43 | 597 | 44.5 | 36 | 7 |
| 4942_supercontig | 2014 | 43 | 1106 | 54.9 | 29 | 14 |
| 4951_supercontig | 709 | 43 | 391 | 55.1 | 38 | 5 |
| 4954_supercontig | 939 | 43 | 458 | 48.8 | 39 | 4 |
| 4989_supercontig | 1141 | 43 | 488 | 42.8 | 43 | 0 |
| 4992_supercontig | 978 | 43 | 505 | 51.6 | 29 | 14 |
| 5018_supercontig | 1786 | 43 | 885 | 49.6 | 35 | 8 |
| 5032_supercontig | 573 | 35 | 234 | 40.8 | 19 | 16 |
| 5034_supercontig | 1524 | 43 | 783 | 51.4 | 37 | 6 |
| 5038_supercontig | 1023 | 43 | 570 | 55.7 | 37 | 6 |
| 5064_supercontig | 262 | 24 | 228 | 87.0 | 15 | 9 |
| 5090_supercontig | 1833 | 43 | 1176 | 64.2 | 33 | 10 |
| 5116_supercontig | 1128 | 42 | 579 | 51.3 | 25 | 17 |
| 5123_supercontig | 1155 | 43 | 891 | 77.1 | 32 | 11 |
| 5131_supercontig | 354 | 27 | 181 | 51.1 | 8 | 19 |
| 5138_supercontig | 1407 | 43 | 678 | 48.2 | 36 | 7 |
| 5162_supercontig | 1048 | 43 | 376 | 35.9 | 40 | 3 |
| 5163_supercontig | 2485 | 43 | 1186 | 47.7 | 30 | 13 |
| 5168_supercontig | 706 | 24 | 272 | 38.5 | 14 | 10 |
| 5177_supercontig | 358 | 40 | 158 | 44.1 | 39 | 1 |
| 5188_supercontig | 1528 | 43 | 1115 | 73.0 | 38 | 5 |
| 5200_supercontig | 1369 | 43 | 851 | 62.2 | 37 | 6 |
| 5206_supercontig | 1131 | 43 | 527 | 46.6 | 39 | 4 |
| 5220_supercontig | 732 | 42 | 337 | 46.0 | 27 | 15 |
| 5257_supercontig | 1235 | 43 | 567 | 45.9 | 37 | 6 |
| 5264_supercontig | 1484 | 43 | 863 | 58.2 | 27 | 16 |
| 5273_supercontig | 412 | 29 | 189 | 45.9 | 9 | 20 |
| 5280_supercontig | 1757 | 42 | 756 | 43.0 | 37 | 5 |
| 5296_supercontig | 2295 | 43 | 1079 | 47.0 | 36 | 7 |
| 5299_supercontig | 1019 | 42 | 559 | 54.9 | 35 | 7 |
| 5304_supercontig | 2024 | 43 | 964 | 47.6 | 40 | 3 |
| 5318_supercontig | 1479 | 43 | 760 | 51.4 | 21 | 22 |
| 5326_supercontig | 1958 | 41 | 887 | 45.3 | 32 | 9 |
| 5328_supercontig | 779 | 43 | 384 | 49.3 | 35 | 8 |
| 5333_supercontig | 371 | 21 | 262 | 70.6 | 10 | 11 |
| 5335_supercontig | 1110 | 43 | 625 | 56.3 | 31 | 12 |
| 5339_supercontig | 1167 | 43 | 641 | 54.9 | 22 | 21 |
| 5343_supercontig | 900 | 43 | 375 | 41.7 | 37 | 6 |
| 5347_supercontig | 824 | 42 | 520 | 63.1 | 33 | 9 |
| 5348_supercontig | 448 | 35 | 202 | 45.1 | 23 | 12 |
| 5354_supercontig | 1303 | 42 | 721 | 55.3 | 31 | 11 |
| 5355_supercontig | 975 | 43 | 405 | 41.5 | 32 | 11 |
| 5357_supercontig | 527 | 32 | 285 | 54.1 | 19 | 13 |
| 5366_supercontig | 1806 | 43 | 1011 | 56.0 | 24 | 19 |
| 5398_supercontig | 2539 | 43 | 1237 | 48.7 | 38 | 5 |
| 5404_supercontig | 1178 | 36 | 607 | 51.5 | 20 | 16 |
| 5406_supercontig | 2757 | 43 | 1330 | 48.2 | 28 | 15 |
| 5421_supercontig | 751 | 43 | 372 | 49.5 | 28 | 15 |
| 5422_supercontig | 896 | 40 | 463 | 51.7 | 32 | 8 |
| 5426_supercontig | 2381 | 43 | 1124 | 47.2 | 42 | 1 |
| 5427_supercontig | 473 | 23 | 303 | 64.1 | 9 | 14 |
| 5428_supercontig | 699 | 43 | 284 | 40.6 | 37 | 6 |
| 5430_supercontig | 1192 | 32 | 714 | 59.9 | 20 | 12 |
| 5434_supercontig | 1372 | 43 | 857 | 62.5 | 29 | 14 |
| 5449_supercontig | 2271 | 43 | 1333 | 58.7 | 37 | 6 |
| 5454_supercontig | 3341 | 43 | 1592 | 47.7 | 40 | 3 |
| 5460_supercontig | 1015 | 43 | 597 | 58.8 | 40 | 3 |
| 5463_supercontig | 861 | 43 | 561 | 65.2 | 40 | 3 |
| 5464_supercontig | 1302 | 43 | 878 | 67.4 | 26 | 17 |
| 5469_supercontig | 1019 | 43 | 659 | 64.7 | 32 | 11 |
| 5477_supercontig | 645 | 39 | 418 | 64.8 | 23 | 16 |
| 5489_supercontig | 2041 | 43 | 1152 | 56.4 | 31 | 12 |
| 5502_supercontig | 2784 | 43 | 1511 | 54.3 | 33 | 10 |
| 5513_supercontig | 875 | 40 | 570 | 65.1 | 30 | 10 |
| 5528_supercontig | 4019 | 43 | 2090 | 52.0 | 37 | 6 |
| 5531_supercontig | 468 | 39 | 203 | 43.4 | 33 | 6 |
| 5536_supercontig | 784 | 31 | 365 | 46.6 | 18 | 13 |
| 5551_supercontig | 2053 | 42 | 1105 | 53.8 | 31 | 11 |
| 5554_supercontig | 1773 | 43 | 1120 | 63.2 | 33 | 10 |
| 5562_supercontig | 408 | 36 | 272 | 66.7 | 29 | 7 |
| 5578_supercontig | 232 | 16 | 66 | 28.4 | 15 | 1 |
| 5594_supercontig | 1039 | 43 | 551 | 53.0 | 38 | 5 |
| 5596_supercontig | 2244 | 42 | 729 | 32.5 | 35 | 7 |
| 5599_supercontig | 3588 | 43 | 1848 | 51.5 | 31 | 12 |
| 5614_supercontig | 2452 | 42 | 1251 | 51.0 | 41 | 1 |
| 5620_supercontig | 1156 | 43 | 483 | 41.8 | 40 | 3 |
| 5634_supercontig | 3071 | 42 | 1748 | 56.9 | 35 | 7 |
| 5639_supercontig | 888 | 40 | 443 | 49.9 | 27 | 13 |
| 5642_supercontig | 243 | 26 | 143 | 58.8 | 25 | 1 |
| 5644_supercontig | 2081 | 43 | 1193 | 57.3 | 41 | 2 |
| 5656_supercontig | 776 | 42 | 325 | 41.9 | 31 | 11 |
| 5660_supercontig | 527 | 36 | 148 | 28.1 | 23 | 13 |
| 5664_supercontig | 1747 | 43 | 962 | 55.1 | 37 | 6 |
| 5670_supercontig | 3918 | 43 | 1665 | 42.5 | 38 | 5 |
| 5699_supercontig | 383 | 36 | 273 | 71.3 | 22 | 14 |
| 5702_supercontig | 1250 | 43 | 536 | 42.9 | 22 | 21 |
| 5703_supercontig | 1338 | 41 | 689 | 51.5 | 22 | 19 |
| 5716_supercontig | 1934 | 43 | 1357 | 70.2 | 24 | 19 |
| 5721_supercontig | 1336 | 43 | 751 | 56.2 | 35 | 8 |
| 5733_supercontig | 2328 | 42 | 1415 | 60.8 | 28 | 14 |
| 5744_supercontig | 774 | 42 | 585 | 75.6 | 28 | 14 |
| 5770_supercontig | 877 | 43 | 522 | 59.5 | 32 | 11 |
| 5772_supercontig | 1187 | 43 | 710 | 59.8 | 25 | 18 |
| 5791_supercontig | 697 | 26 | 437 | 62.7 | 15 | 11 |
| 5802_supercontig | 1087 | 43 | 464 | 42.7 | 37 | 6 |
| 5815_supercontig | 768 | 42 | 461 | 60.0 | 32 | 10 |
| 5816_supercontig | 278 | 31 | 136 | 48.9 | 28 | 3 |
| 5821_supercontig | 926 | 41 | 524 | 56.6 | 25 | 16 |
| 5822_supercontig | 731 | 20 | 278 | 38.0 | 14 | 6 |
| 5840_supercontig | 1823 | 43 | 950 | 52.1 | 39 | 4 |
| 5841_supercontig | 1061 | 41 | 538 | 50.7 | 31 | 10 |
| 5842_supercontig | 575 | 41 | 214 | 37.2 | 16 | 25 |
| 5843_supercontig | 535 | 15 | 315 | 58.9 | 10 | 5 |
| 5849_supercontig | 894 | 42 | 415 | 46.4 | 35 | 7 |
| 5853_supercontig | 855 | 43 | 357 | 41.8 | 17 | 26 |
| 5857_supercontig | 1693 | 43 | 954 | 56.3 | 35 | 8 |
| 5858_supercontig | 1382 | 42 | 786 | 56.9 | 29 | 13 |
| 5859_supercontig | 1155 | 43 | 642 | 55.6 | 39 | 4 |
| 5865_supercontig | 413 | 41 | 209 | 50.6 | 24 | 17 |
| 5866_supercontig | 1005 | 43 | 471 | 46.9 | 30 | 13 |
| 5870_supercontig | 2102 | 43 | 1330 | 63.3 | 28 | 15 |
| 5893_supercontig | 556 | 28 | 271 | 48.7 | 17 | 11 |
| 5894_supercontig | 1397 | 43 | 932 | 66.7 | 34 | 9 |
| 5899_supercontig | 2096 | 43 | 1146 | 54.7 | 40 | 3 |
| 5910_supercontig | 1502 | 43 | 710 | 47.3 | 38 | 5 |
| 5913_supercontig | 1584 | 43 | 770 | 48.6 | 40 | 3 |
| 5918_supercontig | 761 | 40 | 424 | 55.7 | 20 | 20 |
| 5919_supercontig | 488 | 41 | 220 | 45.1 | 40 | 1 |
| 5921_supercontig | 6320 | 43 | 3326 | 52.6 | 30 | 13 |
| 5922_supercontig | 3217 | 43 | 1625 | 50.5 | 37 | 6 |
| 5926_supercontig | 1052 | 43 | 640 | 60.8 | 36 | 7 |
| 5933_supercontig | 1287 | 43 | 656 | 51.0 | 42 | 1 |
| 5936_supercontig | 1297 | 42 | 828 | 63.8 | 35 | 7 |
| 5940_supercontig | 1242 | 43 | 808 | 65.1 | 34 | 9 |
| 5941_supercontig | 881 | 42 | 667 | 75.7 | 29 | 13 |
| 5942_supercontig | 943 | 43 | 463 | 49.1 | 36 | 7 |
| 5943_supercontig | 4021 | 41 | 1944 | 48.3 | 39 | 2 |
| 5944_supercontig | 1066 | 43 | 684 | 64.2 | 37 | 6 |
| 5945_supercontig | 1931 | 43 | 1306 | 67.6 | 40 | 3 |
| 5949_supercontig | 1378 | 43 | 839 | 60.9 | 40 | 3 |
| 5950_supercontig | 1124 | 42 | 676 | 60.1 | 33 | 9 |
| 5958_supercontig | 482 | 40 | 184 | 38.2 | 34 | 6 |
| 5960_supercontig | 1311 | 43 | 655 | 50.0 | 41 | 2 |
| 5968_supercontig | 694 | 43 | 372 | 53.6 | 26 | 17 |
| 5974_supercontig | 1519 | 43 | 697 | 45.9 | 40 | 3 |
| 5977_supercontig | 677 | 42 | 243 | 35.9 | 20 | 22 |
| 5980_supercontig | 1114 | 43 | 682 | 61.2 | 38 | 5 |
| 5981_supercontig | 258 | 19 | 136 | 52.7 | 18 | 1 |
| 5990_supercontig | 216 | 24 | 154 | 71.3 | 24 | 0 |
| 6000_supercontig | 200 | 13 | 95 | 47.5 | 12 | 1 |
| 6003_supercontig | 794 | 30 | 498 | 62.7 | 21 | 9 |
| 6004_supercontig | 1103 | 43 | 478 | 43.3 | 36 | 7 |
| 6016_supercontig | 1717 | 43 | 949 | 55.3 | 37 | 6 |
| 6026_supercontig | 928 | 43 | 457 | 49.2 | 29 | 14 |
| 6029_supercontig | 1276 | 43 | 651 | 51.0 | 29 | 14 |
| 6034_supercontig | 1098 | 37 | 639 | 58.2 | 30 | 7 |
| 6036_supercontig | 2172 | 35 | 1042 | 48.0 | 31 | 4 |
| 6038_supercontig | 2275 | 43 | 1374 | 60.4 | 40 | 3 |
| 6041_supercontig | 2191 | 43 | 1097 | 50.1 | 40 | 3 |
| 6048_supercontig | 971 | 43 | 550 | 56.6 | 34 | 9 |
| 6050_supercontig | 729 | 32 | 459 | 63.0 | 18 | 14 |
| 6051_supercontig | 1365 | 43 | 689 | 50.5 | 42 | 1 |
| 6056_supercontig | 782 | 41 | 407 | 52.0 | 27 | 14 |
| 6064_supercontig | 1412 | 43 | 702 | 49.7 | 34 | 9 |
| 6068_supercontig | 988 | 43 | 564 | 57.1 | 35 | 8 |
| 6098_supercontig | 2753 | 43 | 1315 | 47.8 | 34 | 9 |
| 6110_supercontig | 499 | 39 | 212 | 42.5 | 30 | 9 |
| 6114_supercontig | 1704 | 43 | 962 | 56.5 | 27 | 16 |
| 6119_supercontig | 2041 | 43 | 1109 | 54.3 | 37 | 6 |
| 6128_supercontig | 1826 | 42 | 966 | 52.9 | 36 | 6 |
| 6130_supercontig | 1409 | 43 | 696 | 49.4 | 34 | 9 |
| 6139_supercontig | 2024 | 43 | 1043 | 51.5 | 40 | 3 |
| 6148_supercontig | 461 | 8 | 126 | 27.3 | 4 | 4 |
| 6150_supercontig | 191 | 23 | 59 | 30.9 | 21 | 2 |
| 6164_supercontig | 791 | 39 | 420 | 53.1 | 24 | 15 |
| 6176_supercontig | 1769 | 43 | 894 | 50.5 | 35 | 8 |
| 6198_supercontig | 780 | 24 | 589 | 75.5 | 15 | 9 |
| 6216_supercontig | 1283 | 43 | 595 | 46.4 | 32 | 11 |
| 6221_supercontig | 237 | 17 | 111 | 46.8 | 16 | 1 |
| 6226_supercontig | 995 | 43 | 498 | 50.1 | 36 | 7 |
| 6227_supercontig | 1691 | 43 | 938 | 55.5 | 37 | 6 |
| 6238_supercontig | 1262 | 43 | 567 | 44.9 | 38 | 5 |
| 6258_supercontig | 1058 | 43 | 722 | 68.2 | 35 | 8 |
| 6265_supercontig | 2001 | 42 | 1046 | 52.3 | 31 | 11 |
| 6270_supercontig | 916 | 16 | 507 | 55.3 | 9 | 7 |
| 6274_supercontig | 948 | 43 | 543 | 57.3 | 43 | 0 |
| 6282_supercontig | 1586 | 43 | 780 | 49.2 | 35 | 8 |
| 6284_supercontig | 1565 | 43 | 796 | 50.9 | 32 | 11 |
| 6295_supercontig | 1957 | 43 | 1159 | 59.2 | 39 | 4 |
| 6298_supercontig | 1066 | 43 | 582 | 54.6 | 33 | 10 |
| 6299_supercontig | 599 | 43 | 301 | 50.3 | 31 | 12 |
| 6303_supercontig | 1070 | 43 | 630 | 58.9 | 33 | 10 |
| 6318_supercontig | 1268 | 43 | 766 | 60.4 | 27 | 16 |
| 6320_supercontig | 2127 | 43 | 1144 | 53.8 | 38 | 5 |
| 6363_supercontig | 1644 | 43 | 949 | 57.7 | 41 | 2 |
| 6366_supercontig | 309 | 25 | 210 | 68.0 | 19 | 6 |
| 6373_supercontig | 848 | 42 | 504 | 59.4 | 26 | 16 |
| 6376_supercontig | 981 | 43 | 513 | 52.3 | 26 | 17 |
| 6378_supercontig | 1269 | 43 | 592 | 46.7 | 17 | 26 |
| 6379_supercontig | 980 | 42 | 616 | 62.9 | 34 | 8 |
| 6383_supercontig | 1888 | 43 | 974 | 51.6 | 36 | 7 |
| 6384_supercontig | 311 | 39 | 259 | 83.3 | 20 | 19 |
| 6387_supercontig | 959 | 43 | 461 | 48.1 | 42 | 1 |
| 6389_supercontig | 1553 | 33 | 866 | 55.8 | 29 | 4 |
| 6393_supercontig | 1005 | 43 | 591 | 58.8 | 35 | 8 |
| 6398_supercontig | 227 | 9 | 121 | 53.3 | 8 | 1 |
| 6401_supercontig | 700 | 43 | 342 | 48.9 | 37 | 6 |
| 6404_supercontig | 899 | 43 | 489 | 54.4 | 26 | 17 |
| 6405_supercontig | 1227 | 43 | 749 | 61.0 | 37 | 6 |
| 6406_supercontig | 340 | 7 | 201 | 59.1 | 5 | 2 |
| 6412_supercontig | 1182 | 43 | 523 | 44.2 | 38 | 5 |
| 6420_supercontig | 1396 | 42 | 659 | 47.2 | 38 | 4 |
| 6432_supercontig | 655 | 38 | 314 | 47.9 | 35 | 3 |
| 6439_supercontig | 835 | 43 | 470 | 56.3 | 29 | 14 |
| 6447_supercontig | 829 | 35 | 531 | 64.1 | 20 | 15 |
| 6448_supercontig | 836 | 39 | 411 | 49.2 | 31 | 8 |
| 6450_supercontig | 1504 | 43 | 775 | 51.5 | 35 | 8 |
| 6454_supercontig | 798 | 42 | 456 | 57.1 | 32 | 10 |
| 6457_supercontig | 228 | 30 | 86 | 37.7 | 30 | 0 |
| 6458_supercontig | 749 | 28 | 506 | 67.6 | 22 | 6 |
| 6459_supercontig | 1379 | 43 | 848 | 61.5 | 29 | 14 |
| 6460_supercontig | 393 | 41 | 244 | 62.1 | 40 | 1 |
| 6462_supercontig | 1778 | 43 | 954 | 53.7 | 40 | 3 |
| 6483_supercontig | 1108 | 43 | 620 | 56.0 | 31 | 12 |
| 6487_supercontig | 2775 | 43 | 1327 | 47.8 | 38 | 5 |
| 6488_supercontig | 1058 | 42 | 495 | 46.8 | 31 | 11 |
| 6492_supercontig | 1107 | 43 | 571 | 51.6 | 38 | 5 |
| 6494_supercontig | 1609 | 43 | 690 | 42.9 | 42 | 1 |
| 6496_supercontig | 2870 | 43 | 1647 | 57.4 | 34 | 9 |
| 6498_supercontig | 936 | 43 | 599 | 64.0 | 35 | 8 |
| 6500_supercontig | 2468 | 43 | 1258 | 51.0 | 39 | 4 |
| 6506_supercontig | 1257 | 43 | 866 | 68.9 | 20 | 23 |
| 6507_supercontig | 310 | 12 | 103 | 33.2 | 9 | 3 |
| 6527_supercontig | 2223 | 43 | 1094 | 49.2 | 41 | 2 |
| 6528_supercontig | 963 | 42 | 418 | 43.4 | 31 | 11 |
| 6531_supercontig | 784 | 42 | 469 | 59.8 | 31 | 11 |
| 6532_supercontig | 691 | 43 | 406 | 58.8 | 28 | 15 |
| 6533_supercontig | 1296 | 43 | 749 | 57.8 | 34 | 9 |
| 6538_supercontig | 827 | 43 | 431 | 52.1 | 34 | 9 |
| 6540_supercontig | 895 | 43 | 481 | 53.7 | 31 | 12 |
| 6544_supercontig | 1816 | 41 | 985 | 54.2 | 26 | 15 |
| 6550_supercontig | 377 | 26 | 186 | 49.3 | 25 | 1 |
| 6552_supercontig | 224 | 30 | 89 | 39.7 | 16 | 14 |
| 6557_supercontig | 731 | 42 | 379 | 51.8 | 27 | 15 |
| 6559_supercontig | 419 | 25 | 220 | 52.5 | 19 | 6 |
| 6563_supercontig | 1386 | 43 | 926 | 66.8 | 37 | 6 |
| 6570_supercontig | 192 | 12 | 160 | 83.3 | 12 | 0 |
| 6572_supercontig | 1767 | 43 | 748 | 42.3 | 35 | 8 |
| 6601_supercontig | 2573 | 43 | 1496 | 58.1 | 41 | 2 |
| 6620_supercontig | 653 | 43 | 311 | 47.6 | 43 | 0 |
| 6631_supercontig | 517 | 35 | 194 | 37.5 | 25 | 10 |
| 6636_supercontig | 1250 | 43 | 663 | 53.0 | 39 | 4 |
| 6639_supercontig | 1307 | 43 | 696 | 53.3 | 36 | 7 |
| 6641_supercontig | 1115 | 43 | 613 | 55.0 | 39 | 4 |
| 6649_supercontig | 617 | 43 | 426 | 69.0 | 34 | 9 |
| 6652_supercontig | 1782 | 43 | 849 | 47.6 | 35 | 8 |
| 6660_supercontig | 1018 | 40 | 327 | 32.1 | 31 | 9 |
| 6667_supercontig | 1736 | 43 | 830 | 47.8 | 31 | 12 |
| 6679_supercontig | 305 | 19 | 188 | 61.6 | 9 | 10 |
| 6685_supercontig | 1964 | 43 | 1193 | 60.7 | 34 | 9 |
| 6689_supercontig | 1858 | 43 | 982 | 52.9 | 40 | 3 |
| 6713_supercontig | 776 | 27 | 324 | 41.8 | 22 | 5 |
| 6717_supercontig | 1690 | 43 | 773 | 45.7 | 40 | 3 |
| 6733_supercontig | 414 | 38 | 211 | 51.0 | 31 | 7 |
| 6738_supercontig | 353 | 40 | 272 | 77.1 | 31 | 9 |
| 6746_supercontig | 930 | 43 | 671 | 72.2 | 32 | 11 |
| 6779_supercontig | 1246 | 43 | 561 | 45.0 | 20 | 23 |
| 6780_supercontig | 529 | 42 | 219 | 41.4 | 31 | 11 |
| 6782_supercontig | 978 | 43 | 604 | 61.8 | 36 | 7 |
| 6791_supercontig | 216 | 23 | 130 | 60.2 | 23 | 0 |
| 6792_supercontig | 556 | 43 | 314 | 56.5 | 20 | 23 |
| 6797_supercontig | 1702 | 43 | 888 | 52.2 | 26 | 17 |
| 6825_supercontig | 1325 | 43 | 658 | 49.7 | 38 | 5 |
| 6848_supercontig | 464 | 32 | 254 | 54.7 | 20 | 12 |
| 6854_supercontig | 1655 | 43 | 1231 | 74.4 | 42 | 1 |
| 6859_supercontig | 1767 | 43 | 1008 | 57.0 | 28 | 15 |
| 6860_supercontig | 481 | 40 | 178 | 37.0 | 24 | 16 |
| 6864_supercontig | 743 | 15 | 323 | 43.5 | 11 | 4 |
| 6865_supercontig | 1521 | 43 | 770 | 50.6 | 30 | 13 |
| 6875_supercontig | 877 | 43 | 322 | 36.7 | 35 | 8 |
| 6882_supercontig | 707 | 39 | 368 | 52.1 | 31 | 8 |
| 6883_supercontig | 1071 | 43 | 547 | 51.1 | 38 | 5 |
| 6886_supercontig | 226 | 19 | 102 | 45.1 | 18 | 1 |
| 6909_supercontig | 1695 | 43 | 1005 | 59.3 | 40 | 3 |
| 6913_supercontig | 2599 | 43 | 1602 | 61.6 | 37 | 6 |
| 6914_supercontig | 602 | 34 | 247 | 41.0 | 18 | 16 |
| 6924_supercontig | 2487 | 43 | 1289 | 51.8 | 39 | 4 |
| 6933_supercontig | 968 | 27 | 275 | 28.4 | 14 | 13 |
| 6946_supercontig | 906 | 43 | 557 | 61.5 | 33 | 10 |
| 6947_supercontig | 2371 | 40 | 1210 | 51.0 | 37 | 3 |
| 6954_supercontig | 719 | 35 | 407 | 56.6 | 29 | 6 |
| 6955_supercontig | 680 | 39 | 364 | 53.5 | 28 | 11 |
| 6958_supercontig | 671 | 40 | 305 | 45.5 | 27 | 13 |
| 6961_supercontig | 1249 | 43 | 595 | 47.6 | 35 | 8 |
| 6962_supercontig | 504 | 43 | 236 | 46.8 | 32 | 11 |
| 6968_supercontig | 1185 | 19 | 447 | 37.7 | 13 | 6 |
| 6978_supercontig | 1849 | 43 | 955 | 51.6 | 31 | 12 |
| 6979_supercontig | 382 | 23 | 122 | 31.9 | 18 | 5 |
| 6992_supercontig | 1554 | 43 | 987 | 63.5 | 38 | 5 |
| 6995_supercontig | 310 | 17 | 169 | 54.5 | 17 | 0 |
| 7013_supercontig | 931 | 39 | 506 | 54.4 | 33 | 6 |
| 7021_supercontig | 3026 | 43 | 1687 | 55.8 | 39 | 4 |
| 7028_supercontig | 232 | 9 | 64 | 27.6 | 9 | 0 |
| 7029_supercontig | 1431 | 43 | 761 | 53.2 | 37 | 6 |
| 7067_supercontig | 874 | 27 | 305 | 34.9 | 20 | 7 |
| 7111_supercontig | 718 | 41 | 363 | 50.6 | 23 | 18 |
| 7128_supercontig | 976 | 41 | 385 | 39.4 | 31 | 10 |
| 7135_supercontig | 377 | 14 | 222 | 58.9 | 9 | 5 |
| 7136_supercontig | 959 | 43 | 485 | 50.6 | 29 | 14 |
| 7141_supercontig | 697 | 43 | 374 | 53.7 | 36 | 7 |
| 7174_supercontig | 324 | 28 | 172 | 53.1 | 24 | 4 |
| 7194_supercontig | 904 | 42 | 471 | 52.1 | 38 | 4 |
| 7241_supercontig | 458 | 9 | 266 | 58.1 | 6 | 3 |
| 7273_supercontig | 190 | 16 | 105 | 55.3 | 16 | 0 |
| 7279_supercontig | 1159 | 42 | 824 | 71.1 | 35 | 7 |
| 7313_supercontig | 1637 | 43 | 854 | 52.2 | 38 | 5 |
| 7325_supercontig | 426 | 32 | 263 | 61.7 | 27 | 5 |
| 7333_supercontig | 501 | 43 | 190 | 37.9 | 41 | 2 |
| 7336_supercontig | 468 | 36 | 195 | 41.7 | 34 | 2 |
| 7361_supercontig | 361 | 36 | 217 | 60.1 | 23 | 13 |
| 7363_supercontig | 1045 | 41 | 562 | 53.8 | 39 | 2 |
| 7367_supercontig | 276 | 31 | 138 | 50.0 | 30 | 1 |
| 7371_supercontig | 321 | 34 | 175 | 54.5 | 31 | 3 |
| 7572_supercontig | 1986 | 43 | 1056 | 53.2 | 42 | 1 |
| 7577_supercontig | 457 | 38 | 226 | 49.5 | 25 | 13 |
| 7583_supercontig | 472 | 33 | 145 | 30.7 | 22 | 11 |
| 7602_supercontig | 961 | 43 | 462 | 48.1 | 38 | 5 |
| 7628_supercontig | 795 | 35 | 290 | 36.5 | 25 | 10 |

Table S5. Sequence recovery and variation statistics at the 50% taxon completeness threshold.

| **Gene** | **Original Length** | **Original Sequences** | **Variable Sites** | **Variable Percentage** | **Filtered Sequences** | **Removed Sequences** |
| --- | --- | --- | --- | --- | --- | --- |
| 4471_supercontig | 1726 | 43 | 1051 | 60.9 | 38 | 5 |
| 4527_supercontig | 1635 | 43 | 797 | 48.7 | 42 | 1 |
| 4691_supercontig | 902 | 43 | 496 | 55.0 | 38 | 5 |
| 4724_supercontig | 607 | 39 | 345 | 56.8 | 38 | 1 |
| 4744_supercontig | 244 | 27 | 113 | 46.3 | 27 | 0 |
| 4757_supercontig | 1561 | 43 | 1001 | 64.1 | 43 | 0 |
| 4793_supercontig | 826 | 39 | 656 | 79.4 | 33 | 6 |
| 4796_supercontig | 1248 | 43 | 811 | 65.0 | 40 | 3 |
| 4802_supercontig | 2027 | 43 | 938 | 46.3 | 43 | 0 |
| 4806_supercontig | 664 | 28 | 307 | 46.2 | 23 | 5 |
| 4848_supercontig | 1816 | 43 | 659 | 36.3 | 43 | 0 |
| 4889_supercontig | 842 | 43 | 509 | 60.5 | 34 | 9 |
| 4890_supercontig | 548 | 16 | 311 | 56.8 | 13 | 3 |
| 4893_supercontig | 1556 | 43 | 873 | 56.1 | 32 | 11 |
| 4932_supercontig | 1341 | 43 | 597 | 44.5 | 42 | 1 |
| 4942_supercontig | 2014 | 43 | 1106 | 54.9 | 38 | 5 |
| 4951_supercontig | 709 | 43 | 391 | 55.1 | 43 | 0 |
| 4954_supercontig | 939 | 43 | 458 | 48.8 | 42 | 1 |
| 4989_supercontig | 1141 | 43 | 488 | 42.8 | 43 | 0 |
| 4992_supercontig | 978 | 43 | 505 | 51.6 | 39 | 4 |
| 5018_supercontig | 1786 | 43 | 885 | 49.6 | 43 | 0 |
| 5032_supercontig | 573 | 35 | 234 | 40.8 | 24 | 11 |
| 5034_supercontig | 1524 | 43 | 783 | 51.4 | 43 | 0 |
| 5038_supercontig | 1023 | 43 | 570 | 55.7 | 43 | 0 |
| 5064_supercontig | 262 | 24 | 228 | 87.0 | 18 | 6 |
| 5090_supercontig | 1833 | 43 | 1176 | 64.2 | 34 | 9 |
| 5116_supercontig | 1128 | 42 | 579 | 51.3 | 29 | 13 |
| 5123_supercontig | 1155 | 43 | 891 | 77.1 | 42 | 1 |
| 5131_supercontig | 354 | 27 | 181 | 51.1 | 17 | 10 |
| 5138_supercontig | 1407 | 43 | 678 | 48.2 | 39 | 4 |
| 5162_supercontig | 1048 | 43 | 376 | 35.9 | 42 | 1 |
| 5163_supercontig | 2485 | 43 | 1186 | 47.7 | 41 | 2 |
| 5168_supercontig | 706 | 24 | 272 | 38.5 | 15 | 9 |
| 5177_supercontig | 358 | 40 | 158 | 44.1 | 40 | 0 |
| 5188_supercontig | 1528 | 43 | 1115 | 73.0 | 41 | 2 |
| 5200_supercontig | 1369 | 43 | 851 | 62.2 | 41 | 2 |
| 5206_supercontig | 1131 | 43 | 527 | 46.6 | 41 | 2 |
| 5220_supercontig | 732 | 42 | 337 | 46.0 | 35 | 7 |
| 5257_supercontig | 1235 | 43 | 567 | 45.9 | 43 | 0 |
| 5264_supercontig | 1484 | 43 | 863 | 58.2 | 39 | 4 |
| 5271_supercontig | 497 | 4 | 149 | 30.0 | 4 | 0 |
| 5273_supercontig | 412 | 29 | 189 | 45.9 | 19 | 10 |
| 5280_supercontig | 1757 | 42 | 756 | 43.0 | 42 | 0 |
| 5296_supercontig | 2295 | 43 | 1079 | 47.0 | 40 | 3 |
| 5299_supercontig | 1019 | 42 | 559 | 54.9 | 40 | 2 |
| 5304_supercontig | 2024 | 43 | 964 | 47.6 | 43 | 0 |
| 5318_supercontig | 1479 | 43 | 760 | 51.4 | 36 | 7 |
| 5326_supercontig | 1958 | 41 | 887 | 45.3 | 40 | 1 |
| 5328_supercontig | 779 | 43 | 384 | 49.3 | 42 | 1 |
| 5333_supercontig | 371 | 21 | 262 | 70.6 | 15 | 6 |
| 5335_supercontig | 1110 | 43 | 625 | 56.3 | 40 | 3 |
| 5339_supercontig | 1167 | 43 | 641 | 54.9 | 38 | 5 |
| 5343_supercontig | 900 | 43 | 375 | 41.7 | 43 | 0 |
| 5347_supercontig | 824 | 42 | 520 | 63.1 | 40 | 2 |
| 5348_supercontig | 448 | 35 | 202 | 45.1 | 24 | 11 |
| 5354_supercontig | 1303 | 42 | 721 | 55.3 | 38 | 4 |
| 5355_supercontig | 975 | 43 | 405 | 41.5 | 40 | 3 |
| 5357_supercontig | 527 | 32 | 285 | 54.1 | 29 | 3 |
| 5366_supercontig | 1806 | 43 | 1011 | 56.0 | 39 | 4 |
| 5398_supercontig | 2539 | 43 | 1237 | 48.7 | 43 | 0 |
| 5404_supercontig | 1178 | 36 | 607 | 51.5 | 24 | 12 |
| 5406_supercontig | 2757 | 43 | 1330 | 48.2 | 39 | 4 |
| 5421_supercontig | 751 | 43 | 372 | 49.5 | 34 | 9 |
| 5422_supercontig | 896 | 40 | 463 | 51.7 | 36 | 4 |
| 5426_supercontig | 2381 | 43 | 1124 | 47.2 | 43 | 0 |
| 5427_supercontig | 473 | 23 | 303 | 64.1 | 12 | 11 |
| 5428_supercontig | 699 | 43 | 284 | 40.6 | 40 | 3 |
| 5430_supercontig | 1192 | 32 | 714 | 59.9 | 22 | 10 |
| 5434_supercontig | 1372 | 43 | 857 | 62.5 | 43 | 0 |
| 5449_supercontig | 2271 | 43 | 1333 | 58.7 | 43 | 0 |
| 5454_supercontig | 3341 | 43 | 1592 | 47.7 | 43 | 0 |
| 5460_supercontig | 1015 | 43 | 597 | 58.8 | 41 | 2 |
| 5463_supercontig | 861 | 43 | 561 | 65.2 | 43 | 0 |
| 5464_supercontig | 1302 | 43 | 878 | 67.4 | 38 | 5 |
| 5469_supercontig | 1019 | 43 | 659 | 64.7 | 39 | 4 |
| 5477_supercontig | 645 | 39 | 418 | 64.8 | 32 | 7 |
| 5489_supercontig | 2041 | 43 | 1152 | 56.4 | 40 | 3 |
| 5502_supercontig | 2784 | 43 | 1511 | 54.3 | 42 | 1 |
| 5513_supercontig | 875 | 40 | 570 | 65.1 | 36 | 4 |
| 5528_supercontig | 4019 | 43 | 2090 | 52.0 | 43 | 0 |
| 5531_supercontig | 468 | 39 | 203 | 43.4 | 37 | 2 |
| 5536_supercontig | 784 | 31 | 365 | 46.6 | 22 | 9 |
| 5551_supercontig | 2053 | 42 | 1105 | 53.8 | 39 | 3 |
| 5554_supercontig | 1773 | 43 | 1120 | 63.2 | 41 | 2 |
| 5562_supercontig | 408 | 36 | 272 | 66.7 | 33 | 3 |
| 5578_supercontig | 232 | 16 | 66 | 28.4 | 16 | 0 |
| 5594_supercontig | 1039 | 43 | 551 | 53.0 | 43 | 0 |
| 5596_supercontig | 2244 | 42 | 729 | 32.5 | 39 | 3 |
| 5599_supercontig | 3588 | 43 | 1848 | 51.5 | 41 | 2 |
| 5614_supercontig | 2452 | 42 | 1251 | 51.0 | 42 | 0 |
| 5620_supercontig | 1156 | 43 | 483 | 41.8 | 42 | 1 |
| 5634_supercontig | 3071 | 42 | 1748 | 56.9 | 40 | 2 |
| 5639_supercontig | 888 | 40 | 443 | 49.9 | 33 | 7 |
| 5642_supercontig | 243 | 26 | 143 | 58.8 | 25 | 1 |
| 5644_supercontig | 2081 | 43 | 1193 | 57.3 | 43 | 0 |
| 5656_supercontig | 776 | 42 | 325 | 41.9 | 35 | 7 |
| 5660_supercontig | 527 | 36 | 148 | 28.1 | 28 | 8 |
| 5664_supercontig | 1747 | 43 | 962 | 55.1 | 41 | 2 |
| 5670_supercontig | 3918 | 43 | 1665 | 42.5 | 43 | 0 |
| 5699_supercontig | 383 | 36 | 273 | 71.3 | 30 | 6 |
| 5702_supercontig | 1250 | 43 | 536 | 42.9 | 29 | 14 |
| 5703_supercontig | 1338 | 41 | 689 | 51.5 | 35 | 6 |
| 5716_supercontig | 1934 | 43 | 1357 | 70.2 | 39 | 4 |
| 5721_supercontig | 1336 | 43 | 751 | 56.2 | 40 | 3 |
| 5733_supercontig | 2328 | 42 | 1415 | 60.8 | 35 | 7 |
| 5744_supercontig | 774 | 42 | 585 | 75.6 | 38 | 4 |
| 5770_supercontig | 877 | 43 | 522 | 59.5 | 37 | 6 |
| 5772_supercontig | 1187 | 43 | 710 | 59.8 | 38 | 5 |
| 5791_supercontig | 697 | 26 | 437 | 62.7 | 19 | 7 |
| 5802_supercontig | 1087 | 43 | 464 | 42.7 | 42 | 1 |
| 5815_supercontig | 768 | 42 | 461 | 60.0 | 38 | 4 |
| 5816_supercontig | 278 | 31 | 136 | 48.9 | 30 | 1 |
| 5821_supercontig | 926 | 41 | 524 | 56.6 | 37 | 4 |
| 5822_supercontig | 731 | 20 | 278 | 38.0 | 16 | 4 |
| 5840_supercontig | 1823 | 43 | 950 | 52.1 | 42 | 1 |
| 5841_supercontig | 1061 | 41 | 538 | 50.7 | 37 | 4 |
| 5842_supercontig | 575 | 41 | 214 | 37.2 | 24 | 17 |
| 5843_supercontig | 535 | 15 | 315 | 58.9 | 10 | 5 |
| 5849_supercontig | 894 | 42 | 415 | 46.4 | 41 | 1 |
| 5853_supercontig | 855 | 43 | 357 | 41.8 | 32 | 11 |
| 5857_supercontig | 1693 | 43 | 954 | 56.3 | 40 | 3 |
| 5858_supercontig | 1382 | 42 | 786 | 56.9 | 40 | 2 |
| 5859_supercontig | 1155 | 43 | 642 | 55.6 | 43 | 0 |
| 5865_supercontig | 413 | 41 | 209 | 50.6 | 36 | 5 |
| 5866_supercontig | 1005 | 43 | 471 | 46.9 | 41 | 2 |
| 5870_supercontig | 2102 | 43 | 1330 | 63.3 | 39 | 4 |
| 5893_supercontig | 556 | 28 | 271 | 48.7 | 22 | 6 |
| 5894_supercontig | 1397 | 43 | 932 | 66.7 | 40 | 3 |
| 5899_supercontig | 2096 | 43 | 1146 | 54.7 | 43 | 0 |
| 5910_supercontig | 1502 | 43 | 710 | 47.3 | 43 | 0 |
| 5913_supercontig | 1584 | 43 | 770 | 48.6 | 43 | 0 |
| 5918_supercontig | 761 | 40 | 424 | 55.7 | 28 | 12 |
| 5919_supercontig | 488 | 41 | 220 | 45.1 | 40 | 1 |
| 5921_supercontig | 6320 | 43 | 3326 | 52.6 | 36 | 7 |
| 5922_supercontig | 3217 | 43 | 1625 | 50.5 | 42 | 1 |
| 5926_supercontig | 1052 | 43 | 640 | 60.8 | 42 | 1 |
| 5933_supercontig | 1287 | 43 | 656 | 51.0 | 43 | 0 |
| 5936_supercontig | 1297 | 42 | 828 | 63.8 | 42 | 0 |
| 5940_supercontig | 1242 | 43 | 808 | 65.1 | 41 | 2 |
| 5941_supercontig | 881 | 42 | 667 | 75.7 | 38 | 4 |
| 5942_supercontig | 943 | 43 | 463 | 49.1 | 43 | 0 |
| 5943_supercontig | 4021 | 41 | 1944 | 48.3 | 41 | 0 |
| 5944_supercontig | 1066 | 43 | 684 | 64.2 | 42 | 1 |
| 5945_supercontig | 1931 | 43 | 1306 | 67.6 | 43 | 0 |
| 5949_supercontig | 1378 | 43 | 839 | 60.9 | 43 | 0 |
| 5950_supercontig | 1124 | 42 | 676 | 60.1 | 42 | 0 |
| 5958_supercontig | 482 | 40 | 184 | 38.2 | 39 | 1 |
| 5960_supercontig | 1311 | 43 | 655 | 50.0 | 43 | 0 |
| 5968_supercontig | 694 | 43 | 372 | 53.6 | 38 | 5 |
| 5974_supercontig | 1519 | 43 | 697 | 45.9 | 43 | 0 |
| 5977_supercontig | 677 | 42 | 243 | 35.9 | 30 | 12 |
| 5980_supercontig | 1114 | 43 | 682 | 61.2 | 41 | 2 |
| 5981_supercontig | 258 | 19 | 136 | 52.7 | 18 | 1 |
| 5990_supercontig | 216 | 24 | 154 | 71.3 | 24 | 0 |
| 6000_supercontig | 200 | 13 | 95 | 47.5 | 12 | 1 |
| 6003_supercontig | 794 | 30 | 498 | 62.7 | 27 | 3 |
| 6004_supercontig | 1103 | 43 | 478 | 43.3 | 41 | 2 |
| 6016_supercontig | 1717 | 43 | 949 | 55.3 | 40 | 3 |
| 6026_supercontig | 928 | 43 | 457 | 49.2 | 37 | 6 |
| 6029_supercontig | 1276 | 43 | 651 | 51.0 | 41 | 2 |
| 6034_supercontig | 1098 | 37 | 639 | 58.2 | 35 | 2 |
| 6036_supercontig | 2172 | 35 | 1042 | 48.0 | 34 | 1 |
| 6038_supercontig | 2275 | 43 | 1374 | 60.4 | 42 | 1 |
| 6041_supercontig | 2191 | 43 | 1097 | 50.1 | 42 | 1 |
| 6048_supercontig | 971 | 43 | 550 | 56.6 | 40 | 3 |
| 6050_supercontig | 729 | 32 | 459 | 63.0 | 25 | 7 |
| 6051_supercontig | 1365 | 43 | 689 | 50.5 | 43 | 0 |
| 6056_supercontig | 782 | 41 | 407 | 52.0 | 34 | 7 |
| 6064_supercontig | 1412 | 43 | 702 | 49.7 | 41 | 2 |
| 6068_supercontig | 988 | 43 | 564 | 57.1 | 42 | 1 |
| 6098_supercontig | 2753 | 43 | 1315 | 47.8 | 42 | 1 |
| 6110_supercontig | 499 | 39 | 212 | 42.5 | 34 | 5 |
| 6114_supercontig | 1704 | 43 | 962 | 56.5 | 40 | 3 |
| 6119_supercontig | 2041 | 43 | 1109 | 54.3 | 41 | 2 |
| 6128_supercontig | 1826 | 42 | 966 | 52.9 | 41 | 1 |
| 6130_supercontig | 1409 | 43 | 696 | 49.4 | 40 | 3 |
| 6139_supercontig | 2024 | 43 | 1043 | 51.5 | 43 | 0 |
| 6148_supercontig | 461 | 8 | 126 | 27.3 | 6 | 2 |
| 6150_supercontig | 191 | 23 | 59 | 30.9 | 22 | 1 |
| 6164_supercontig | 791 | 39 | 420 | 53.1 | 25 | 14 |
| 6176_supercontig | 1769 | 43 | 894 | 50.5 | 42 | 1 |
| 6198_supercontig | 780 | 24 | 589 | 75.5 | 20 | 4 |
| 6216_supercontig | 1283 | 43 | 595 | 46.4 | 35 | 8 |
| 6221_supercontig | 237 | 17 | 111 | 46.8 | 16 | 1 |
| 6226_supercontig | 995 | 43 | 498 | 50.1 | 42 | 1 |
| 6227_supercontig | 1691 | 43 | 938 | 55.5 | 41 | 2 |
| 6238_supercontig | 1262 | 43 | 567 | 44.9 | 43 | 0 |
| 6258_supercontig | 1058 | 43 | 722 | 68.2 | 41 | 2 |
| 6265_supercontig | 2001 | 42 | 1046 | 52.3 | 38 | 4 |
| 6270_supercontig | 916 | 16 | 507 | 55.3 | 11 | 5 |
| 6274_supercontig | 948 | 43 | 543 | 57.3 | 43 | 0 |
| 6282_supercontig | 1586 | 43 | 780 | 49.2 | 41 | 2 |
| 6284_supercontig | 1565 | 43 | 796 | 50.9 | 41 | 2 |
| 6295_supercontig | 1957 | 43 | 1159 | 59.2 | 43 | 0 |
| 6298_supercontig | 1066 | 43 | 582 | 54.6 | 42 | 1 |
| 6299_supercontig | 599 | 43 | 301 | 50.3 | 39 | 4 |
| 6303_supercontig | 1070 | 43 | 630 | 58.9 | 40 | 3 |
| 6318_supercontig | 1268 | 43 | 766 | 60.4 | 36 | 7 |
| 6320_supercontig | 2127 | 43 | 1144 | 53.8 | 41 | 2 |
| 6363_supercontig | 1644 | 43 | 949 | 57.7 | 42 | 1 |
| 6366_supercontig | 309 | 25 | 210 | 68.0 | 25 | 0 |
| 6373_supercontig | 848 | 42 | 504 | 59.4 | 34 | 8 |
| 6376_supercontig | 981 | 43 | 513 | 52.3 | 36 | 7 |
| 6378_supercontig | 1269 | 43 | 592 | 46.7 | 33 | 10 |
| 6379_supercontig | 980 | 42 | 616 | 62.9 | 38 | 4 |
| 6383_supercontig | 1888 | 43 | 974 | 51.6 | 42 | 1 |
| 6384_supercontig | 311 | 39 | 259 | 83.3 | 25 | 14 |
| 6387_supercontig | 959 | 43 | 461 | 48.1 | 43 | 0 |
| 6389_supercontig | 1553 | 33 | 866 | 55.8 | 33 | 0 |
| 6393_supercontig | 1005 | 43 | 591 | 58.8 | 38 | 5 |
| 6398_supercontig | 227 | 9 | 121 | 53.3 | 9 | 0 |
| 6401_supercontig | 700 | 43 | 342 | 48.9 | 41 | 2 |
| 6404_supercontig | 899 | 43 | 489 | 54.4 | 35 | 8 |
| 6405_supercontig | 1227 | 43 | 749 | 61.0 | 41 | 2 |
| 6406_supercontig | 340 | 7 | 201 | 59.1 | 7 | 0 |
| 6412_supercontig | 1182 | 43 | 523 | 44.2 | 43 | 0 |
| 6420_supercontig | 1396 | 42 | 659 | 47.2 | 42 | 0 |
| 6432_supercontig | 655 | 38 | 314 | 47.9 | 36 | 2 |
| 6439_supercontig | 835 | 43 | 470 | 56.3 | 37 | 6 |
| 6447_supercontig | 829 | 35 | 531 | 64.1 | 32 | 3 |
| 6448_supercontig | 836 | 39 | 411 | 49.2 | 37 | 2 |
| 6450_supercontig | 1504 | 43 | 775 | 51.5 | 42 | 1 |
| 6454_supercontig | 798 | 42 | 456 | 57.1 | 37 | 5 |
| 6457_supercontig | 228 | 30 | 86 | 37.7 | 30 | 0 |
| 6458_supercontig | 749 | 28 | 506 | 67.6 | 23 | 5 |
| 6459_supercontig | 1379 | 43 | 848 | 61.5 | 38 | 5 |
| 6460_supercontig | 393 | 41 | 244 | 62.1 | 41 | 0 |
| 6462_supercontig | 1778 | 43 | 954 | 53.7 | 43 | 0 |
| 6483_supercontig | 1108 | 43 | 620 | 56.0 | 38 | 5 |
| 6487_supercontig | 2775 | 43 | 1327 | 47.8 | 41 | 2 |
| 6488_supercontig | 1058 | 42 | 495 | 46.8 | 33 | 9 |
| 6492_supercontig | 1107 | 43 | 571 | 51.6 | 41 | 2 |
| 6494_supercontig | 1609 | 43 | 690 | 42.9 | 43 | 0 |
| 6496_supercontig | 2870 | 43 | 1647 | 57.4 | 42 | 1 |
| 6498_supercontig | 936 | 43 | 599 | 64.0 | 41 | 2 |
| 6500_supercontig | 2468 | 43 | 1258 | 51.0 | 43 | 0 |
| 6506_supercontig | 1257 | 43 | 866 | 68.9 | 32 | 11 |
| 6507_supercontig | 310 | 12 | 103 | 33.2 | 10 | 2 |
| 6527_supercontig | 2223 | 43 | 1094 | 49.2 | 43 | 0 |
| 6528_supercontig | 963 | 42 | 418 | 43.4 | 41 | 1 |
| 6531_supercontig | 784 | 42 | 469 | 59.8 | 38 | 4 |
| 6532_supercontig | 691 | 43 | 406 | 58.8 | 32 | 11 |
| 6533_supercontig | 1296 | 43 | 749 | 57.8 | 43 | 0 |
| 6538_supercontig | 827 | 43 | 431 | 52.1 | 43 | 0 |
| 6540_supercontig | 895 | 43 | 481 | 53.7 | 41 | 2 |
| 6544_supercontig | 1816 | 41 | 985 | 54.2 | 30 | 11 |
| 6550_supercontig | 377 | 26 | 186 | 49.3 | 26 | 0 |
| 6552_supercontig | 224 | 30 | 89 | 39.7 | 17 | 13 |
| 6557_supercontig | 731 | 42 | 379 | 51.8 | 35 | 7 |
| 6559_supercontig | 419 | 25 | 220 | 52.5 | 23 | 2 |
| 6563_supercontig | 1386 | 43 | 926 | 66.8 | 43 | 0 |
| 6570_supercontig | 192 | 12 | 160 | 83.3 | 12 | 0 |
| 6572_supercontig | 1767 | 43 | 748 | 42.3 | 42 | 1 |
| 6601_supercontig | 2573 | 43 | 1496 | 58.1 | 42 | 1 |
| 6620_supercontig | 653 | 43 | 311 | 47.6 | 43 | 0 |
| 6631_supercontig | 517 | 35 | 194 | 37.5 | 28 | 7 |
| 6636_supercontig | 1250 | 43 | 663 | 53.0 | 43 | 0 |
| 6639_supercontig | 1307 | 43 | 696 | 53.3 | 42 | 1 |
| 6641_supercontig | 1115 | 43 | 613 | 55.0 | 43 | 0 |
| 6649_supercontig | 617 | 43 | 426 | 69.0 | 40 | 3 |
| 6652_supercontig | 1782 | 43 | 849 | 47.6 | 43 | 0 |
| 6660_supercontig | 1018 | 40 | 327 | 32.1 | 38 | 2 |
| 6667_supercontig | 1736 | 43 | 830 | 47.8 | 40 | 3 |
| 6679_supercontig | 305 | 19 | 188 | 61.6 | 16 | 3 |
| 6685_supercontig | 1964 | 43 | 1193 | 60.7 | 39 | 4 |
| 6689_supercontig | 1858 | 43 | 982 | 52.9 | 43 | 0 |
| 6713_supercontig | 776 | 27 | 324 | 41.8 | 24 | 3 |
| 6717_supercontig | 1690 | 43 | 773 | 45.7 | 43 | 0 |
| 6733_supercontig | 414 | 38 | 211 | 51.0 | 32 | 6 |
| 6738_supercontig | 353 | 40 | 272 | 77.1 | 38 | 2 |
| 6746_supercontig | 930 | 43 | 671 | 72.2 | 39 | 4 |
| 6779_supercontig | 1246 | 43 | 561 | 45.0 | 40 | 3 |
| 6780_supercontig | 529 | 42 | 219 | 41.4 | 38 | 4 |
| 6782_supercontig | 978 | 43 | 604 | 61.8 | 42 | 1 |
| 6791_supercontig | 216 | 23 | 130 | 60.2 | 23 | 0 |
| 6792_supercontig | 556 | 43 | 314 | 56.5 | 27 | 16 |
| 6797_supercontig | 1702 | 43 | 888 | 52.2 | 37 | 6 |
| 6825_supercontig | 1325 | 43 | 658 | 49.7 | 43 | 0 |
| 6848_supercontig | 464 | 32 | 254 | 54.7 | 21 | 11 |
| 6854_supercontig | 1655 | 43 | 1231 | 74.4 | 43 | 0 |
| 6859_supercontig | 1767 | 43 | 1008 | 57.0 | 40 | 3 |
| 6860_supercontig | 481 | 40 | 178 | 37.0 | 34 | 6 |
| 6864_supercontig | 743 | 15 | 323 | 43.5 | 13 | 2 |
| 6865_supercontig | 1521 | 43 | 770 | 50.6 | 32 | 11 |
| 6875_supercontig | 877 | 43 | 322 | 36.7 | 40 | 3 |
| 6882_supercontig | 707 | 39 | 368 | 52.1 | 36 | 3 |
| 6883_supercontig | 1071 | 43 | 547 | 51.1 | 41 | 2 |
| 6886_supercontig | 226 | 19 | 102 | 45.1 | 19 | 0 |
| 6909_supercontig | 1695 | 43 | 1005 | 59.3 | 43 | 0 |
| 6913_supercontig | 2599 | 43 | 1602 | 61.6 | 40 | 3 |
| 6914_supercontig | 602 | 34 | 247 | 41.0 | 23 | 11 |
| 6924_supercontig | 2487 | 43 | 1289 | 51.8 | 43 | 0 |
| 6933_supercontig | 968 | 27 | 275 | 28.4 | 18 | 9 |
| 6946_supercontig | 906 | 43 | 557 | 61.5 | 39 | 4 |
| 6947_supercontig | 2371 | 40 | 1210 | 51.0 | 40 | 0 |
| 6954_supercontig | 719 | 35 | 407 | 56.6 | 34 | 1 |
| 6955_supercontig | 680 | 39 | 364 | 53.5 | 34 | 5 |
| 6958_supercontig | 671 | 40 | 305 | 45.5 | 32 | 8 |
| 6961_supercontig | 1249 | 43 | 595 | 47.6 | 40 | 3 |
| 6962_supercontig | 504 | 43 | 236 | 46.8 | 38 | 5 |
| 6968_supercontig | 1185 | 19 | 447 | 37.7 | 13 | 6 |
| 6969_supercontig | 693 | 4 | 78 | 11.3 | 4 | 0 |
| 6978_supercontig | 1849 | 43 | 955 | 51.6 | 37 | 6 |
| 6979_supercontig | 382 | 23 | 122 | 31.9 | 19 | 4 |
| 6992_supercontig | 1554 | 43 | 987 | 63.5 | 42 | 1 |
| 6995_supercontig | 310 | 17 | 169 | 54.5 | 17 | 0 |
| 7013_supercontig | 931 | 39 | 506 | 54.4 | 36 | 3 |
| 7021_supercontig | 3026 | 43 | 1687 | 55.8 | 42 | 1 |
| 7024_supercontig | 570 | 6 | 53 | 9.3 | 5 | 1 |
| 7028_supercontig | 232 | 9 | 64 | 27.6 | 9 | 0 |
| 7029_supercontig | 1431 | 43 | 761 | 53.2 | 39 | 4 |
| 7067_supercontig | 874 | 27 | 305 | 34.9 | 24 | 3 |
| 7111_supercontig | 718 | 41 | 363 | 50.6 | 34 | 7 |
| 7128_supercontig | 976 | 41 | 385 | 39.4 | 37 | 4 |
| 7135_supercontig | 377 | 14 | 222 | 58.9 | 12 | 2 |
| 7136_supercontig | 959 | 43 | 485 | 50.6 | 37 | 6 |
| 7141_supercontig | 697 | 43 | 374 | 53.7 | 42 | 1 |
| 7174_supercontig | 324 | 28 | 172 | 53.1 | 26 | 2 |
| 7194_supercontig | 904 | 42 | 471 | 52.1 | 42 | 0 |
| 7241_supercontig | 458 | 9 | 266 | 58.1 | 9 | 0 |
| 7273_supercontig | 190 | 16 | 105 | 55.3 | 16 | 0 |
| 7279_supercontig | 1159 | 42 | 824 | 71.1 | 38 | 4 |
| 7313_supercontig | 1637 | 43 | 854 | 52.2 | 43 | 0 |
| 7325_supercontig | 426 | 32 | 263 | 61.7 | 29 | 3 |
| 7333_supercontig | 501 | 43 | 190 | 37.9 | 42 | 1 |
| 7336_supercontig | 468 | 36 | 195 | 41.7 | 35 | 1 |
| 7361_supercontig | 361 | 36 | 217 | 60.1 | 34 | 2 |
| 7363_supercontig | 1045 | 41 | 562 | 53.8 | 39 | 2 |
| 7367_supercontig | 276 | 31 | 138 | 50.0 | 31 | 0 |
| 7371_supercontig | 321 | 34 | 175 | 54.5 | 34 | 0 |
| 7572_supercontig | 1986 | 43 | 1056 | 53.2 | 43 | 0 |
| 7577_supercontig | 457 | 38 | 226 | 49.5 | 37 | 1 |
| 7583_supercontig | 472 | 33 | 145 | 30.7 | 29 | 4 |
| 7602_supercontig | 961 | 43 | 462 | 48.1 | 43 | 0 |
| 7628_supercontig | 795 | 35 | 290 | 36.5 | 31 | 4 |

Table S6. Sequence recovery and variation statistics at the 70% taxon completeness threshold.

| **Gene** | **Original Length** | **Original Sequences** | **Variable Sites** | **Variable percentage** | **Filtered sequences** | **Removed sequences** |
| --- | --- | --- | --- | --- | --- | --- |
| 4471_supercontig | 1726 | 43 | 1051 | 60.9 | 43 | 0 |
| 4527_supercontig | 1635 | 43 | 797 | 48.7 | 43 | 0 |
| 4691_supercontig | 902 | 43 | 496 | 55.0 | 43 | 0 |
| 4724_supercontig | 607 | 39 | 345 | 56.8 | 39 | 0 |
| 4744_supercontig | 244 | 27 | 113 | 46.3 | 27 | 0 |
| 4757_supercontig | 1561 | 43 | 1001 | 64.1 | 43 | 0 |
| 4793_supercontig | 826 | 39 | 656 | 79.4 | 33 | 6 |
| 4796_supercontig | 1248 | 43 | 811 | 65.0 | 43 | 0 |
| 4802_supercontig | 2027 | 43 | 938 | 46.3 | 43 | 0 |
| 4806_supercontig | 664 | 28 | 307 | 46.2 | 25 | 3 |
| 4848_supercontig | 1816 | 43 | 659 | 36.3 | 43 | 0 |
| 4889_supercontig | 842 | 43 | 509 | 60.5 | 42 | 1 |
| 4890_supercontig | 548 | 16 | 311 | 56.8 | 16 | 0 |
| 4893_supercontig | 1556 | 43 | 873 | 56.1 | 43 | 0 |
| 4932_supercontig | 1341 | 43 | 597 | 44.5 | 43 | 0 |
| 4942_supercontig | 2014 | 43 | 1106 | 54.9 | 43 | 0 |
| 4951_supercontig | 709 | 43 | 391 | 55.1 | 43 | 0 |
| 4954_supercontig | 939 | 43 | 458 | 48.8 | 43 | 0 |
| 4989_supercontig | 1141 | 43 | 488 | 42.8 | 43 | 0 |
| 4992_supercontig | 978 | 43 | 505 | 51.6 | 40 | 3 |
| 5018_supercontig | 1786 | 43 | 885 | 49.6 | 43 | 0 |
| 5032_supercontig | 573 | 35 | 234 | 40.8 | 35 | 0 |
| 5034_supercontig | 1524 | 43 | 783 | 51.4 | 43 | 0 |
| 5038_supercontig | 1023 | 43 | 570 | 55.7 | 43 | 0 |
| 5064_supercontig | 262 | 24 | 228 | 87.0 | 24 | 0 |
| 5090_supercontig | 1833 | 43 | 1176 | 64.2 | 43 | 0 |
| 5104_supercontig | 985 | 4 | 263 | 26.7 | 4 | 0 |
| 5116_supercontig | 1128 | 42 | 579 | 51.3 | 34 | 8 |
| 5123_supercontig | 1155 | 43 | 891 | 77.1 | 43 | 0 |
| 5131_supercontig | 354 | 27 | 181 | 51.1 | 27 | 0 |
| 5138_supercontig | 1407 | 43 | 678 | 48.2 | 42 | 1 |
| 5162_supercontig | 1048 | 43 | 376 | 35.9 | 43 | 0 |
| 5163_supercontig | 2485 | 43 | 1186 | 47.7 | 43 | 0 |
| 5168_supercontig | 706 | 24 | 272 | 38.5 | 18 | 6 |
| 5177_supercontig | 358 | 40 | 158 | 44.1 | 40 | 0 |
| 5188_supercontig | 1528 | 43 | 1115 | 73.0 | 43 | 0 |
| 5200_supercontig | 1369 | 43 | 851 | 62.2 | 43 | 0 |
| 5206_supercontig | 1131 | 43 | 527 | 46.6 | 43 | 0 |
| 5220_supercontig | 732 | 42 | 337 | 46.0 | 42 | 0 |
| 5257_supercontig | 1235 | 43 | 567 | 45.9 | 43 | 0 |
| 5264_supercontig | 1484 | 43 | 863 | 58.2 | 43 | 0 |
| 5271_supercontig | 497 | 4 | 149 | 30.0 | 4 | 0 |
| 5273_supercontig | 412 | 29 | 189 | 45.9 | 28 | 1 |
| 5280_supercontig | 1757 | 42 | 756 | 43.0 | 42 | 0 |
| 5296_supercontig | 2295 | 43 | 1079 | 47.0 | 43 | 0 |
| 5299_supercontig | 1019 | 42 | 559 | 54.9 | 41 | 1 |
| 5304_supercontig | 2024 | 43 | 964 | 47.6 | 43 | 0 |
| 5318_supercontig | 1479 | 43 | 760 | 51.4 | 43 | 0 |
| 5326_supercontig | 1958 | 41 | 887 | 45.3 | 41 | 0 |
| 5328_supercontig | 779 | 43 | 384 | 49.3 | 43 | 0 |
| 5333_supercontig | 371 | 21 | 262 | 70.6 | 20 | 1 |
| 5335_supercontig | 1110 | 43 | 625 | 56.3 | 42 | 1 |
| 5339_supercontig | 1167 | 43 | 641 | 54.9 | 41 | 2 |
| 5343_supercontig | 900 | 43 | 375 | 41.7 | 43 | 0 |
| 5347_supercontig | 824 | 42 | 520 | 63.1 | 41 | 1 |
| 5348_supercontig | 448 | 35 | 202 | 45.1 | 35 | 0 |
| 5354_supercontig | 1303 | 42 | 721 | 55.3 | 41 | 1 |
| 5355_supercontig | 975 | 43 | 405 | 41.5 | 43 | 0 |
| 5357_supercontig | 527 | 32 | 285 | 54.1 | 32 | 0 |
| 5366_supercontig | 1806 | 43 | 1011 | 56.0 | 43 | 0 |
| 5398_supercontig | 2539 | 43 | 1237 | 48.7 | 43 | 0 |
| 5404_supercontig | 1178 | 36 | 607 | 51.5 | 32 | 4 |
| 5406_supercontig | 2757 | 43 | 1330 | 48.2 | 43 | 0 |
| 5421_supercontig | 751 | 43 | 372 | 49.5 | 43 | 0 |
| 5422_supercontig | 896 | 40 | 463 | 51.7 | 38 | 2 |
| 5426_supercontig | 2381 | 43 | 1124 | 47.2 | 43 | 0 |
| 5427_supercontig | 473 | 23 | 303 | 64.1 | 21 | 2 |
| 5428_supercontig | 699 | 43 | 284 | 40.6 | 43 | 0 |
| 5430_supercontig | 1192 | 32 | 714 | 59.9 | 24 | 8 |
| 5434_supercontig | 1372 | 43 | 857 | 62.5 | 43 | 0 |
| 5449_supercontig | 2271 | 43 | 1333 | 58.7 | 43 | 0 |
| 5454_supercontig | 3341 | 43 | 1592 | 47.7 | 43 | 0 |
| 5460_supercontig | 1015 | 43 | 597 | 58.8 | 43 | 0 |
| 5463_supercontig | 861 | 43 | 561 | 65.2 | 43 | 0 |
| 5464_supercontig | 1302 | 43 | 878 | 67.4 | 41 | 2 |
| 5469_supercontig | 1019 | 43 | 659 | 64.7 | 42 | 1 |
| 5477_supercontig | 645 | 39 | 418 | 64.8 | 35 | 4 |
| 5489_supercontig | 2041 | 43 | 1152 | 56.4 | 43 | 0 |
| 5502_supercontig | 2784 | 43 | 1511 | 54.3 | 43 | 0 |
| 5513_supercontig | 875 | 40 | 570 | 65.1 | 38 | 2 |
| 5528_supercontig | 4019 | 43 | 2090 | 52.0 | 43 | 0 |
| 5531_supercontig | 468 | 39 | 203 | 43.4 | 39 | 0 |
| 5536_supercontig | 784 | 31 | 365 | 46.6 | 23 | 8 |
| 5551_supercontig | 2053 | 42 | 1105 | 53.8 | 42 | 0 |
| 5554_supercontig | 1773 | 43 | 1120 | 63.2 | 43 | 0 |
| 5562_supercontig | 408 | 36 | 272 | 66.7 | 36 | 0 |
| 5578_supercontig | 232 | 16 | 66 | 28.4 | 16 | 0 |
| 5594_supercontig | 1039 | 43 | 551 | 53.0 | 43 | 0 |
| 5596_supercontig | 2244 | 42 | 729 | 32.5 | 42 | 0 |
| 5599_supercontig | 3588 | 43 | 1848 | 51.5 | 43 | 0 |
| 5614_supercontig | 2452 | 42 | 1251 | 51.0 | 42 | 0 |
| 5620_supercontig | 1156 | 43 | 483 | 41.8 | 43 | 0 |
| 5634_supercontig | 3071 | 42 | 1748 | 56.9 | 42 | 0 |
| 5639_supercontig | 888 | 40 | 443 | 49.9 | 34 | 6 |
| 5642_supercontig | 243 | 26 | 143 | 58.8 | 25 | 1 |
| 5644_supercontig | 2081 | 43 | 1193 | 57.3 | 43 | 0 |
| 5656_supercontig | 776 | 42 | 325 | 41.9 | 41 | 1 |
| 5660_supercontig | 527 | 36 | 148 | 28.1 | 36 | 0 |
| 5664_supercontig | 1747 | 43 | 962 | 55.1 | 43 | 0 |
| 5670_supercontig | 3918 | 43 | 1665 | 42.5 | 43 | 0 |
| 5699_supercontig | 383 | 36 | 273 | 71.3 | 36 | 0 |
| 5702_supercontig | 1250 | 43 | 536 | 42.9 | 36 | 7 |
| 5703_supercontig | 1338 | 41 | 689 | 51.5 | 39 | 2 |
| 5716_supercontig | 1934 | 43 | 1357 | 70.2 | 43 | 0 |
| 5721_supercontig | 1336 | 43 | 751 | 56.2 | 41 | 2 |
| 5733_supercontig | 2328 | 42 | 1415 | 60.8 | 41 | 1 |
| 5744_supercontig | 774 | 42 | 585 | 75.6 | 42 | 0 |
| 5770_supercontig | 877 | 43 | 522 | 59.5 | 42 | 1 |
| 5772_supercontig | 1187 | 43 | 710 | 59.8 | 43 | 0 |
| 5791_supercontig | 697 | 26 | 437 | 62.7 | 26 | 0 |
| 5802_supercontig | 1087 | 43 | 464 | 42.7 | 43 | 0 |
| 5815_supercontig | 768 | 42 | 461 | 60.0 | 41 | 1 |
| 5816_supercontig | 278 | 31 | 136 | 48.9 | 30 | 1 |
| 5821_supercontig | 926 | 41 | 524 | 56.6 | 40 | 1 |
| 5822_supercontig | 731 | 20 | 278 | 38.0 | 20 | 0 |
| 5840_supercontig | 1823 | 43 | 950 | 52.1 | 43 | 0 |
| 5841_supercontig | 1061 | 41 | 538 | 50.7 | 39 | 2 |
| 5842_supercontig | 575 | 41 | 214 | 37.2 | 41 | 0 |
| 5843_supercontig | 535 | 15 | 315 | 58.9 | 15 | 0 |
| 5849_supercontig | 894 | 42 | 415 | 46.4 | 42 | 0 |
| 5853_supercontig | 855 | 43 | 357 | 41.8 | 40 | 3 |
| 5857_supercontig | 1693 | 43 | 954 | 56.3 | 43 | 0 |
| 5858_supercontig | 1382 | 42 | 786 | 56.9 | 42 | 0 |
| 5859_supercontig | 1155 | 43 | 642 | 55.6 | 43 | 0 |
| 5865_supercontig | 413 | 41 | 209 | 50.6 | 40 | 1 |
| 5866_supercontig | 1005 | 43 | 471 | 46.9 | 43 | 0 |
| 5870_supercontig | 2102 | 43 | 1330 | 63.3 | 43 | 0 |
| 5893_supercontig | 556 | 28 | 271 | 48.7 | 28 | 0 |
| 5894_supercontig | 1397 | 43 | 932 | 66.7 | 43 | 0 |
| 5899_supercontig | 2096 | 43 | 1146 | 54.7 | 43 | 0 |
| 5910_supercontig | 1502 | 43 | 710 | 47.3 | 43 | 0 |
| 5913_supercontig | 1584 | 43 | 770 | 48.6 | 43 | 0 |
| 5918_supercontig | 761 | 40 | 424 | 55.7 | 35 | 5 |
| 5919_supercontig | 488 | 41 | 220 | 45.1 | 41 | 0 |
| 5921_supercontig | 6320 | 43 | 3326 | 52.6 | 42 | 1 |
| 5922_supercontig | 3217 | 43 | 1625 | 50.5 | 43 | 0 |
| 5926_supercontig | 1052 | 43 | 640 | 60.8 | 43 | 0 |
| 5933_supercontig | 1287 | 43 | 656 | 51.0 | 43 | 0 |
| 5936_supercontig | 1297 | 42 | 828 | 63.8 | 42 | 0 |
| 5940_supercontig | 1242 | 43 | 808 | 65.1 | 42 | 1 |
| 5941_supercontig | 881 | 42 | 667 | 75.7 | 41 | 1 |
| 5942_supercontig | 943 | 43 | 463 | 49.1 | 43 | 0 |
| 5943_supercontig | 4021 | 41 | 1944 | 48.3 | 41 | 0 |
| 5944_supercontig | 1066 | 43 | 684 | 64.2 | 43 | 0 |
| 5945_supercontig | 1931 | 43 | 1306 | 67.6 | 43 | 0 |
| 5949_supercontig | 1378 | 43 | 839 | 60.9 | 43 | 0 |
| 5950_supercontig | 1124 | 42 | 676 | 60.1 | 42 | 0 |
| 5958_supercontig | 482 | 40 | 184 | 38.2 | 40 | 0 |
| 5960_supercontig | 1311 | 43 | 655 | 50.0 | 43 | 0 |
| 5968_supercontig | 694 | 43 | 372 | 53.6 | 43 | 0 |
| 5974_supercontig | 1519 | 43 | 697 | 45.9 | 43 | 0 |
| 5977_supercontig | 677 | 42 | 243 | 35.9 | 41 | 1 |
| 5980_supercontig | 1114 | 43 | 682 | 61.2 | 42 | 1 |
| 5981_supercontig | 258 | 19 | 136 | 52.7 | 18 | 1 |
| 5990_supercontig | 216 | 24 | 154 | 71.3 | 24 | 0 |
| 6000_supercontig | 200 | 13 | 95 | 47.5 | 12 | 1 |
| 6003_supercontig | 794 | 30 | 498 | 62.7 | 30 | 0 |
| 6004_supercontig | 1103 | 43 | 478 | 43.3 | 43 | 0 |
| 6016_supercontig | 1717 | 43 | 949 | 55.3 | 43 | 0 |
| 6026_supercontig | 928 | 43 | 457 | 49.2 | 40 | 3 |
| 6029_supercontig | 1276 | 43 | 651 | 51.0 | 43 | 0 |
| 6034_supercontig | 1098 | 37 | 639 | 58.2 | 37 | 0 |
| 6036_supercontig | 2172 | 35 | 1042 | 48.0 | 35 | 0 |
| 6038_supercontig | 2275 | 43 | 1374 | 60.4 | 43 | 0 |
| 6041_supercontig | 2191 | 43 | 1097 | 50.1 | 43 | 0 |
| 6048_supercontig | 971 | 43 | 550 | 56.6 | 43 | 0 |
| 6050_supercontig | 729 | 32 | 459 | 63.0 | 32 | 0 |
| 6051_supercontig | 1365 | 43 | 689 | 50.5 | 43 | 0 |
| 6056_supercontig | 782 | 41 | 407 | 52.0 | 39 | 2 |
| 6064_supercontig | 1412 | 43 | 702 | 49.7 | 43 | 0 |
| 6068_supercontig | 988 | 43 | 564 | 57.1 | 43 | 0 |
| 6098_supercontig | 2753 | 43 | 1315 | 47.8 | 43 | 0 |
| 6110_supercontig | 499 | 39 | 212 | 42.5 | 39 | 0 |
| 6114_supercontig | 1704 | 43 | 962 | 56.5 | 42 | 1 |
| 6119_supercontig | 2041 | 43 | 1109 | 54.3 | 43 | 0 |
| 6128_supercontig | 1826 | 42 | 966 | 52.9 | 42 | 0 |
| 6130_supercontig | 1409 | 43 | 696 | 49.4 | 43 | 0 |
| 6139_supercontig | 2024 | 43 | 1043 | 51.5 | 43 | 0 |
| 6148_supercontig | 461 | 8 | 126 | 27.3 | 8 | 0 |
| 6150_supercontig | 191 | 23 | 59 | 30.9 | 22 | 1 |
| 6164_supercontig | 791 | 39 | 420 | 53.1 | 38 | 1 |
| 6176_supercontig | 1769 | 43 | 894 | 50.5 | 43 | 0 |
| 6198_supercontig | 780 | 24 | 589 | 75.5 | 21 | 3 |
| 6216_supercontig | 1283 | 43 | 595 | 46.4 | 38 | 5 |
| 6221_supercontig | 237 | 17 | 111 | 46.8 | 16 | 1 |
| 6226_supercontig | 995 | 43 | 498 | 50.1 | 43 | 0 |
| 6227_supercontig | 1691 | 43 | 938 | 55.5 | 43 | 0 |
| 6238_supercontig | 1262 | 43 | 567 | 44.9 | 43 | 0 |
| 6258_supercontig | 1058 | 43 | 722 | 68.2 | 42 | 1 |
| 6265_supercontig | 2001 | 42 | 1046 | 52.3 | 41 | 1 |
| 6270_supercontig | 916 | 16 | 507 | 55.3 | 15 | 1 |
| 6274_supercontig | 948 | 43 | 543 | 57.3 | 43 | 0 |
| 6282_supercontig | 1586 | 43 | 780 | 49.2 | 43 | 0 |
| 6284_supercontig | 1565 | 43 | 796 | 50.9 | 43 | 0 |
| 6295_supercontig | 1957 | 43 | 1159 | 59.2 | 43 | 0 |
| 6298_supercontig | 1066 | 43 | 582 | 54.6 | 43 | 0 |
| 6299_supercontig | 599 | 43 | 301 | 50.3 | 43 | 0 |
| 6303_supercontig | 1070 | 43 | 630 | 58.9 | 42 | 1 |
| 6318_supercontig | 1268 | 43 | 766 | 60.4 | 40 | 3 |
| 6320_supercontig | 2127 | 43 | 1144 | 53.8 | 42 | 1 |
| 6363_supercontig | 1644 | 43 | 949 | 57.7 | 43 | 0 |
| 6366_supercontig | 309 | 25 | 210 | 68.0 | 25 | 0 |
| 6373_supercontig | 848 | 42 | 504 | 59.4 | 42 | 0 |
| 6376_supercontig | 981 | 43 | 513 | 52.3 | 41 | 2 |
| 6378_supercontig | 1269 | 43 | 592 | 46.7 | 41 | 2 |
| 6379_supercontig | 980 | 42 | 616 | 62.9 | 40 | 2 |
| 6383_supercontig | 1888 | 43 | 974 | 51.6 | 43 | 0 |
| 6384_supercontig | 311 | 39 | 259 | 83.3 | 28 | 11 |
| 6387_supercontig | 959 | 43 | 461 | 48.1 | 43 | 0 |
| 6389_supercontig | 1553 | 33 | 866 | 55.8 | 33 | 0 |
| 6393_supercontig | 1005 | 43 | 591 | 58.8 | 43 | 0 |
| 6398_supercontig | 227 | 9 | 121 | 53.3 | 9 | 0 |
| 6401_supercontig | 700 | 43 | 342 | 48.9 | 43 | 0 |
| 6404_supercontig | 899 | 43 | 489 | 54.4 | 43 | 0 |
| 6405_supercontig | 1227 | 43 | 749 | 61.0 | 43 | 0 |
| 6406_supercontig | 340 | 7 | 201 | 59.1 | 7 | 0 |
| 6412_supercontig | 1182 | 43 | 523 | 44.2 | 43 | 0 |
| 6420_supercontig | 1396 | 42 | 659 | 47.2 | 42 | 0 |
| 6432_supercontig | 655 | 38 | 314 | 47.9 | 38 | 0 |
| 6439_supercontig | 835 | 43 | 470 | 56.3 | 40 | 3 |
| 6447_supercontig | 829 | 35 | 531 | 64.1 | 35 | 0 |
| 6448_supercontig | 836 | 39 | 411 | 49.2 | 39 | 0 |
| 6450_supercontig | 1504 | 43 | 775 | 51.5 | 43 | 0 |
| 6454_supercontig | 798 | 42 | 456 | 57.1 | 42 | 0 |
| 6457_supercontig | 228 | 30 | 86 | 37.7 | 30 | 0 |
| 6458_supercontig | 749 | 28 | 506 | 67.6 | 27 | 1 |
| 6459_supercontig | 1379 | 43 | 848 | 61.5 | 41 | 2 |
| 6460_supercontig | 393 | 41 | 244 | 62.1 | 41 | 0 |
| 6462_supercontig | 1778 | 43 | 954 | 53.7 | 43 | 0 |
| 6483_supercontig | 1108 | 43 | 620 | 56.0 | 43 | 0 |
| 6487_supercontig | 2775 | 43 | 1327 | 47.8 | 43 | 0 |
| 6488_supercontig | 1058 | 42 | 495 | 46.8 | 39 | 3 |
| 6492_supercontig | 1107 | 43 | 571 | 51.6 | 43 | 0 |
| 6494_supercontig | 1609 | 43 | 690 | 42.9 | 43 | 0 |
| 6496_supercontig | 2870 | 43 | 1647 | 57.4 | 43 | 0 |
| 6498_supercontig | 936 | 43 | 599 | 64.0 | 43 | 0 |
| 6500_supercontig | 2468 | 43 | 1258 | 51.0 | 43 | 0 |
| 6506_supercontig | 1257 | 43 | 866 | 68.9 | 40 | 3 |
| 6507_supercontig | 310 | 12 | 103 | 33.2 | 12 | 0 |
| 6527_supercontig | 2223 | 43 | 1094 | 49.2 | 43 | 0 |
| 6528_supercontig | 963 | 42 | 418 | 43.4 | 41 | 1 |
| 6531_supercontig | 784 | 42 | 469 | 59.8 | 42 | 0 |
| 6532_supercontig | 691 | 43 | 406 | 58.8 | 42 | 1 |
| 6533_supercontig | 1296 | 43 | 749 | 57.8 | 43 | 0 |
| 6538_supercontig | 827 | 43 | 431 | 52.1 | 43 | 0 |
| 6540_supercontig | 895 | 43 | 481 | 53.7 | 43 | 0 |
| 6544_supercontig | 1816 | 41 | 985 | 54.2 | 35 | 6 |
| 6550_supercontig | 377 | 26 | 186 | 49.3 | 26 | 0 |
| 6552_supercontig | 224 | 30 | 89 | 39.7 | 17 | 13 |
| 6557_supercontig | 731 | 42 | 379 | 51.8 | 41 | 1 |
| 6559_supercontig | 419 | 25 | 220 | 52.5 | 25 | 0 |
| 6563_supercontig | 1386 | 43 | 926 | 66.8 | 43 | 0 |
| 6570_supercontig | 192 | 12 | 160 | 83.3 | 12 | 0 |
| 6572_supercontig | 1767 | 43 | 748 | 42.3 | 43 | 0 |
| 6601_supercontig | 2573 | 43 | 1496 | 58.1 | 43 | 0 |
| 6620_supercontig | 653 | 43 | 311 | 47.6 | 43 | 0 |
| 6631_supercontig | 517 | 35 | 194 | 37.5 | 34 | 1 |
| 6636_supercontig | 1250 | 43 | 663 | 53.0 | 43 | 0 |
| 6639_supercontig | 1307 | 43 | 696 | 53.3 | 43 | 0 |
| 6641_supercontig | 1115 | 43 | 613 | 55.0 | 43 | 0 |
| 6649_supercontig | 617 | 43 | 426 | 69.0 | 43 | 0 |
| 6652_supercontig | 1782 | 43 | 849 | 47.6 | 43 | 0 |
| 6660_supercontig | 1018 | 40 | 327 | 32.1 | 38 | 2 |
| 6667_supercontig | 1736 | 43 | 830 | 47.8 | 43 | 0 |
| 6679_supercontig | 305 | 19 | 188 | 61.6 | 17 | 2 |
| 6685_supercontig | 1964 | 43 | 1193 | 60.7 | 43 | 0 |
| 6689_supercontig | 1858 | 43 | 982 | 52.9 | 43 | 0 |
| 6713_supercontig | 776 | 27 | 324 | 41.8 | 25 | 2 |
| 6717_supercontig | 1690 | 43 | 773 | 45.7 | 43 | 0 |
| 6733_supercontig | 414 | 38 | 211 | 51.0 | 38 | 0 |
| 6738_supercontig | 353 | 40 | 272 | 77.1 | 39 | 1 |
| 6746_supercontig | 930 | 43 | 671 | 72.2 | 43 | 0 |
| 6779_supercontig | 1246 | 43 | 561 | 45.0 | 42 | 1 |
| 6780_supercontig | 529 | 42 | 219 | 41.4 | 42 | 0 |
| 6782_supercontig | 978 | 43 | 604 | 61.8 | 43 | 0 |
| 6791_supercontig | 216 | 23 | 130 | 60.2 | 23 | 0 |
| 6792_supercontig | 556 | 43 | 314 | 56.5 | 42 | 1 |
| 6797_supercontig | 1702 | 43 | 888 | 52.2 | 42 | 1 |
| 6825_supercontig | 1325 | 43 | 658 | 49.7 | 43 | 0 |
| 6848_supercontig | 464 | 32 | 254 | 54.7 | 26 | 6 |
| 6854_supercontig | 1655 | 43 | 1231 | 74.4 | 43 | 0 |
| 6859_supercontig | 1767 | 43 | 1008 | 57.0 | 43 | 0 |
| 6860_supercontig | 481 | 40 | 178 | 37.0 | 40 | 0 |
| 6864_supercontig | 743 | 15 | 323 | 43.5 | 15 | 0 |
| 6865_supercontig | 1521 | 43 | 770 | 50.6 | 43 | 0 |
| 6875_supercontig | 877 | 43 | 322 | 36.7 | 43 | 0 |
| 6882_supercontig | 707 | 39 | 368 | 52.1 | 37 | 2 |
| 6883_supercontig | 1071 | 43 | 547 | 51.1 | 43 | 0 |
| 6886_supercontig | 226 | 19 | 102 | 45.1 | 19 | 0 |
| 6909_supercontig | 1695 | 43 | 1005 | 59.3 | 43 | 0 |
| 6913_supercontig | 2599 | 43 | 1602 | 61.6 | 43 | 0 |
| 6914_supercontig | 602 | 34 | 247 | 41.0 | 34 | 0 |
| 6924_supercontig | 2487 | 43 | 1289 | 51.8 | 43 | 0 |
| 6933_supercontig | 968 | 27 | 275 | 28.4 | 20 | 7 |
| 6946_supercontig | 906 | 43 | 557 | 61.5 | 41 | 2 |
| 6947_supercontig | 2371 | 40 | 1210 | 51.0 | 40 | 0 |
| 6954_supercontig | 719 | 35 | 407 | 56.6 | 35 | 0 |
| 6955_supercontig | 680 | 39 | 364 | 53.5 | 39 | 0 |
| 6958_supercontig | 671 | 40 | 305 | 45.5 | 38 | 2 |
| 6961_supercontig | 1249 | 43 | 595 | 47.6 | 40 | 3 |
| 6962_supercontig | 504 | 43 | 236 | 46.8 | 43 | 0 |
| 6968_supercontig | 1185 | 19 | 447 | 37.7 | 16 | 3 |
| 6969_supercontig | 693 | 4 | 78 | 11.3 | 4 | 0 |
| 6978_supercontig | 1849 | 43 | 955 | 51.6 | 43 | 0 |
| 6979_supercontig | 382 | 23 | 122 | 31.9 | 20 | 3 |
| 6992_supercontig | 1554 | 43 | 987 | 63.5 | 42 | 1 |
| 6995_supercontig | 310 | 17 | 169 | 54.5 | 17 | 0 |
| 7013_supercontig | 931 | 39 | 506 | 54.4 | 37 | 2 |
| 7021_supercontig | 3026 | 43 | 1687 | 55.8 | 43 | 0 |
| 7024_supercontig | 570 | 6 | 53 | 9.3 | 6 | 0 |
| 7028_supercontig | 232 | 9 | 64 | 27.6 | 9 | 0 |
| 7029_supercontig | 1431 | 43 | 761 | 53.2 | 43 | 0 |
| 7067_supercontig | 874 | 27 | 305 | 34.9 | 26 | 1 |
| 7111_supercontig | 718 | 41 | 363 | 50.6 | 40 | 1 |
| 7128_supercontig | 976 | 41 | 385 | 39.4 | 39 | 2 |
| 7135_supercontig | 377 | 14 | 222 | 58.9 | 14 | 0 |
| 7136_supercontig | 959 | 43 | 485 | 50.6 | 39 | 4 |
| 7141_supercontig | 697 | 43 | 374 | 53.7 | 43 | 0 |
| 7174_supercontig | 324 | 28 | 172 | 53.1 | 27 | 1 |
| 7194_supercontig | 904 | 42 | 471 | 52.1 | 42 | 0 |
| 7241_supercontig | 458 | 9 | 266 | 58.1 | 9 | 0 |
| 7273_supercontig | 190 | 16 | 105 | 55.3 | 16 | 0 |
| 7279_supercontig | 1159 | 42 | 824 | 71.1 | 41 | 1 |
| 7313_supercontig | 1637 | 43 | 854 | 52.2 | 43 | 0 |
| 7325_supercontig | 426 | 32 | 263 | 61.7 | 32 | 0 |
| 7331_supercontig | 535 | 5 | 296 | 55.3 | 5 | 0 |
| 7333_supercontig | 501 | 43 | 190 | 37.9 | 43 | 0 |
| 7336_supercontig | 468 | 36 | 195 | 41.7 | 36 | 0 |
| 7361_supercontig | 361 | 36 | 217 | 60.1 | 36 | 0 |
| 7363_supercontig | 1045 | 41 | 562 | 53.8 | 41 | 0 |
| 7367_supercontig | 276 | 31 | 138 | 50.0 | 31 | 0 |
| 7371_supercontig | 321 | 34 | 175 | 54.5 | 34 | 0 |
| 7572_supercontig | 1986 | 43 | 1056 | 53.2 | 43 | 0 |
| 7577_supercontig | 457 | 38 | 226 | 49.5 | 38 | 0 |
| 7583_supercontig | 472 | 33 | 145 | 30.7 | 32 | 1 |
| 7602_supercontig | 961 | 43 | 462 | 48.1 | 43 | 0 |
| 7628_supercontig | 795 | 35 | 290 | 36.5 | 35 | 0 |


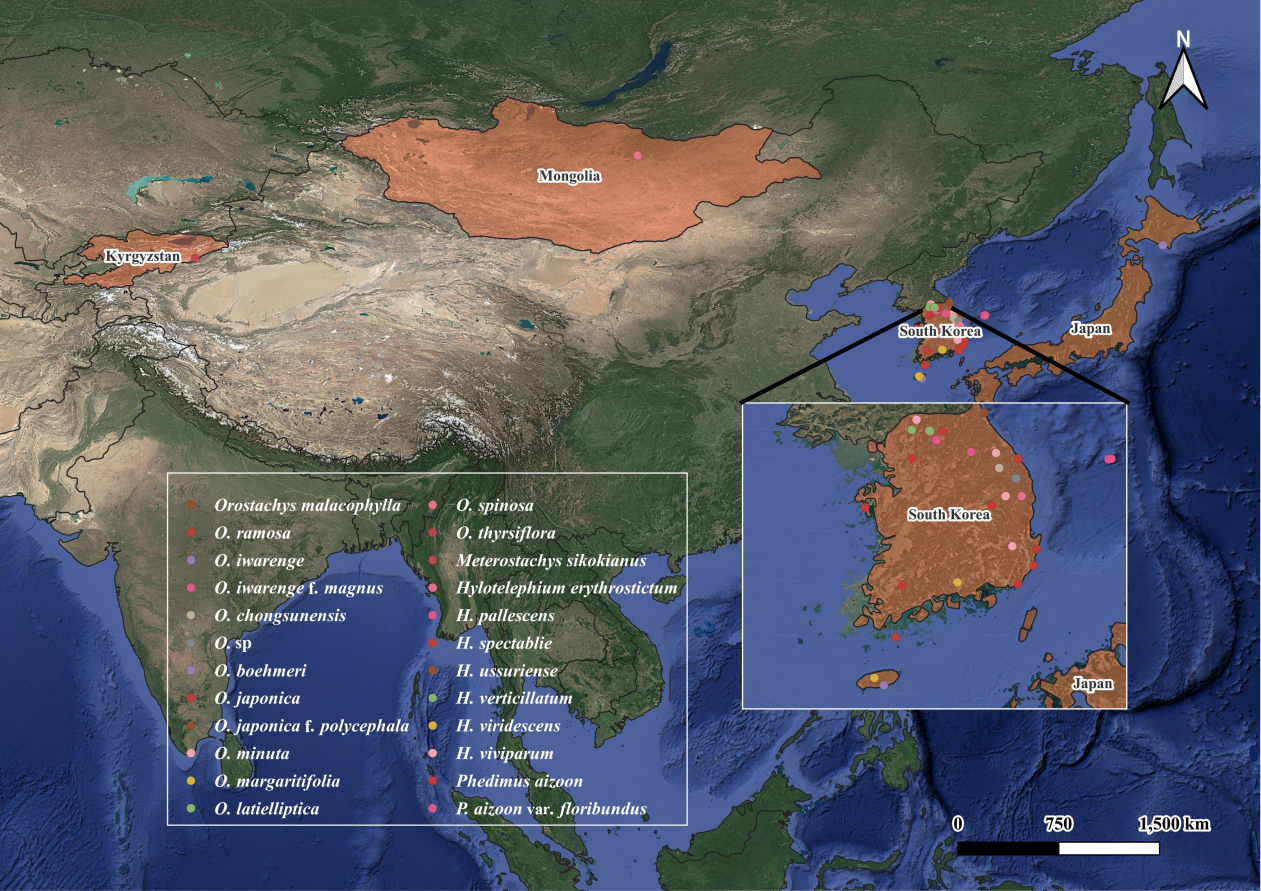


Figure S1. Map of the sampling sites used in this study.


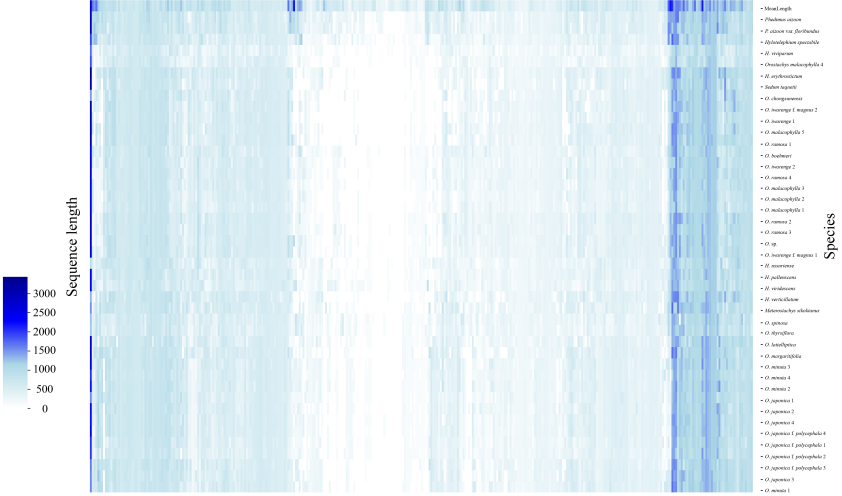


Figure S2. Heatmap of gene recovery efficiency across 43 individuals representing 25 taxa from the Angiosperms353 target enrichment. Each row represents one sample, and each column represents one gene. The colors indicate the absolute recovered sequence length in base pairs (bp).


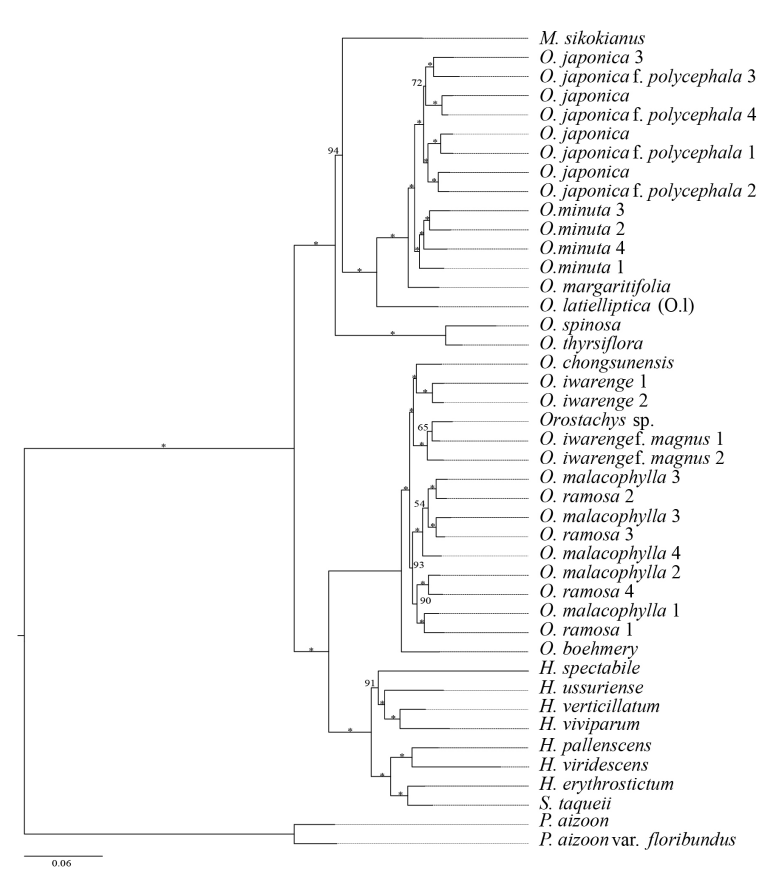


Figure S3. Concatenated maximum likelihood (ML) tree based on supercontig data filtered by a 30% completeness threshold and inferred with IQ-TREE. The numbers at nodes represent UFBoot support values. Asterisks (*) indicate 100% UFBoot support. The scale bar indicates substitutions per site.


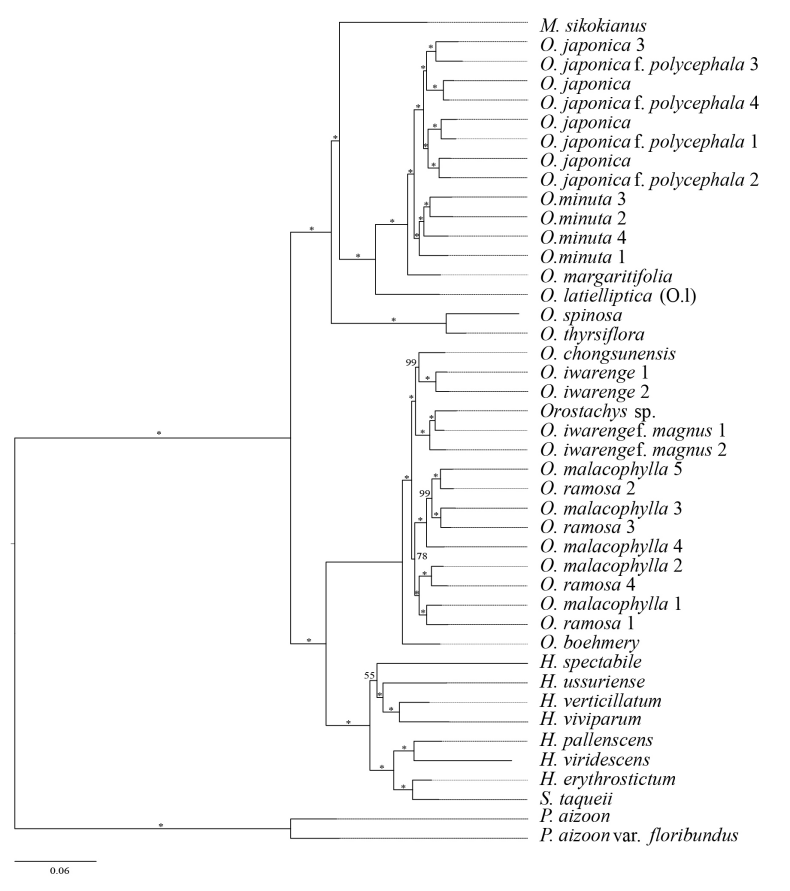


Figure S4. Concatenated maximum likelihood (ML) tree based on supercontig data filtered by a 70% completeness threshold and inferred with IQ-TREE. The numbers at the nodes represent UFBoot support values. Asterisks (*) indicate 100% UFBoot support. The scale bar indicates substitutions per site.


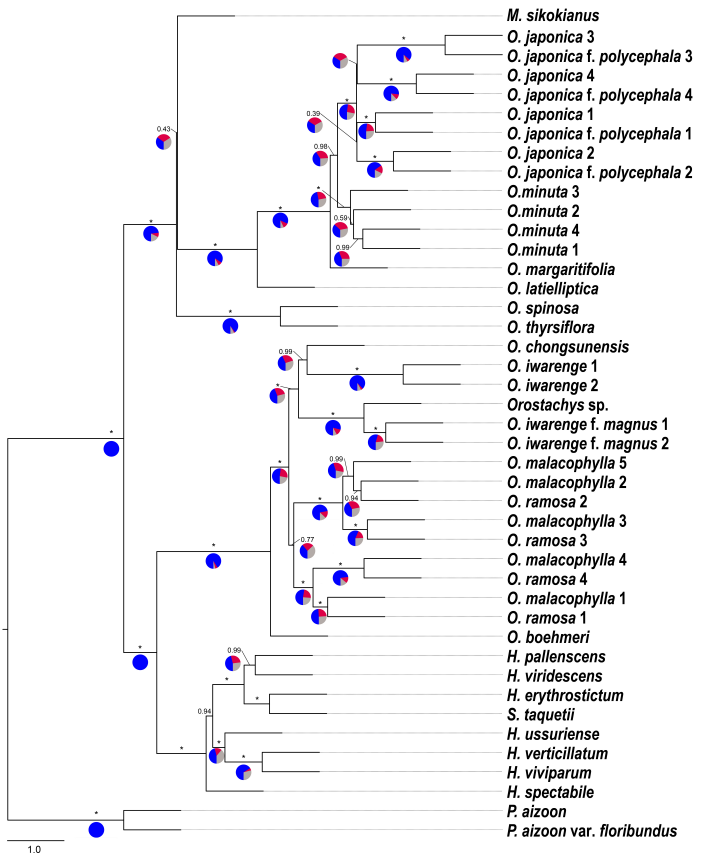


Figure S5. Multi-locus species tree constructed using data filtered by the 30% threshold. The numbers at the nodes represent LPP values. Asterisks (*) indicate 1.00 UFBoot support. The scale bar indicates coalescent units. Pie charts at each node represent the number of gene trees that fall into one of three categories: concordant with the multi-locus species tree (blue), discordant with the multi-locus species tree (red for all other alternatives), and uninformative (gray).


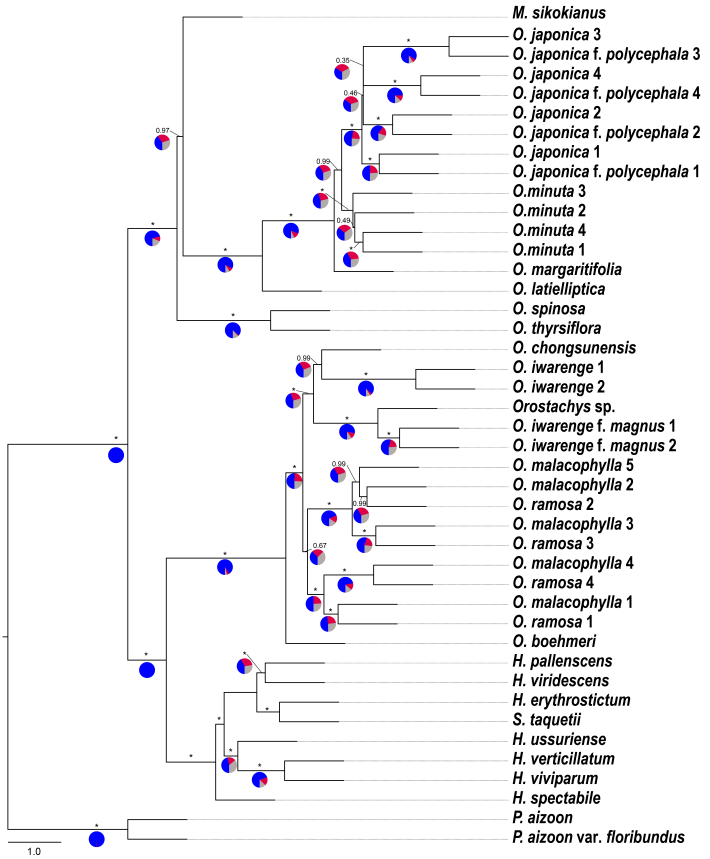


Figure S6. Multi-locus species tree constructed using data filtered by the 70% threshold. The numbers at the nodes represent LPP values. Asterisks (*) indicate 1.00 UFBoot support. The scale bar indicates coalescent units. Pie charts at each node represent the number of gene trees that fall into one of three categories: concordant with the multi-locus species tree (blue), discordant with the multi-locus species tree (red for all other alternatives), and uninformative (gray).
